# Quinazolin-4(3*H*)-one based potential multiple tyrosine kinase inhibitors with excellent cytotoxicity

**DOI:** 10.1080/14756366.2021.1972992

**Published:** 2021-09-22

**Authors:** Tebyan O. Mirgany, Ashraf N. Abdalla, Md Arifuzzaman, A. F. M. Motiur Rahman, Huda S. Al-Salem

**Affiliations:** a Department of Pharmaceutical Chemistry, College of Pharmacy, King Saud University, Riyadh, Saudi Arabia; b Department of Pharmacology and Toxicology, Faculty of Pharmacy, Umm Al-Qura University, Makkah, Saudi Arabia; c College of Pharmacy, Yeungnam University, Gyeongsan, Korea

**Keywords:** Quinazolin-4(3*H*)-one, CDK2, HER2, EGFR, drug likeness properties

## Abstract

A series of quinazolin-4(3*H*)-one derivatives were synthesised and evaluated for their cytotoxicity against human Caucasian breast adenocarcinoma (MCF-7) and human ovarian carcinoma (A2780) cell lines. Cytotoxicity of the most tested compounds was 2- to 30-fold more than the positive control lapatinib (IC_50_ of **2j =** 3.79 ± 0.96; **3j** = 0.20 ± 0.02; and lapatinib = 5.9 ± 0.74) against MCF7 cell lines except two compounds (IC_50_ of **2 b** = 15.72 ± 0.07 and **2e** = 14.88 ± 0.99). On the other hand, cytotoxicity was 4 − 87 folds (IC_50_ of **3a** = 3.00 ± 1.20; **3 g** = 0.14 ± 0.03) more the positive control lapatinib (IC_50_ = 12.11 ± 1.03) against A2780 cell lines except compound **2e** (IC_50_ = 16.43 ± 1.80). Among the synthesised quinazolin-4(3*H*)-one derivatives, potent cytotoxic **2f-j** and **3f-j** were investigated for molecular mechanism of action. Inhibitory activities of the compounds were tested against multiple tyrosine protein kinases (CDK2, HER2, EGFR and VEGFR2) enzymes. As expected, all the quinazolin-4(3*H*)-one derivatives were showed comparable inhibitory activity against those kinases tested, especially, compound **2i** and **3i** showed potent inhibitory activity against CDK2, HER2, EGFR tyrosine kinases. Therefore, molecular docking analysis for quinazolin-4(3*H*)-one derivatives **2i** and **3i** were performed, and it was revealed that compounds **2i** and **3i** act as ATP non-competitive type-II inhibitor against CDK2 kinase enzymes and ATP competitive type-I inhibitor against EGFR kinase enzymes. However, in case of HER2, compounds **2i** act as ATP non-competitive type-II inhibitor and **3i** act as ATP competitive type-I inhibitor. Docking results of known inhibitors were compared with synthesised compounds and found synthesised **2i** and **3i** are superior than the known inhibitors in case of interactions. In addition, *in silico* drug likeness properties of quinazolin-4(3*H*)-one derivatives showed better predicted ADME values than lapatinib.

## 1. Introduction

**Figure 1.**
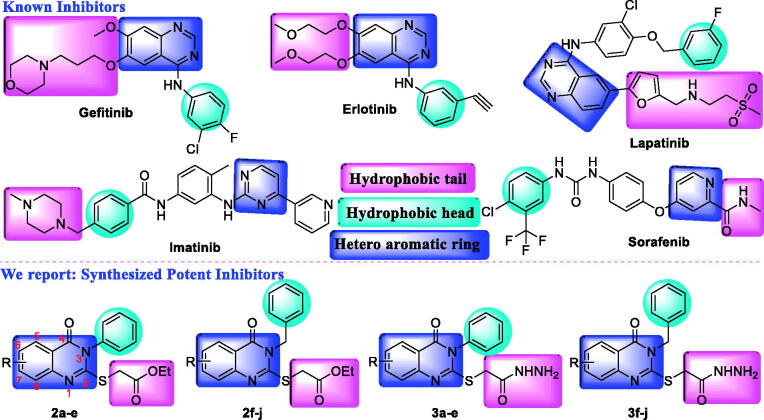
Reported tyrosine protein kinase inhibitors and potential quinazolin-4(3*H*)-one derivatives as tyrosine protein kinase inhibitory activity with their basic pharmacophoric features.

Cancer is a global health problem; in many countries, it is considered the second cause of morbidity and mortality^1^. Development of a new, safer and more efficient anticancer drugs with low cost and minimum side effects become an urgent need as the incidence of the cancer increase dramatically worldwide^2^. In the recent years, developing a new molecule-inhibiting protein kinase enzymes is a promising approach in developing anticancer agents^3^. Protein kinase enzymes are large groups of families that regulates cellular growth, division, proliferation, metastasis and survival. Protein kinases not only control cell division but also support the angiogenesis process that is required for tumour growth and metastasis. Activation of protein kinase enzymes through mechanisms, such as point mutations or over expression, could lead to large percentage of clinical cancers^4^. Quinazolinone derivatives are one of the promising *N*-containing heterocyclic compounds in drug researches area as they have a wide range of biological activities^5^, including antibacterial^6^^,^^7^, antioxidant^8^, anticonvulsant^9^^,^^10^, anti-inflammatory^11^^,^^12^, antitumor^13^^,^^14^, antiproliferative^15^^,^^16^, anticancer and many others^17^. Quinazolinone based molecules have been known as promising class of chemotherapeutic compounds as they shown a significant efficacy against different types of tumours^18^^,^^19^. Very recently, number of articles containing quinazolin-4(3*H*)-one moiety reported with urease^20–22^, phosphodiesterase^23^, aurora kinase^24^, PI3Kα^25^^,^^26^, HDAC^27^, USP7^28^ and VEGFR inhibitory^29^ activities as well as act as PPARγ and SUR agonists^30^. They are well known that quinazolinone based molecules such as gefitinib^31^^,^^32^, erlotinib^33^ and lapatinib^34^ are potent inhibitors of different tyrosine kinases^35^ (Figure 1). Epidermal Growth Factor Receptor (EGFR) belongs to the ErbB family of signalling proteins, which comprises four members (HER1, HER2, ErbB3 and ErbB4) are one of the most important tyrosine kinases involved in a cancer state which are over expressed in numerous of tumours such as breast, ovarian, prostate and lung^36^. Moreover, vascular endothelial growth factor (VEGFR) is transmembrane tyrosine kinase has a significant role in tumour angiogenesis, endothelial cell activation, proliferation and migration. It consists of three members (VEGFR1, VEGFR2 and VEGFR3). VEGFR2 is the major regulator of VEGF-driven responses in endothelial cells and can mediate proliferation, differentiation, and micro-vascular permeability^37^. However, more or less, almost all the tyrosine kinase inhibitors (TKIs) having side effects^38–44^. Therefore, there is an urgent need to develop the novel or modified anticancer drug molecules. It should be noted that, synthesised quinazolin-4(3*H*)-one derivative was shown to have no/negligible antimicrobial activity and therefore might show less adverse effect on future drug development^6^. Searching for a potent anticancer compound with no/less toxicity and at the same time having potent tyrosine protein kinase enzymes inhibitory activity on the other hand considering diverse biological activity of quinazolinone based molecules, here, we have designed and synthesised a series of quinazolin-4(3*H*)-one derivatives using reported methodology. In this series, considering the pharmacophoric features of various TKIs (Figure 1), we have modified the hydrophobic head and tail as well as quinazoline moiety of known TKIs by quinazolinone moiety. Moreover, a hydrophobic part of synthesised compounds was modified with halogens and methoxy group. Synthesised quinazolin-4(3*H*)-one derivatives were evaluated their cytotoxicity, studied structure–activity relationships (SARs), evaluated multiple tyrosine protein kinase enzymes (CDK2, EGFR1, HER2, and VEGFR2) inhibitory activity, analysed molecular docking and studied *in silico* drug likeness properties.

## 2. Results and discussion

### 2.2. Chemistry

**Scheme 1.**
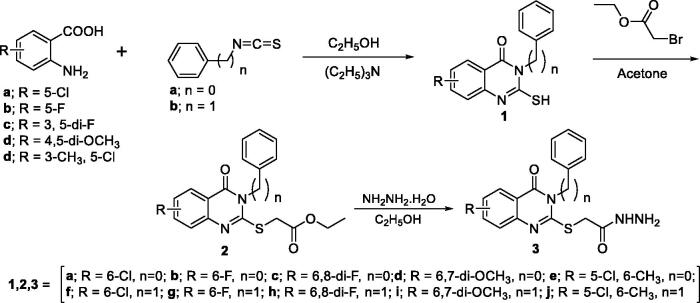
Synthesis of compounds **2** and **3**

Synthesis of quinazolin-4(3*H*)-one derivatives **1**, **2** and **3** was straightforward as depicted in Scheme 1 ^6^. In brief, substituted anthranilic acid (a-e) was refluxed with phenyl/benzyl isothiocyanate (a/b) in the presence of triethylamine in absolute ethanol at room temperature to obtain 2-mercapto-3-phenyl(or benzyl) quinazolin-4(3*H*)-one (**1a**-**j**) in 38–97% yields [45]. In the second step, compounds **1a**-**j** were alkylated with ethylbromoacetate in the presence of K_2_CO_3_ in boiling acetone to obtain quinazolin-4(3*H*)-one esters (**2a**-**j**) 48–97%. In the final step, the esters (**2a**-**j**) were stirred at room temperature with hydrazine hydrate in absolute ethanol to obtain final quinazolin-4(3*H*)-one hydrazide derivatives (**3a**-**j**) in excellent yields (73–96%) (Scheme 1). Structures of the synthesised quinazolin-4(3*H*)-one derivatives were confirmed using spectroscopic data (IR, ^1^H and ^13^CNMR and Mass). Physical properties were compared with literature reported values^6^^,^^9^.

### 2.2. Biological evaluation

#### 2.2.1. Cytotoxicity

The cytotoxicity of the synthesised quinazolin-4(3*H*)-one derivatives **2a–j** and **3a–j** was evaluated against two different cancer cell lines, namely MCF7 and A2780, and the results are summarised in Table 1. Among the quinazolin-4(3*H*)-one esters (**2a–j**) and quinazolin-4(3*H*)-one hydrazides (**3a**–**j**) series, the quinazolinone hydrazides (**3a**–**j**) exhibited the highest inhibitory activity against MCF7 cell lines than the quinazolin-4(3*H*)-one esters (**2a–j**). In brief, compounds **3a** and **3j** (Entry **3a** and **3j**; Table 1) exhibited IC_50_ at 0.20 ± 0.02 µM, and IC_50_ of all of them were in between 0.20 to 0.84 µM. On the other hand, compounds **2a–j** exhibited IC_50_ in between 0.73 to 3.79 µM except **2 b** (15.72 ± 0.07) and **2e (**14.88 ± 0.99). It should be noted that, positive control drug lapatinib shows IC_50_ at 5.90 ± 0.74 µM against MCF7 cell lines. In the case of A2780 cell lines, quinazolin-4(3*H*)-one hydrazides (**3a**–**j**) also exhibited the highest inhibitory activity than the quinazolin-4(3*H*)-one esters (**2a–j**). Among the quinazolin-4(3*H*)-one hydrazides (**3a**–**j**), all of them shows cytotoxicity in between 0.14 to 0.84 µM except **3a** (IC_50_ = 3.00 ± 1.20), whereas quinazolin-4(3*H*)-one esters (**2a–j**) shows cytotoxicity in between 0.49 to 2.98 µM except **2e** (16.43 ± 1.80). It also should be noted that, positive control drug lapatinib shows IC_50_ at 11.11 ± 1.03 µM against A2780 cell lines. In summary, all of the evaluated synthesised quinazolin-4(3*H*)-ones showed potent cytotoxicity towards MCF7 and A2780 cell lines (Table 1).

**Table 1.** Cytotoxicity of quinazolin-4(3*H*)-one **2a-j** and **3a-j** against MCF7 and A2780 cell lines

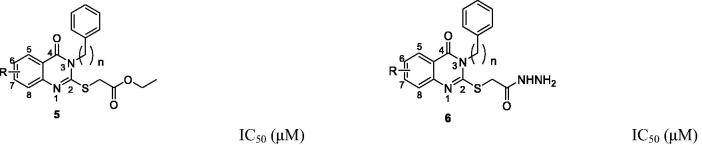									
No.	R	n	MCF7	A2780	No.	R	n	MCF7	A2780
**2a**	6-Cl	0	1.50 ± 0.01	2.05 ± 0.64	**3a**	6-Cl	0	0.20 ± 0.02	3.00 ± 1.20
**2b**	6-F		15.72 ± 0.07	2.98 ± 0.30	**3b**	6-F		1.32 ± 0.12	0.30 ± 0.06
**2c**	6,8-di-F		0.73 ± 0.18	0.49 ± 0.05	**3c**	6,8-di-F		0.33 ± 0.03	1.21 ± 0.01
**2d**	6,7-di-OCH_3_		1.56 ± 0.02	1.23 ± 0.10	**3d**	6,7-di-OCH_3_		0.50 ± 0.29	0.77 ± 0.66
**2e**	6-Cl,8-CH_3_		14.88 ± 0.99	16.43 ± 1.80	**3e**	6-Cl,8-CH_3_		0.74 ± 0.23	0.92 ± 0.11
**2f**	6-Cl	1	1.56 ± 0.24	1.32 ± 0.43	**3f**	6-Cl	1	0.34 ± 0.11	1.54 ± 0.60
**2g**	6-F		1.22 ± 0.17	2.40 ± 0.20	**3g**	6-F		0.22 ± 0.11	0.14 ± 0.03
**2h**	6,8-di-F		0.84 ± 0.16	1.40 ± 0.34	**3h**	6,8-di-F		0.84 ± 0.05	0.63 ± 0.16
**2i**	6,7-di-OCH_3_		2.70 ± 0.16	1.16 ± 0.10	**3i**	6,7-di-OCH_3_		0.56 ± 0.31	1.36 ± 0.07
**2j**	6-Cl,8-CH_3_		3.79 ± 0.96	2.68 ± 0.09	**3j**	6-Cl,8-CH_3_		0.20 ± 0.02	0.22 ± 0.03
**lapatinib**			5.90 ± 0.74	12.11 ± 1.03					

IC_50_ values are the mean ± SD of triplicate measurements; lapatinib was used as positive control.

#### 2.2.2. Structure–activity relationships (SARs) study of 2a–j and 3a–j

**Figure 2.**
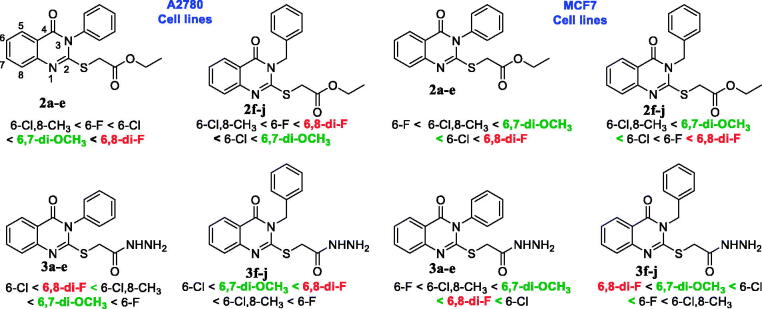
Structure–activity relationships (SARs) of quinazolin-4(3*H*)-ones **2a-j** and **3a-j**.

As all of the quinazolin-4(3*H*)-ones showed excellent cytotoxicity against both MCF7 and A2780 cell lines except compounds **2 b** and **2e,** the SARs study revealed no significant increase or decrease the cytotoxicity of quinazolin-4(3*H*)-ones **2a–j** and **3a–j**. However, still there are some minor relationships can be figure out. As depicted in Figure 2, rather considering *N*-phenyl/benzyl substitutions in quinazolinone ring and/or cell lines, compounds containing 6,8-di-fluoro substitutions (**2c** & **3c**) in benzene ring of quinazolin-4(3*H*)-one moiety of quinazolin-4(3*H*)-one esters (**2a-j**) shows increased the cytotoxicity. On the other hand, 6-fluoro derivatives (**3 b** and **3 g**) of quinazolin-4(3*H*)-one hydrazide (**3a-j**) was shown increased activity against A2780 cell lines. Besides those relationships, a conclusion can be made on Figure 2 that quinazolin-4(3*H*)-one containing dimethoxy substituents at 6 & 7 positions/fluoro substituents at 6 & 8 positions of benzene ring of quinazolin-4(3*H*)-one moiety of quinazolin-4(3*H*)-one esters (**2a-j**)/ quinazolin-4(3*H*)-one hydrazide (**3a-j**) having increased cytotoxicity.

### 2.2.3. Enzyme inhibitory activity of quinazolin-4(3H)-ones 2f-j and 3f-j

The promising cytotoxicity of quinazolin-4(3*H*)-ones **2a-j** and **3a-j**, especially our interested *N*-benzyl substituted quinazolin-4(3*H*)-ones **2f-j** and **3f-j**, which we were searching for potential protein kinases inhibitors, were studied for their inhibitory activities against several tyrosine protein kinases enzymes (CDK2, HER2, EGFR and VEGFR2). As summarised in Table 2, quinazolin-4(3*H*)-ones **2f-j** and **3f-j** exhibited good inhibitory activity against cyclin-dependent kinase 2 (CDK2)^46^, human epidermal growth factor receptor 2 (HER2), epidermal growth factor receptor (EGFR) and vascular endothelial growth factor receptor-2 (VEGFR2). Among the quinazolin-4(3*H*)-one evaluated, **2i** and **3i** exhibited strong enzyme inhibitory activity against CDK2 (IC_50_ = 0.173 ± 0.012 and 0.177 ± 0.032 µM, respectively), which is almost similar to the imatinib (IC_50_ = 0.131 ± 0.015 µM). Besides that, compound **2 h**, **3 g** and **3 h** also showed excellent enzyme inhibitory activity against CDK2 (Table 2; entry **2 h**, **3 g** and **3 h**). Rest of the quinazolin-4(3*H*)-ones also exhibited considerable enzyme inhibitory activity against CDK2. In case of HER2, **3i** showed excellent inhibitory activity (IC_50_ = 0.079 ± 0.015 µM) which is exactly similar with positive control lapatinib (IC_50_ = 0.078 ± 0.015 µM). Not only **3i** but also compounds **2 h**, **2i**, **3f** and **3 g** showed half fold activity as lapatinib, which are IC_50_ = 0.138 ± 0.012, 0.128 ± 0.024, 0.132 ± 0.014 and 0.112 ± 0.016 µM, respectively. As HER2, quinazolin-4(3*H*)-ones **2 h**, **2i**, **3 h** and **3i**, also showed excellent EGFR inhibitory activity comparing positive control erlotinib. The compounds showed IC_50_ = 0.102 ± 0.014, 0.097 ± 0.019, 0.128 ± 0.016 and 0.181 ± 0.011 µM, respectively, whereas, IC_50_ of erlotinib was 0.056 ± 0.012 µM. It should be noted that, all the quinazolin-4(3*H*)-ones tested was potent against EGFR kinase enzymes. Only VEGFR2 inhibitory activity was not satisfactory for all the compounds but still **2j**, **3 g** and **2i** was shown comparable activity against sorafenib. IC_50_ of the compounds **2j**, **3 g** and **3i** was 0.247 ± 0.015, 0.294 ± 0.011 and 0.257 ± 0.023 µM, respectively, whereas sorafenib shows at 0.091 ± 0.012 µM (Table 2).

**Table 2.** Inhibitory activities of quinazolin-4(3*H*)-ones **2f-j** and **3f-j** against CDK2, HER2, EGFR and VEGFR2 kinase

Entry	Kinase IC_50_ (µM)^a^				
	R	CDK2	HER2	EGFR	VEGFR2
**2f**	6-Cl	0.424 ± 0.012	0.264 ± 0.013	0.584 ± 0.011	1.915 ± 0.021
**2g**	6-F	0.444 ± 0.011	0.884 ± 0.016	0.232 ± 0.030	0.702 ± 0.013
**2h**	6,8-di-F	0.318 ± 0.023	0.138 ± 0.012	0.102 ± 0.014	2.191 ± 0.024
**2i**	6,7-di-OCH_3_	0.173 ± 0.012	0.128 ± 0.024	0.097 ± 0.019	2.846 ± 0.014
**2j**	6-Cl,8-CH_3_	0.862 ± 0.016	0.343 ± 0.015	0.813 ± 0.022	0.247 ± 0.015
**3f**	6-Cl	0.420 ± 0.013	0.132 ± 0.014	0.440 ± 0.015	0.651 ± 0.014
**3g**	6-F	0.207 ± 0.017	0.112 ± 0.016	0.221 ± 0.014	0.294 ± 0.011
**3h**	6,8-di-F	0.257 ± 0.016	0.248 ± 0.023	0.128 ± 0.016	1.372 ± 0.026
**3i**	6,7-di-OCH_3_	0.177 ± 0.032	0.079 ± 0.015	0.181 ± 0.011	0.257 ± 0.023
**3j**	6-Cl,8-CH_3_	0.473 ± 0.033	0.222 ± 0.012	0.371 ± 0.014	3.085 ± 0.016
Positive controls		0.131 ± 0.015^b^	0.078 ± 0.015^c^	0.056 ± 0.012^d^	0.091 ± 0.012^e^

^a^The values are the mean ± SD of triplicate measurements; ^b^imatinib; ^c^lapatinib; ^d^erlotinib; ^e^sorafenib.

### 2.3. In silico binding mechanism analysis

Considering the above experimental results of synthesised quinazolin-4(3*H*)-one derivatives **2a-j** and **3a-j**, which has been shown to have potent cytotoxicity against MCF7 and A2780 cell lines, and possessed excellent tyrosine kinases inhibitory activity, especially against CDK2, HER2 and EGFR, we further decided to analyse binding mechanism of those two quinazolin-4(3*H*)-one **2i** and **3i** with the respective kinases by molecular docking analysis. We therefore, performed the docking analysis of compounds **2i** and **3i** with EGFR, CDK2 and HER2 kinases as described in the method section.

#### 2.3.1. Docking analysis for quinazolin-4(3H)-one 2i and 3i with CDK2 kinase enzyme

**Figure 3.**
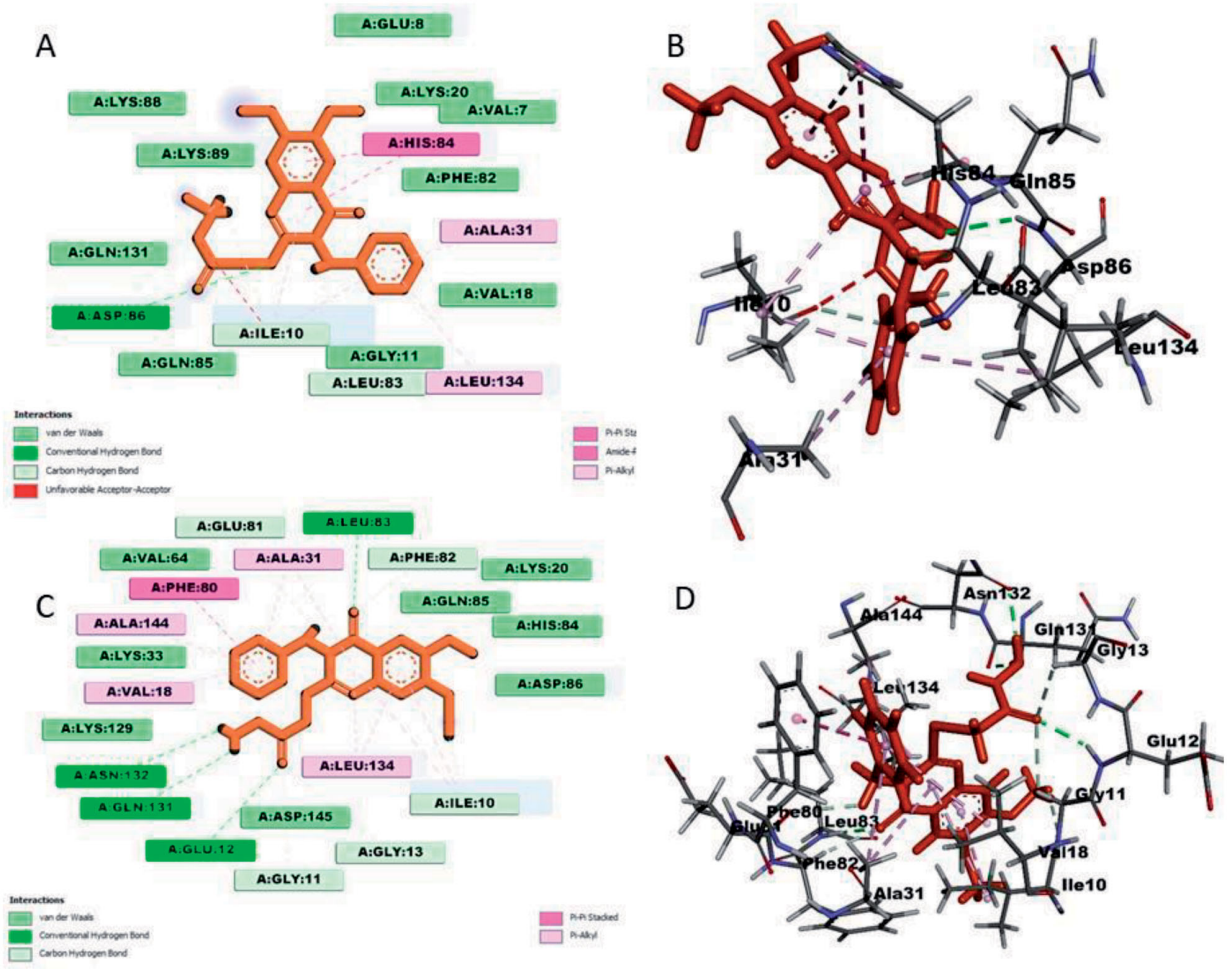
Docking analysis of quinazolin-4(3*H*)-one **2i** and **3i** with CDK2 protein kinase enzymes: (A) 2 D of **2i**; (B) 3 D of **2i**; (C) 2 D of **3i**; (D) 3 D of **2i**.

Quinazolin-4(3*H*)-one **2i** showed significant interactions with CDK2 kinase. It involved one conventional hydrogen bond interaction with Asp86, van der Waals interactions with Asp86, Ile10 and Gly11, pi-pi stacked bonding with His84 and several pi-alkyl interactions with Ala31, Ile10, Gly11 and Leu134 (Figure 3(A,B)). Compound **3i** comparatively showed significant interactions with CDK2 kinase than **2i**. Interactions involved four conventional hydrogen bonds interactions with Leu83, Glu12, Gln131 and Asn132. It also formed five van der Waals interactions with Ile10, Gly11, Gly13, Lys33, Phe82 and Glu81. Several pi-alkyl interactions were also observed for Ala31, Val18, Leu134, Ala144 and Ile10. Phe80 alone formed a single pi-pi stacked interaction (Figure 3(C,D)). From the recently published docking analysis of synthesised compounds with CDK2 kinase, it was found that active state kinase contains DFG motif which contains Asp145-Phe146- Gly147, whereas Leu83, Asp86and Asp145 form the ATP binding site of CDK2 through hydrogen bonds, where Asp145 belongs to the DFG motif. The phosphorylation of the C-terminal domain contains the catalytic residue (Glu51) required for the phosphorylation of Thr160 in the T-loop for its activation^46^.

For comparison study, reference imatinib was docked with CDK2 kinase (Figure S79 & S80 are inserted in the supporting information file) and found that, it is mainly interacted with CDK2 kinase domain via hydrogen bonds with Lys9, Ile10 and Leu83; pi-sigma interaction with Val18; pi-alkyl interactions with Ala31, Val64, Phe80 and Ala144; attractive charge interaction with Glu8. All these interactions were present for the studied compounds **2i** and **3i**. Thus, imatinib showed no superior interaction pattern than the studied compounds. Docking analysis of quinazolin-4(*3H*)-ones **2i** and **3i** lacked important interactions with DFG motif and ATP binding site residues thus both compounds might act as ATP non-competitive type II inhibitors.

#### 2.3.2. Docking analysis for quinazolin-4(3H)-one 2i and 3i with HER2 kinase enzyme

**Figure 4.**
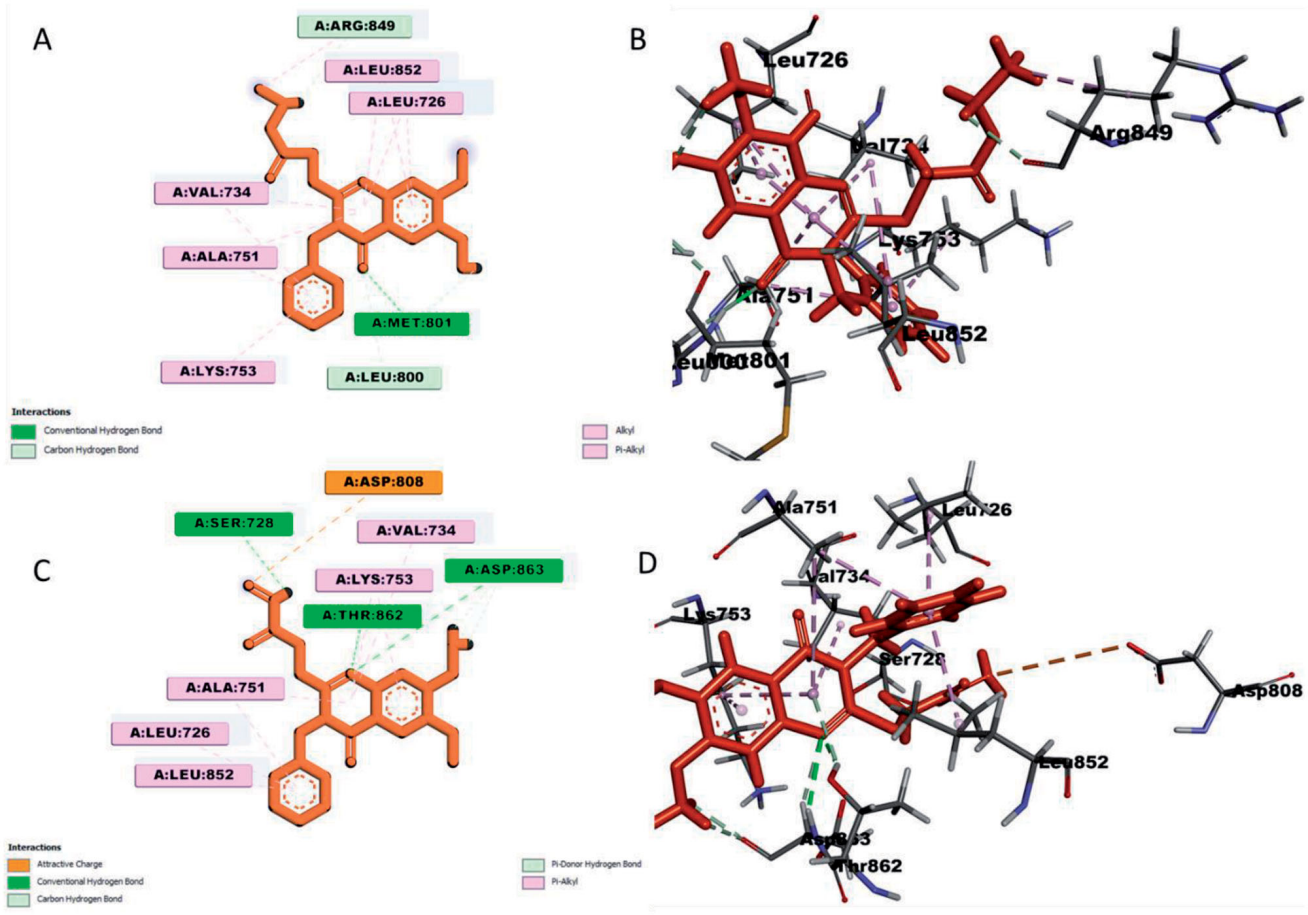
Docking analysis of quinazolin-4(3*H*)-one **2i** and **3i** with HER2 protein kinase enzyme: (A) 2 D of **2i**; (B) 3 D of **3i**; (C) 2 D of **2i**; (D) 3 D of **3i**.

Docking analysis revealed significant interactions of quinazolin-4(3*H*)-one **2i** with HER2 kinase enzyme. These interactions were mediated *via* hydrogen bond interactions with Met801 and Leu800, several alkyl and pi-alkyl interactions with Leu726, Leu852, Ala751, Val734, Lys753 and Arg849 (Figure 4(A,B)). Compound **3i** showed similar interactions with HER2 kinase as quinazolin-4(3*H*)-one **2i** except lacking pi-alkyl interaction with Arg849. It also formed additional three hydrogen bond interactions with Ser728, Thr862 and Asp863. Asp808 formed an attractive charge interaction (Figure 4(C,D)). From the published crystal structure of HER2 kinase enzyme, it was found that the DFG motif region ranges from Asp863–Gly865; the catalytic loop from Arg844–Asn850; and the activation loop from Asp863–Val884^47^.

Previous docking study of lapatinib with HER2^48^ showed that, its interacted with HER2 kinase via Ala751, Glu770 and Leu796 residues. In addition to that, two attractive or repulsive charged interactions were found between lapatinib and Gly727 and Asp808 residues. Comparing interaction of lapatinib and the studied compounds, it was found two similar interactions with Ala751 and Asp808. However, lapatinib lacked important interactions with active site residues such as Asp863. Therefore, the studied compounds **2i** and **3i** showed strong interaction than reference compound lapatinib. Docking analysis unveiled that quinazolin-4(3*H*)-one **2i** lacked important interactions with the DFG motif residues as well as catalytic loop residues which also does not involved the ATP binding site. Thus, compound **2i** can act as ATP non-competitive type-II inhibitor. On contrary, **3i** showed important interactions with DFG motif residue (Asp863) as well as activation loop residue thus can act as ATP competitive type I inhibitor.

#### 2.3.3. Docking analysis for quinazolin-4(3H)-one 2i and 3i with EGFR kinase enzyme

**Figure 5.**
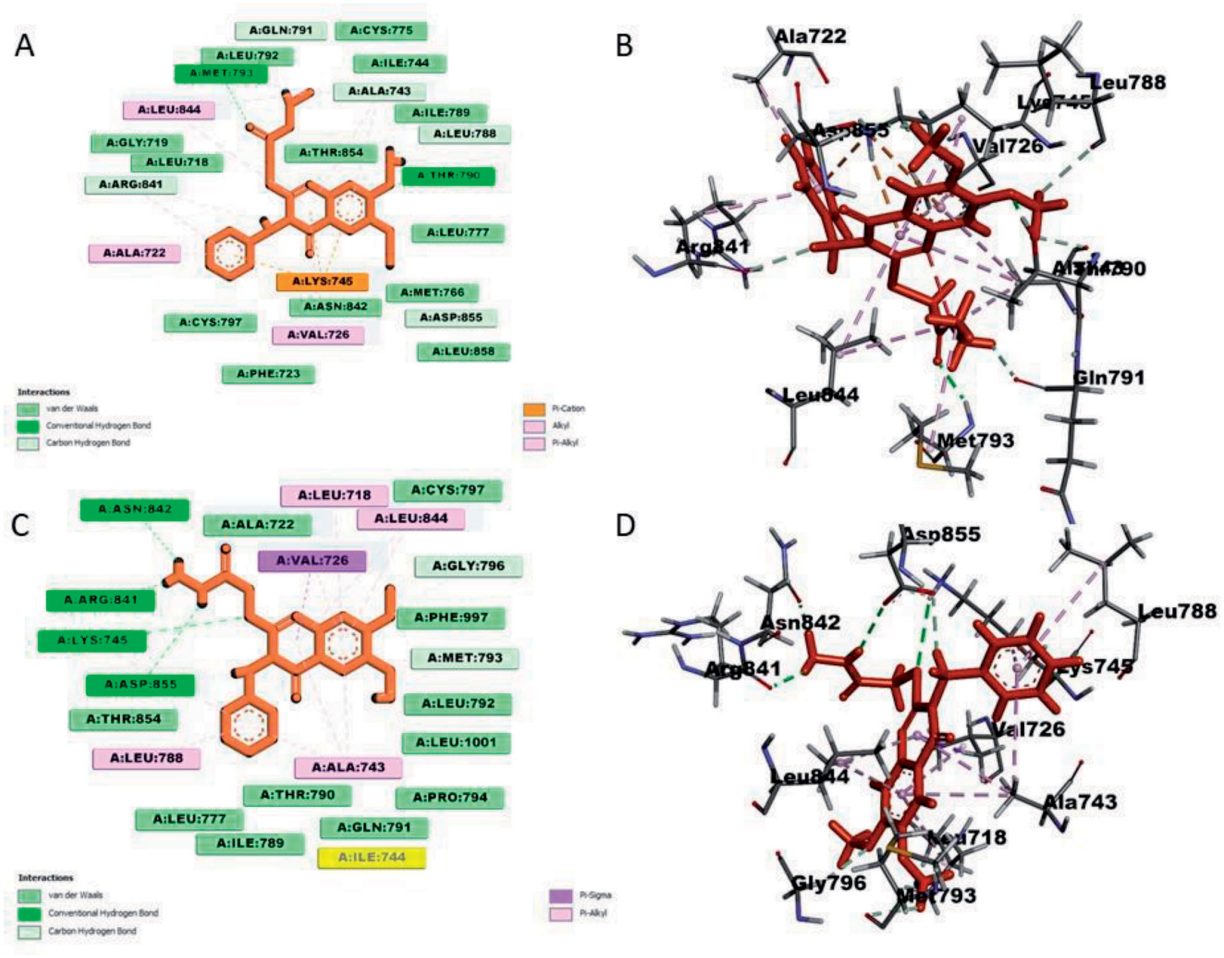
Docking analysis of quinazolin-4(3*H*)-one **2i** and **3i** with EGFR protein kinase enzyme: (A) 2 D of **2i**; (B) 3 D of **2i**; (C) 2 D of **3i**; (D) 3 D of **3i**.

Docking analysis of quinazolin-4(3*H*)-one **2i** with EGFR kinase involved two conventional hydrogen bonds with Met793 and Thr790, several van der Waals interactions with Gln791, Ala743, Leu788, Arg841 and Asp855. It also formed several alkyl and pi-alkyl interactions with Val726, Ala722, Arg841, Leu844, Ala743 and Met793. Lys745 made 3 pi–cation interactions with **2i** (Figure 5(A,B)). The other quinazolin-4(3*H*)-one **3i** formed four conventional hydrogen bonding with Arg841, Asn842, Lys745 and Asp855. Three van der Waals interactions were formed by Met793, Gly796 and Asp855. Moreover, several pi–alkyl interactions were observed for Leu788, Ala743, Lys745, Leu718 and Leu844. One pi–sigma interaction was formed by Val726 (Figure 5(C,D)). Inhibition of EGFR kinase mainly can be achieved by interactions with DGF motif residues and ATP binding site residues which contains Asp855 and Phe856^49^. From the docking analysis of quinazolin-4(3*H*)-one **2i** and **3i**, it was found that both quinazolin-4(3*H*)-one interacted with Asp855; thus, they can inhibit active EGFR kinase by interacting with DFG motif residue which also ATP-binding site of EGFR kinase. Comparing with established docking of erlotinib with EGFR kinase^50^, its lacked important DFG motif interactions and many important interactions with the active site residues even though it showed some interactions with Cys773, Met769 and Lys721. So, quinazolin-4(3*H*)-one **2i** and **3i** can act as ATP competitive type-I inhibitor against EGFR kinase with superiority to erlotinib.

### 2.4. In silico drug likeness property

Rational drug design is the most significant part in modern drug discovery approaches. In this regards, computational ADME (absorption, distribution, metabolism and excretion) analysis can help us to select the best drug in terms of cost, time and efficiency. Applying computational chemistry tools, *in vitro* and *in vivo* ADME prediction is now much more convenient, it can aid the pharmaceutical industries to screen thousands of compounds within a short time^51^. Here, synthesised quinazolin-4(3*H*)-one (**2** and **3**) were screened for predicted ADME values and the results are summarised in Table 3. Since high molecular weight compounds are always less effective in terms of intestinal absorption^52^^,^^53^, here, synthesised quinazolin-4(3*H*)-one derivatives’ (**2** and **3**) molecular weight were kept in between 344 − 414 Da. In case of hydrogen bond donor (HBD) (recommended value = ˂5) and hydrogen bond acceptor (HBA) values (recommended value = ˂10), compound **2a-j** and **3a-j** were superior than the lapatinib (HBD = 1; HBA = 8.25). Octanol/water partition coefficient of compounds **2a-j** and **3a-j** were in between 1.81 − 4.45, and solubility score −3.68 to −5.97, respectively, which are also in acceptable ranges and excellent than lapatinib^54^. Predicted logIC_50_ values for blockage of hERG K^+^ channels (loghERG) was found for compounds **2a-j** and **3a-j** in between −5.74 to −6.49 (lapatinib = −9.34) (recommended values is below −5^55^). Improved and well oral drug absorption and transdermal delivery efficiency for **2a-j** (Caco-2 range of 1142 − 1938) and **3a-j (**Caco-2 range of 168 − 386) were obtained which is better than the lapatinib (113)^56^. The blood–brain barrier (BBB) permeability [57] showed significant result for **2a-j** (−0.4 to −0.95) and **3a-j** (−0.8 to −1.76), whereas lapatinib gave −1.2. Madin–Darby canine kidney (MDCK) cell permeability^58^ values for **2a-j** gave much higher (740 − 3535), and **3a-j** gave in between range (93 − 489), whereas lapatinib gave 217. The synthesised quinazolin-4(3*H*)-one also gave a predicted human oral absorption rate of 100% for **2a-j** and 77 − 87% for **3a-j** (lapatinib 74%). Taken together, our designed quinazolin-4(3*H*)-one **2a-j** and **3a-j** showed higher predicted ADME values than lapatinib.

**Table 3.** Analysis of drug likeness and pharmacokinetic properties of quinazolin-4(3*H*)-ones **2a-j** and **3a-j** by QikProp

No.^a^	MW^b^	HBD^c^	HBA^d^	logPo/w^e^	logS^f^	logP	HERG^g^	Caco-2^h^	BBB^i^	MDCK^j^	HOA(%)^k^
**2a**	375	0	6	3.91	−5.66	9.2	−6.49	1142	−0.53	1773	100
**2b**	358	0	6	3.65	−5.28	9.2	−6.46	1146	−0.57	1307	100
**2c**	376	0	6	3.84	−5.52	9.02	−6.32	1148	−0.48	2078	100
**2d**	400	0	7.5	3.45	−4.69	9.62	−5.88	1267	−0.72	911	100
**2e**	389	0	6	4.14	−5.91	9.0	−6.37	1367	−0.50	1614	100
**2f**	389	0	6	4.27	−5.45	8.5	−6.31	1838	−0.40	2923	100
**2g**	372	0	6	4.00	−5.06	8.6	−6.28	1798	−0.46	2056	100
**2h**	390	0	6	4.24	−5.47	8.5	−6.20	1518	−0.41	3536	100
**2i**	414	0	7.5	3.87	−5.27	9.5	−6.40	1251	−0.95	740	100
**2j**	403	0	6	4.45	−5.97	8.5	−6.43	1468	−0.61	1627	100
**3a**	361	3	7	2.10	−4.60	15.0	−5.93	168	−1.32	217	79.1
**3b**	344	3	7	1.82	−4.17	15.0	−5.89	169	−1.35	160	77.45
**3c**	362	3	7	2.04	−4.50	14.8	−5.74	168	−1.26	253	78.77
**3d**	386	3	8.5	1.83	−3.68	15.1	−4.94	318	−1.17	215	82.45
**3e**	375	3	7	2.27	−4.05	14.2	−4.87	386	−0.80	489	86.56
**3f**	375	3	7	2.39	−4.60	14.6	−5.99	180	−1.44	221	81.27
**3g**	358	3	7	2.14	−4.25	14.6	−5.94	180	−1.47	162	79.83
**3h**	376	3	7	2.33	−4.48	14.4	−5.80	183	−1.38	260	81.05
**3i**	400	3	8.5	2.14	−4.36	15.3	−5.86	188	−1.76	93	80.19
**3j**	389	3	7	2.59	−4.77	14.3	−5.86	221	−1.40	204	84.04
**Lap^l^**	581	1	8.25	6.2	−8.5	13	−9.34	113	−1.2	217	74

^a^Compounds number; ^b^Molecular weight in Daltons (acceptable range: <500); ^c^Hydrogen bond donor (acceptable range: ≤5); ^d^Hydrogen bond acceptor (acceptable range: ≤10); ^e^Predicted octanol/water partition coefficient (acceptable range: −2 – 6.5); ^f^Predicted aqueous solubility, S in mol/dm^−3^ (acceptable range: −6.5 – 0.5); ^g^Predicted IC_50_ value for blockage of hERG K + channels (concern: below −5); ^h^Caco − 2 value, permeability to Caco-2 (human colorectal carcinoma) cells *in vitro*; ^I^Blood-brain barrier permeability (acceptable range: ∼ −0.4); ^j^Predicted apparent MDCK cell permeability in nm/sec, QPPMDCK= >500 is great, <25 is poor; ^k^Predicted human oral absorption on 0% to 100% scale (<25% is poor and >80% is high); ^l^Lab = lapatinib..

## 3. Experimental

### 3.1. Synthesis of quinazolin-4(3H)-ones

Quinazolin-4(3*H*)-ones were prepared using previously reported method^6^^,^^9^ and the structures of the synthesised quinazolin-4(3*H*)-ones were confirmed by various spectrometric analysis (NMR, IR, Mass), physical properties and comparing those reported values^6^^,^^9^^,^^45^^,^^59–61^.

#### General procedure for the synthesis of 1

A mixture of 5-choloanthranilic acid (1 mmol) and phenylisothiocynate (1 mmol) in absolute ethanol (15 ml), few drops of triethylamine was added. Reaction mixture was stirred at room temperature for 15 min followed by refluxed for 4 h. Precipitation was formed, filtered, dried and washed with petroleum ether. Pure compound **1a** was obtained from recrystallization.

6-Chloro-2-mercapto-3-phenylquinazolin-4(3*H*)-one (**1a**). White solid (38%). Mp. 261–3 °C^8^. ^1^H-NMR (600 MHz, DMSO-d_6_): δ 7.29 (*s*, 1H, ArH), 7.40–7.50 (*m*, 5H, ArH), 7.80–7.90 (*m*, 2H, ArH), 13.20 (brs, 1H, -SH) ppm. ^13 ^C-NMR (150 MHz, DMSO-d_6_): δ 118.01, 119.02, 118.52, 126.81, 128.75, 129.52, 136.00, 139.95, 139.62, 159.45, 176.62 ppm. MS (*m/z*): 289 [M(^35^Cl)+H]^+^, 291 [M(^37^Cl)+H]^+^.

6-Fluoro-2-mercapto-3-phenylquinazolin-4(3*H*)-one (**1 b**). White powder (77%). Mp. 317–318 °C (268–270 °C^27^). ^1^H-NMR (300 MHz, DMSO-d_6_): δ 7.31–7.27 (*m*, 2H), 7.53–7.42 (*m*, 4H), 7.75–7.65 (*m*, 2H) ppm. ^13 ^C-NMR (75 MHz, DMSO-d_6_): δ 112.53, 117.57, 118.39, 123.86, 128.22, 128.95, 136.50, 139.16, 157.03, 159.19, 159.44, 175.62 ppm. ESI-MS *m*/*z* 271.0 (M-H)^-^. MS (*m/z*): 273 [M + H]^+^; ESI-HRMS calcd. for C_14_H_8_FN_2_OS (M^-^)^-^ 271.0341, found 271.0345.

6,8-Difluoro-2-mercapto-3-phenylquinazolin-4(3*H*)-one (**1c**). White solid (73%). Mp. 290–93 °C^6^. IR (KBr, υ cm^−1^): 3323 (NH), 3130 (aromatic CH), 1689 (C = O), 1256, 1222, 1103; ^1^H-NMR (600 MHz, DMSO-d_6_): δ 7.25 (d, *J =* 7.2 Hz, 2H, ArH), 7.37–7.56 (*m*, 4H, ArH), 7.82 (*s*, 1H, ArH), 13,12 (*s*, 1H, -SH) ppm. ^13 ^C-NMR (150 MHz, DMSO-d_6_): δ 108.8, 109.04, 111.02, 111.22, 119.32, 128.65, 129.26, 129.44, 133.75, 138.72, 139.65, 144.75, 158.71, 176.52 ppm. MS (*m/z*): 290 [M(^18^F)+H]^+^, 291 [M(^19^F)+H]^+^.

6,7-Dimethoxy-2-mercapto-3-phenylquinazolin-4(3*H*)-one (**1d**). White powder (70%). Mp. 331 °C (316 °C^28^). ^1^H-NMR (300 MHz, DMSO-d_6_): δ 3.82 (*s*, 3H), 3.90 (*s*, 3H), 7.02 (*s*, 1H), 7.26–7.28 (*m*, 3H), 7.45–7.48 (*m*, 3H) ppm. ^13 ^C-NMR (75 MHz, DMSO-d_6_): δ 55.82, 56.01, 97.82, 106.95, 108.52, 128.11, 128.95, 129.04, 135.44, 139.47, 146.52, 155.32, 159.33 174.81 ppm. MS (*m/z*): 315 [M + H]^+^.

5-Chloro-2-mercapto-6-methyl-3-phenylquinazolin-4(3*H*)-one (**1e**). White solid (84%). Mp. 268–70 °C^6^. IR (KBr, υ cm^−1^): 3255 (NH), 3072 (aromatic CH), 2945 (aliphatic C-H), 1705 (C = O), 1255, 1211, 682 cm^−1^. ^1^H-NMR (600 MHz, DMSO-d_6_): δ 2.54 (*s*, 3H, -PhCH_3_), 7.25 (d, *J* = 6 Hz, 2H, ArH), 7.42 (d, *J* = 6 Hz, 1H, ArH), 7.48 (*s*, 2H, ArH), 7.7 (*s*, 1H, ArH), 7.74 (*s*, 1H, ArH), 12 (*s*, 1H, -SH) ppm. ^13 ^C-NMR (150 MHz, DMSO-d_6_): δ 17.71, 118.41, 124.44, 128.15, 128.36, 128.6,4 129.22, 129.44, 136.54, 137.62, 139.74, 159.48, 176.82 ppm. MS (*m/z*): 302 [M(^35^Cl)+H]^+^, 304 [M(^37^Cl)+H]^+^.

3-Benzyl-6-chloro-2-mercaptoquinazolin-4(3*H*)-one (**1f**). (KBr, υ cm^−1^): 1703 (C = O str.), 3167 (N-H str.) cm^−1^. ^1^H-NMR (600 MHz, DMSO-d_6_): δ, 5.65 (*s*, 2H, -CH_2_Ph), 7.20–7.35 (*m*, 5H, ArH), 7.40–7.50 (d, *J* = 8.5 Hz, 1H, ArH), 7.80 (dd, *J* = 9.0, 2.0 Hz, 1H, ArH), 7.90 (*s*,1H, ArH), 12.90 (brs, 1H, -SH) ppm. ^13 ^C-NMR (150 MHz, DMSO-d_6_): δ 17.61, 49.42, 117.32, 118.54, 126.85, 127.44, 128.74, 128.92, 136.01, 136.80, 138.55, 159.02, 176.81 ppm. MS (*m/z*): 303 [M(^35^Cl)+H]^+^, 305 [M(^37^Cl)+H]^+^.

3-Benzyl-6-fluoro-2-mercaptoquinazolin-4(3*H*)-one (**1 g**). White solid (59%). Mp. 209–11 °C ^6^. IR (KBr, υ cm^−1^): 3184 (NH), 3030 (aromatic CH), 2970 (aliphatic C-H), 1699 (C = O), 1298, 1234, 1165 cm^−1^. ^1^H-NMR (600 MHz, DMSO-d_6_): δ 5.64 (*s*, 2H, -CH_2_Ph), 7.24 (*m*, 5H, ArH), 7.46 (*s*, 1H, ArH), 7.67 (d, *J* = 8.4 Hz, 2H, Ar-H), 13.11 (s, 1H, -SH) ppm. ^13 ^C-NMR (150 MHz, DMSO-d_6_): δ 49.30, 112.81, 124.21, 124.44, 127.45, 127.55, 128.67, 133.62, 134.11, 136.55, 137.22, 138.24, 175.52 ppm. MS (*m/z*): 286 [M(^18^F)+H]^+^, 287 [M(^19^F)+H]^+^.

3-Benzyl-6,8-difluoro-2-mercaptoquinazolin-4(3*H*)-one (**1 h**). White solid (97%). Mp. 224–226 °C^6^. IR (KBr, υ cm^−1^): 3003 (aromatic C-H), 2968 (aliphatic C-H), 2600 (SH), 1716 (C = O), 1219, 1091 cm^−1^. ^1^H-NMR (600 MHz, DMSO-d_6_): δ 5.66 (*s*, 2H, -CH_2_Ph), 7.22 (*s*, 1H, ArH), 7.26–7.33 (*m*, 4H, ArH), 7.56 (d, *J* = 8.4, 1H, ArH), 7.82 (*s*, 1H, ArH), 13.15 (*s*, 1H, -SH) ppm. ^13 ^C-NMR (150 MHz, DMSO-d_6_): δ 49.41, 108.90, 111.15, 111.32, 127.45, 127.51, 128.65, 136.64, 154.54, 158.62, 160.64, 175.92 ppm. MS (*m/z*): 304 [M + H]^+^.

3-Benzyl-2-mercapto-6,7-dimethoxyquinazolin-4(3*H*)-one (**1i**). White solid (64%). Mp. 222–24 °C^8^. ^1^H-NMR (600 MHz, DMSO-d_6_): δ 3.80 (*s*, 6H, OCH3), 5.70 (*s*, 2H, -CH_2_Ph), 6.90 (*s*, 2H, ArH), 7.10–7.50 (*m*, 5H, ArH), 12.80 (s, 1H, -SH) ppm. ^13 ^C-NMR (150 MHz, DMSO-d_6_): δ 49.11, 56.32, 56.52, 98.35, 107.41, 108.52, 127.34, 127.71, 128.64, 135.66, 137.30, 147.32, 156.00, 159.31, 174.88 ppm. MS (*m/z*): 329 [M + H]^+^.

3-Benzyl-5-chloro-2-mercapto-6-methylquinazolin-4(3*H*)-one (**1j**). White solid (79%). Mp. 217–19 °C^6^. IR (KBr, υ cm^−1^): 3248 (NH), 3076 (aromatic CH), 2945 (aliphatic C-H), 1705 (C = O), 1246, 1228, 680 cm^−1^. ^1^H-NMR (600 MHz, DMSO-d_6_): δ 3.3 (*s*, 3H, -PhCH_3_), 5.67 (*s*, 2H, -CH_2_Ph), 7.21 (*s*, 1H, ArH), 7.28 (*s*, 4H, ArH), 7.69 (*s*, 1H, ArH), 7.76 (*s*, 1H, ArH), 11.9 (*s*, 1H, -SH) ppm. ^13 ^C-NMR (150 MHz, DMSO-d_6_): δ 17.62, 49.41, 117.51, 124.42, 127.45, 127.54, 128.62, 130.61, 136.65, 136.71, 159.12, 176.41 ppm. MS (*m/z*): 316 [M(^35^Cl)+H]^+^, 318 [M(^37^Cl)+H]^+^.

#### General procedure for the synthesis of 2

A mixture of substituted 2-mercapto-3-phenyl (or benzyl) quinazolin-4(3*H*)-one (10 mmol), ethyl bromoacetate (10 mmol) and anhydrous potassium carbonate (1.5 gm) in dried acetone were refluxed for 5–6 h. Solvent were evaporated under vacuum and the obtained residue was washed with water and recrystallized from the appropriate solvents obtained **2**.

Ethyl 2-((6-chloro-4-oxo-3-phenyl-3,4-dihydroquinazolin-2-yl)thio)acetate (**2a**). White solid (70%). Mp. 85–87 °C^8^. ^1^H-NMR (500 MHz, DMSO-d_6_): δ 1.22 (*t*, 3H, -CH_2_CH_3_), 3.99 (*s*, 2H, -SCH_2_-), 4.13 (*q*, 2H, -CH_2_CH_3_), 7.50 (*m*, 2H, ArH), 7.53 (d, *J* = 9.0 Hz, 1H, ArH), 7.61 (br, 3H, ArH), 7.88 (dd, *J* = 7.5, 2.5 Hz, 1H, ArH), 8.01 (d, *J* = 2.0 Hz, 1H, ArH) ppm. ^13 ^C-NMR (125 MHz, DMSO-d_6_): δ 14.12, 34.42, 61.06, 120.80, 125.51, 128.09, 129.24, 129.63, 130.11, 130.19, 135.05, 135.42, 145.70, 157.43, 159.60, 168.19 ppm. MS (*m/z*): 375 [M(^35^Cl)+H]^+^, 377 [M(^37^Cl)+H]^+^.

Ethyl 2-((6-fluoro-4-oxo-3-phenyl-3,4-dihydroquinazolin-2-yl)thio)acetate (**2 b**). White solid (87%). Mp. 124–127 °C (128 °C^29^). ^1^H-NMR (600 MHz, DMSO-d_6_): δ 1.22 (*t*, *J* = 7.0 Hz, 3H, CH_2_CH_3_), 3.97 (*s*, 2H, SCH_2_), 4.13 (*q*, *J* = 7.0 Hz, 2H, CH_2_CH_3_), 7.48–7.57 (*m*, 2H), 7.57–7.61 (*m*, 4H), 7.73–7.77 (*m*, 2H). ^13 ^C-NMR (150 MHz, DMSO-d_6_): δ 14.63, 34.88, 61.54, 111.87 (*J*_2_ = 20.2 Hz), 121.17, 123.94 (*J*_2_ = 20.2 Hz), 129.11, 129.16, 129.40, 129.80, 130.20, 130.66, 135.98, 144.45, 156.63, 160.45, 160.66 (*J*_F_ = 210 Hz), 168.76 ppm. MS (*m/z*): 359 [M + H]^+^

Ethyl 2-((6,8-difluoro-4-oxo-3-phenyl-3,4-dihydroquinazolin-2-yl)thio)acetate (**2c**). White cream (95%). Mp. 151–153 °C^6^. IR (KBr, υ cm^−1^): 3047 (aromatic C-H), 2972, 2924 (aliphatic C-H), 1739,1693 (C = O), 1105, 1020 (C-O) cm^−1^. ^1^H-NMR (600 MHz, DMSO-d_6_): δ 1.18 (*t*, *J* = 6.1 Hz, 3H, -CH_2_-CH_3_), 3.96 (*s*, 2H, -SCH_2_-), 4.09 (*q*, *J* = 6.1 Hz, 2H, -CH_2_CH_3_), 7.48–7.49 (*m*, 2H, Ar-H), 7.57–7.62 (*m*, 4H, Ar-H), 7.85 (ddd, *J* = 6.3, 2.2 Hz, 1H, Ar-H) ppm; ^13 ^C-NMR (150 MHz, DMSO-d_6_): δ 14.43, 34.97, 61.59, 107.81 (*J*_2_ = 20.1 Hz), 110.71 (*J*_2_ = 20.1, 19.8 Hz), 122.40, 129.67, 130.18, 130.79, 134.19 (*J*_3_ = 6.4 Hz), 135.84, 156.65 (*J*_F_ = 240 Hz), 157.70 (*J*_F_ = 210 Hz), 157.89, 159.65, 168.58 ppm. MS (*m/z*): 377 [M + H]^+^.

Ethyl 2-((6,7-dimethoxy-4-oxo-3-phenyl-3,4-dihydroquinazolin-2-yl)thio)acetate (**2d**). White solid (87%). Mp. 145–46 °C^8^. ^1^H-NMR (400 MHz, DMSO-d_6_): δ 1.23 (*t*, 3H, -CH_2_CH_3_), 3.85 (*s*, 3H, -OCH_3_), 3.92 (*s*, 3H, -OCH_3_), 3.97 (*s*, 2H, -SCH_2_-), 4.15 (*q*, 2H, -CH_2_CH_3_), 6.93 (*s*, 1H, ArH), 7.38 (*s*, 3H, ArH), 7.42–7.45 (*m*, 2H, ArH), 7.56–7.63 (*m*, 3H, ArH) ppm. ^13 ^C-NMR (100 MHz, DMSO-d_6_): δ 14.75, 34.86, 56.37, 56.52, 61.60, 106.27, 107.39, 112.70, 129.94, 130.14, 130.53, 136.45, 143.89, 148.66, 154.99, 155.55, 160.05, 168.97 ppm. MS (*m/z*): 401 [M + H]^+^.

Ethyl 2-((5-chloro-6-methyl-4-oxo-3-phenyl-3,4-dihydroquinazolin-2-yl)thio)acetate (**2e**). White solid (93%). Mp. 180–83 °C^6^. IR (KBr, υ cm^−1^): 3067 (aromatic C-H), 2985,2920 (aliphatic C-H), 1734,1683 (C = O), 1170, 1026 (C-O) cm^−1^. ^1^H-NMR (600 MHz, DMSO-d_6_): δ 1.17 (*t*, *J* = 6 Hz, 3H, -CH_2_CH_3_), 2.51 (*s*, 3H, -PhCH_3_), 3.98 (*s*, 2H, -SCH_2_-), 4.10 (*q*, *J =* 6.1 Hz, 2H, -OCH_2_CH_3_), 7.46 (d, *J* = 6.3 Hz, 2H, Ar-H), 7.59 (d, *J* = 5.7 Hz, 3H, Ar-H), 7.77 (*s*, 1H, Ar-H), 7.83 (*s*, 1H, Ar-H) ppm. ^13 ^C-NMR (150 MHz, DMSO-d_6_): δ 14.48, 16.80, 34.97, 61.60, 121.09, 123.45, 129.71, 130.07, 130.14, 130.67, 135.34, 136.00, 137.68, 144.82, 156.95, 160.39, 168.62 ppm. MS (*m/z*): 389 [M(^35^Cl)+H]^+^.

Ethyl 2-((3-benzyl-6-chloro-4-oxo-3,4-dihydroquinazolin-2-yl)thio)acetate (**2f**). White solid (90%). Mp. 73–75 °C^8^. IR (KBr, υ cm^−1^): 1684 (C = O str. of quinazoline-one ring), 1730 (C = O str. of ester) cm^−1^. ^1^H-NMR (300 MHz, DMSO-d_6_): δ 1.22 (*t*, 3H, -CH_2_CH_3_), 4.10 (*s*, 2H, SCH_2_), 4.14 (*q*, 2H, -CH_2_CH_3_), 5.34 (*s*, 2H, -CH_2_Ph), 7.29–7.30 (*m*, 3H, ArH), 7.34–7.36 (*m*, 2H, ArH), 7.84–7.88 (dd, *J* = 8.2, 2.0 Hz, 1H, ArH), 8.05 (d, *J* = 2.0 Hz, 1H, ArH) ppm. ^13 ^C-NMR (150 MHz, DMSO-d_6_): δ 14.10, 34.19, 47.18, 61.11, 119.78, 125.58, 126.80, 127.54, 128.04, 128.59, 130.23, 135.11, 135.17, 145.31, 156.99, 159.80, 168.07 ppm. MS (*m/z*): 389 [M(^35^Cl)+H]^+^, 391 [M(^37^Cl)+H]^+^.

Ethyl 2-((3-benzyl-6-fluoro-4-oxo-3,4-dihydroquinazolin-2-yl)thio)acetate (**2 g**). White solid (86%). Mp. 110–111 °C^6^. IR (KBr, υ cm^−1^): 3003 (aromatic C-H), 2968 (aliphatic C-H), 1734, 1716 (C = O), 1220, 1091(C-O) cm^−1^. ^1^H-NMR (600 MHz, DMSO-d_6_): δ 1.13 (*s*, 3H, -CH_2_-CH_3_), 4.04 (*s*, 2H, -SCH_2_-), 4.08 (*q*, 2H, -CH_2_CH_3_), 5.28 (*s*, 2H, -CH_2_Ph), 7.26–7.28 (*m*, 3H Ar-H), 7.26–7.28 (*m*, 2H Ar-H), 7.31–7.34 (*m*, 1H, Ar-H), 7.72 (dd, *J* = 7.5, 3.7 Hz, 1H ArH), 7.75 (*t*, *J* = 3.8 Hz 1H ArH) ppm. ^13 ^C-NMR (150 MHz, DMSO-d_6_): δ 14.52, 34.52, 47.52, 61.51, 111.57 (*J*_2_ = 20.2 Hz), 120.24, 123.87 (*J*_2_ = 20.6 Hz), 127.22, 128.01, 129.10, 129.43, 135.60, 144.03, 159.10 160.60 (*J*_F_ = 201 Hz), 160.73, 168.62 ppm. MS (*m/z*): 372 [M(^18^F)+H]^+^.

Ethyl 2-((3-benzyl-6,8-difluoro-4-oxo-3,4-dihydroquinazolin-2-yl)thio)acetate (**2 h**). White solid (97%). Mp. 120–123 °C^6^. IR (KBr, υ cm^−1^): 3003 (aromatic C-H), 2968, 2922 (aliphatic C-H), 1735, 1716 (C = O), 1217, 1091 (C-O) cm^−1^. ^1^H-NMR (600 MHz, DMSO-d_6_): δ 1.14 (*t*, *J* = 8.2 Hz, 3H, CH_2_-CH_3_), 4.06 (*s*, 2H, SCH_2_CO), 4.08 (*q*, 2H *J* = 6.0 Hz, OCH_2_CH_3_), 5.31 (*s*, 2H, CH_2_-Ph), 7.25–7.28 (*m*, 3H, Ar-H), 7.31–7.33 (*m*, 2H, Ar-H), 7.63 (dd, *J* = 6.6, 2.2 Hz, 1H, Ar-H), 7.81 (ddd, *J* = 8.6, 2.2 Hz, 1H, Ar-H) ppm. ^13 ^C-NMR (150 MHz, DMSO-d_6_): δ 14.43, 34.76, 47.89, 61.64, 107.81 (*J*_2_ = 20.1 Hz), 110.80 (*J*_2_ = 20.1, 19.8 Hz), 121.44 (*J*_3_ = 7.5 Hz), 128.09, 129.11, 133.91 (*J*_3_ = 6.3 Hz), 135.49, 156.25 (*J*_F_ = 240, 10.6 Hz), 157.38, 157.70 (*J*_C-F_ = 210, 10.1 Hz), 158.33, 159.76, 168.42 ppm. MS (*m/z*): 390 [M(^18^F)+H]^+^.

Ethyl 2-((3-benzyl-6,7-dimethoxy-4-oxo-3,4-dihydroquinazolin-2-yl)thio)acetate (**2i**). White solid (98%). Mp. 104–105 °C^8^. ^1^H-NMR (600 MHz, DMSO-d_6_): δ 1.21 (*t*, 3H, -CH_2_CH_3_), 3.83 (*s*, 2H, -SCH_2_-), 3.93 (*s*, 6H, OCH_3_), 4.15 (*q*, 2H, -CH_2_CH_3_), 5.32 (*s*, 2H, -CH_2_Ph), 6.99 (*s*, 2H, ArH), 7.22–7.35 (*m*, 3H, ArH), 7.41–7.51 (*m*, 2H, ArH). ^13 ^C-NMR (150 MHz, DMSO-d_6_): δ 14.42, 34.82, 47.02, 55.92, 55.97, 61.62, 105.11, 106.52, 111.32, 126.52, 127.11, 128.41, 135.62, 143.01, 153.54, 154.24, 154.56, 160.11, 166.20 ppm. MS (*m/z*): 415 [M + H]^+^.

Ethyl 2-((3-benzyl-5-chloro-6-methyl-4-oxo-3,4-dihydroquinazolin-2-yl)thio)acetate (**2j**). White solid (99%). Mp. 132–34 °C^6^. IR (KBr, υ cm^−1^): 3005 (aromatic C-H), 2918, 2848 (aliphatic C-H), 1714, 1647 (C = O), 1222, 1091 (C-O) cm^−1^. ^1^H-NMR (600 MHz, DMSO-d_6_): δ 1.14 (*t*, *J* = 6.1 Hz, 3H, -CH_2_-CH_3_), 2.53 (*s*, 3H, CH_3_-Ph), 4.07 (*s*, 2H, SCH_2_CO), 4.10 (*q*, *J* = 6.1 Hz, -2H, OCH_2_CH_3_), 5.32 (*s*, 2H, CH_2_-Ph), 7.26–7.28 (*m*, 3H, Ar-H), 7.32–7.34 (*m*, 2H, Ar-H), 7.74 (d, *J* = 1.0 Hz. 1H, Ar-H), 7.87 (d, *J* = 2.2 Hz. 1H, Ar-H) ppm. ^13 ^C-NMR (150 MHz, DMSO-d_6_): δ 14.44, 16.69, 34.73, 47.63, 61.66, 120.15, 123.49, 127.29, 128.04, 129.09, 130.20, 135.36, 135.69, 137.73, 144.49, 156.45, 160.56, 168.43 ppm. MS (*m/z*): 402 [M(^35^Cl)+H]^+^, 404 [M(^37^Cl)+H]^+^.

#### General procedure for the synthesis of 3

A mixture of compound **2** (10 mmol) and hydrazine hydrate (1–2 ml) in absolute ethanol was stirred in a sealed flask at room temperature for 1 to 3 days, reaction was monitored by TLC. The obtained solid was filtered, washed with water and recrystallized from ethanol to get the desired compound **3**.

2-((6-Chloro-4-oxo-3-phenyl-3,4-dihydroquinazolin-2-yl)thio)acetohydrazide (**3a**). White solid (81%). Mp. 181–83 °C^8^. ^1^H-NMR (300 MHz, DMSO-d_6_): δ 3.81 (*s*, 2H, -NHNH_2_), 4.36 (*s*, 2H, -SCH_2_-), 7.26 (*m*, 2H, ArH), 7.53 (*m*, 3H, ArH), 7.58 (*m*, 1H, ArH), 7.64 (*m*, 1H, ArH), 8.05 (*s*, 1H, ArH), 9.10 (*s*, 1H, -NHNH_2_) ppm. ^13 ^C-NMR (150 MHz, DMSO-d_6_): δ 33.78, 120.17, 125.49, 127.46, 128.43, 129.11, 129.63, 130.45, 134.31, 134.60, 145.33, 156.70, 159.71, 167.15 ppm. MS (*m/z*): 361 [M(^35^Cl)+H]^+^, 363 [M(^37^Cl)+H]^+^.

2-((6-Fluoro-4-oxo-3-phenyl-3,4-dihydroquinazolin-2-yl)thio)acetohydrazide (**3 b**). White solid (82%). Mp. 192–94 °C^29^. ^1^H-NMR (300 MHz, DMSO-d_6_): δ 3.80 (*s*, 2H, SCH_2_), 4.26 (*s*, 2H, NHNH_2_), 7.45 (dd, *J* = 6.6, 2.1 Hz. 2H, Ar-H), 7.55–7.596 (*m*, 3H), 7.68 (dd, *J* = 7.5, 3.7 Hz. 1H, Ar-H), 7.72–7.75 (Overlapped, 2H), 9.31 (*s*, 1H, NHNH_2_) ppm. ^13 ^C-NMR (150 MHz, DMSO-d_6_): δ 34.92, 111.67 (*J*_2_ = 19.9 Hz), 121.21 (*J*_3_ = 7.1 Hz), 123.74 (*J*_2_ = 20.7 Hz), 129.38 (*J*_3_ = 7.1 Hz), 129.83, 130.04, 130.57, 136.23, 144.59, 156.80, 159.91 (*J*_F_ = 198.4 Hz), 160.57, 166.62 ppm. MS (m/z): 344 [M(^18^F)+H]^+^.

2-((6,8-Difluoro-4-oxo-3-phenyl-3,4-dihydroquinazolin-2-yl)thio)acetohydrazide (**3c**). White solid (86%). Mp. 201–03 °C^6^. IR (KBr, υ cm^−1^): 3323, 3150 (N-H), 3041(aromatic C-H), 2978 (aliphatic C-H), 1687 (CO), 1637 (CO) cm^−1^. ^1^H-NMR (600 MHz, DMSO-d_6_): δ 3.86 (*s*, 2H, -SCH_2_-), 4.27 (*s*, 2H, -NHNH_2_), 7.49–7.51 (*m*, 2H, ArH), 7.60 (br, 2H, ArH), 7.65 (d, *J* = 6.5 Hz 1H), 7.89 (t, 1H), 9.29 (s, 1H, -NHNH_2_) ppm. ^13 ^C-NMR (150 MHz, DMSO-d_6_): δ 34.7, 120.1, 123.3, 127.2, 127.9, 129, 130, 135.2, 135.7, 138, 144.6, 156.7, 160.6, 166.3 ppm. MS (*m/z*): 362 [M(^18^F)+H]^+^.

2-((6,7-Dimethoxy-4-oxo-3-phenyl-3,4-dihydroquinazolin-2-yl)thio)acetohydrazide (**3d**). White solid (10%). Mp. 166–68 °C^8^. ^1^H-NMR (300 MHz, DMSO-d_6_): δ 3.84 (*s*, 2H, -SCH_2_-), 3.87 (*s*, 3H, -OCH_3_), 3.94 (*s*, 3H, -OCH_3_), 4.29 (*s*, 2H, -NHNH_2_), 7.11 (*s*, 1H, ArH), 7.39 (*s*, 1H, ArH), 7.44 (*m*, 2H, ArH), 7.57 (*m*, 3H, ArH), 9.37 (*s*, 1H, -NHNH_2_) ppm. ^13 ^C-NMR (150 MHz, DMSO-d_6_): δ 34.29, 55.75, 56.01, 105.64, 107.15, 112.17, 129.44, 129.83, 135.93, 143.46, 147.98, 154.61, 154.93, 160.10, 166.17 ppm. MS (*m/z*): 387 [M + H]^+^.

2-((5-Chloro-6-methyl-4-oxo-3-phenyl-3,4-dihydroquinazolin-2-yl)thio)acetohydrazide (**3e**). White solid (97%). Mp. 218–19 °C^6^. IR (KBr, υ cm^−1^): 3313–3265 (N-H), 3068 (aromatic C-H), 29819 (aliphatic C-H), 1685, 1654 (C = O) cm^−1^. ^1^H-NMR (600 MHz, DMSO-d_6_): δ 2.50 (*s*, 3H, -CH_3_), 3.82 (*s*, 2H, -SCH_2_-), 4.25 (br, 2H, -NHNH_2_), 7.45 (d, *J* = 5.5 Hz. 2H, ArH), 7.57 (br, 3H, ArH), 7.76 (*s*, 1H, ArH), 7.82 (*s*, 1H, ArH), 9.26 (*s*, 1H, -NHNH_2_) ppm. ^13 ^C-NMR (150 MHz, DMSO-d_6_): δ 17.26, 34.97, 121.06, 123.35, 129.76, 129.91, 130.06, 130.57, 135.24, 136.09, 137.94, 144.97, 157.16, 160.51, 166.52 ppm. MS (*m/z*): 374 [M(^35^Cl)+H]^+^, 376 [M(^37^Cl)+H]^+^.

2-((3-Benzyl-6-chloro-4-oxo-3,4-dihydroquinazolin-2-yl)thio)acetohydrazide (**3f**). White solid (89%). Mp. 151–52 °C^8^. IR (KBr, υ cm^−1^): 1638 (C = O str. of hydrazide), 1683 (C = O str. of quinazolinone ring), 3304–3330 (N-H str.) cm^−1^. ^1^H-NMR (400 MHz, DMSO-d_6_): δ 3.94 (*s*, 2H, SCH_2_), 4.31 (*s*, 2H, -NHNH_2_), 5.34 (*s*, 2H, -CH_2_Ph), 7.27–7.30 (*m*, 3H, ArH), 7.32–7.37 (*m*, 2H, ArH), 7.61 (d, *J* = 8.5 Hz, 1H, ArH), 7.87 (dd, *J* = 8.5, 2.5 Hz, 1H, ArH), 8.04 (d, *J* = 2.5 Hz, 1H, ArH), 9.38 (*s*, 1H, -NHNH_2_) ppm. ^13 ^C-NMR (100 MHz, DMSO-d_6_): δ 34.46, 47.66, 120.48, 126.07, 127.38, 128.08, 128.92, 129.17, 130.70, 135.55, 135.85, 146.04, 157.77, 160.53, 166.53 ppm. MS (*m/z*): 375 [M(^35^Cl)+H]^+^, 377 [M(^37^Cl)+H]^+^.

2-((3-Benzyl-6-fluoro-4-oxo-3,4-dihydroquinazolin-2-yl)thio)acetohydrazide (**3 g**). White solid (96%). Mp. 207–09 °C^6^. IR (KBr, υ cm^−1^): 3304, 3213 (N-H), 3037 (aromatic C-H), 2987 (aliphatic C-H), 1672 (CO), 1654 (CO) cm^−1^. ^1^H-NMR (600 MHz, DMSO-d_6_): δ 3.92 (*s*, 2H, SCH_2_CO), 4.30 (*s*, 2H, -NHNH_2_), 5.32 (*s*, 2H, CH_2_-Ph), 7.26–7.28 (*m*, 3H, ArH), 7.26–7.28 (*m*, 3H, ArH), 7.32–7.34 (*m*, 2H, ArH), 7.74–7.66 (*t*, *J* = 7.4, 3.8 Hz, 1H), 7.72–7.74 (*t*, *J* = 7.3 Hz, 1H), 7.80 (d, *J* = 7.0 Hz, 1H), 9.36 (*s*, 1H, NHNH_2_) ppm. ^13 ^C-NMR (150 MHz, DMSO-d_6_): δ 34.66, 47.51, 111.57 (*J*_2_ = 19.9 Hz), 120.24, 123.87 (*J*_2_ = 20.6 Hz), 127.28, 127.98, 129.10, 129.43, 135.86, 144.23, 156.7, 160.10 (*J*_F_ = 198.4 Hz), 160.82, 166.62 ppm. MS (*m/z*): 358 [M(^18^F)+H]^+^; 359 [M(^19^F)+H]^+^.

2-((3-Benzyl-6,8-difluoro-4-oxo-3,4-dihydroquinazolin-2-yl)thio)acetohydrazide (**3 h**). White solid (95%). Mp. 185–86 °C^6^. IR (KBr, υ cm^−1^): 3317, 3150(N-H), 3098 (aromatic C-H), 2989 (aliphatic C-H), 1695 (CO), 1635 (CO) cm^−1^. ^1^H-NMR (600 MHz, DMSO-d_6_): δ 3.95 (*s*, 2H, SCH_2_), 4.26 (s, 2H, -NHNH_2_), 5.34 (*s*, 2H, -CH_2_Ph), 7.27–7.28 (*m*, 3H, ArH), 7.32–7.35 (*m*, 2H, ArH), 7.66 (dd, *J* = 4.5, 2.3 Hz, 1H, ArH), 7.85 (dd, *J* = 7.8, 2.6 Hz, 1H, ArH), 9.32 (*s*, 1H, NHNH_2_) ppm. ^13 ^C-NMR (150 MHz, DMSO-d_6_): δ 34.84, 47.79, 107.76 (*J*_2_ = 20.4 Hz), 110.80 (*J*_2_ = 24.0 Hz), 121.47 (*J*_3_ = 9.2 Hz), 127.27, 128.03, 129.10, 134.04 (*J*_3_ = 10.7 Hz), 135.57, 156.35 (*J*_F_ = 220.0, 10.6 Hz), 157.68, 159.00 (*J*_C-F_ = 217.5, 8.2 Hz), 159.96, 166.21 ppm. MS (*m/z*): 376 [M(^18^F)+H]^+^

2-((3-Benzyl-6,7-dimethoxy-4-oxo-3,4-dihydroquinazolin-2-yl)thio)acetohydrazide (**3i**). White solid (98%). Mp. 104–05 °C^8^. ^1^H-NMR (300 MHz, DMSO-d_6_): δ 3.83 (*s*, 2H, -SCH_2_-), 3.93 (*s*, 3H, OCH_3_), 3.94 (*s*, 3H, OCH_3_), 4.33 (*s*, 2H, -NHNH_2_), 5.34 (*s*, 2H, -CH_2_Ph), 7.06 (*s*, 1H, ArH), 7.24–7.29 (*m*, 3H, ArH), 7.33–7.34 (*m*, 2H, ArH), 7.43 (*s*, 1H, ArH), 9.37 (*s*, 1H, NHNH_2_) ppm. ^13 ^C-NMR (150 MHz, DMSO-d_6_): δ 34.0, 46.65, 55.69, 55.96, 62.94, 105.52, 106.98, 111.48, 126.72, 127.36, 128.55, 135.79, 143.13, 148.11, 154.21, 154.95, 160.25, 166.10 ppm. MS (*m/z*): 401 [M + H]^+^

2-((3-Benzyl-5-chloro-6-methyl-4-oxo-3,4-dihydroquinazolin-2-yl)thio)acetohydrazide (**3j**). White solid (94%). Mp. 216–20 °C^6^. IR (KBr, υ cm^−1^): 3284, 3100 (N-H), 3060 (aromatic C-H), 2993 (aliphatic C-H), 1683, 1654 (C = O) cm^−1^. ^1^H-NMR (600 MHz, DMSO-d_6_): δ 2.50 (*s*, 3H, -CH_3_Ph), 3.95 (*s*, 2H, SCH_2_-), 4.30 (br, 2H, -NHNH_2_), 5.35 (*s*, 2H, -CH_2_Ph), 7.28–7.30 (*m*, 3H, ArH), 7.33–7.35 (*m*, 2H, ArH), 7.75 (dd, J = 2.0, 0.6 Hz, 1H, ArH), 7.88 (d, J = 2.2 Hz, 1H, ArH), 9.34 (*s*, 1H, -NHNH_2_) ppm. ^13 ^C-NMR (150 MHz, DMSO-d_6_): δ 17.17, 34.72, 47.58, 120.15, 123.40, 127.29, 127.98, 129.08, 130.04, 135.25, 135.79, 138.02, 144.63, 156.72, 160.69, 166.31 ppm. MS (*m*/*z*): 388 [M(^35^Cl)+H]^+^, 390 [M(^37^Cl)+H]^+^.

### 3.2. Cytotoxicity

The cytotoxicity of the synthesised quinazolin-4(3*H*)-ones was evaluated by MTT assay, as previously described^62–64^. Two cancer cell lines, MCF7 (human breast adenocarcinoma) and A2780 (human ovary adenocarcinoma), were used in this study, which were obtained from the ATCC (Rockville, MD, USA). They were subcultured in RPMI-1640 media (supplemented with 10% FBS and 1% antibiotics) at 37 °C and 5% CO_2_. Additionally, compounds were prepared at the same medium to obtain serial dilutions (50, 25, 19,1 and 0.1 µM). The two cell lines were separately cultured in 96-well plates (3 × 10^3^/well) and incubated at 37 °C overnight. The following day, before treating the cells with the compounds, each well of the T0 plate was treated with 50 μL MTT solution (2 mg/mL in phosphate buffered saline) and then incubated for 2–4 h. The media were aspirated, and the formazan crystals were solubilised by adding 150 µL DMSO. Absorbance was read on a multi-plate reader (BioRad) at 550 mm. Optical density of the purple formazan A550 was proportional to the number of viable cells. quinazolin-4(3*H*)-ones concentration causing 50% inhibition (IC_50_) compared to positive control cell growth (100%) was determined. The data were obtained from triplicates and analysed using statistical software.

### 3.3. In vitro cyclin-dependent kinase2 (CDK2) inhibitory activity

The CDK2 Assay Kit is designed to measure CDK2/CyclinA2 activity for screening and profiling applications, using Kinase-Glo® MAX as a detection reagent. The CDK2 Assay Kit comes in a convenient 96-well format, with enough purified recombinant CDK2/CyclinA2 enzyme, CDK substrate peptide, ATP and kinase assay buffer for 100 enzyme reactions^62–64^. The assay was performed according to the protocol supplied from the CDK2 Assay kit #79599. The CDK2/CyclinA2 activity at a single-dose concentration of 10 μM was performed, where the Kinase-Glo MAX luminescence kinase assay kit (Promega#V6071) was used. The compounds were diluted in 10% DMSO and 5 μl of the dilution was added to a 50 μl reaction so that the final concentration of DMSO was 1% in all of the reactions. All of the enzymatic reactions were conducted at 30 °C for 40 min. The 50 μl reaction mixture contained 40 mM Tris, pH 7.4, 10 mM MgCl_2_, 0.1 mg/mL BSA, 1 mM DTT, 10 mM ATP, Kinase substrate and the enzyme (CDK2/CyclinA2). After the enzymatic reaction, 50 μl of Kinase-Glo^®^ MAX Luminescence kinase assay solution was added to each reaction and the plates were incubated for 5 min at room temperature. Luminescence signal was measured using a Bio Tek Synergy 2 microplate reader.

### 3.4. In vitro human epidermal growth factor receptor 2 (HFR2)/epidermal growth factor receptor (EGFR) / vascular endothelial growth factor receptor 2 (VEGFR2) inhibitory activity

The HER2/EGFR/VEGFR2 assay kit is designed to measure HER2/EGFR/VEGFR-2 inhibitory activity for screening and profiling applications, using Kinase-Glo^®^ MAX as a detection reagent. The HER2/EGFR/VEGFR2 assay kit comes in a convenient 96-well format, with enough purified recombinant HER2/EGFR/VEGFR2 enzyme, HER2/EGFR/VEGFR2 substrate, ATP and kinase buffer 1 for 100 enzyme reactions. The assay was performed according to the protocol supplied from the HER2/EGFR/VEGFR2 kinase assay kit #40721, #40321 and #40325, respectively^65^^,^^66^. The HER2/EGFR/VEGFR2 activity at a single-dose concentration of 10 μM was performed, where the Kinase-Glo MAX luminescence kinase assay kit (Promega#V6071) was used. The quinazolin-4(3*H*)-ones were diluted in 10% DMSO and 5 μl of the dilution was added to a 50 μl reaction so that the final concentration of DMSO was 1% in all of the reactions. All of the enzymatic reactions were conducted at 30 °C for 40 min. The 50 μl reaction mixture contained 40 mM Tris, pH 7.4, 10 mM MgCl_2_, 0.1 mg/mL BSA, 1 mM DTT, 10 mM ATP, kinase substrate and the enzyme HER2 / EGFR / VEGFR2. After the enzymatic reaction, 50 μl of Kinase-Glo^®^ MAX luminescence kinase assay solution was added to each reaction and the plates were incubated for 5 min at room temperature. Luminescence signal was measured using a Bio Tek Synergy 2 microplate reader (For detail enzyme assay protocol and the results, please see supplementary data).

### 3.5. Molecular docking and in silico ADME analysis

In order to perform molecular docking analysis, the protein data bank (PDB) structures of CDK2, HER2 and EGFR, kinase domains were downloaded from Research Collaboratory for Structural Bioinformatics (RCSB) PDB database (https://www.rcsb.org/) in PDB format. The PDB ID used for CDK2 kinase domain, HER2 kinase domain and EGFR kinase domain were 2BHE, 3PP0 and 3POZ, respectively^47^^,^^67^. Proteins and quinazolin-4(3*H*)-one were prepared for docking by using an established procedure^62–64^. Protein and compounds structures were energy minimised, refined and prepared for docking study by Schrödinger Maestro (Version 2018–4). OLPS3 force field and extra precision (XP) docking protocol was selected to generate induced fit docking scores which was explained in the established procedure^21^. Van der Waals scaling factor and partial charges cut-off were selected to be 0.85 and 0.15, respectively, for ligand molecules. The docking cut-off value was fixed at −10.00 kcal/mole for the screening of best poses of the docked compounds for subsequent processing. Discovery Studio was used for making 2 D interaction figures. Pymol was used to generate the 3 D and surface representation figures. For the *in silico* ADME analysis, all the quinazolin-4(3*H*)-one’ structures were prepared with the LigPrep module of Schrodinger Maestro and ADME was calculated by the Qikprop module of the same software package^62–64^.

## 4. Conclusion

Quinazolin-4(3*H*)-one derivatives exhibited excellent inhibitory activity against MCF-7 and A2780 cell lines and comparing known lapatinib. Molecular mechanism of action of quinazolin-4(3*H*)-one derivatives revealed that two quinazolin-4(3*H*)-ones (**2i** and **3i**) are the potent tyrosine kinases inhibitors against CDK2, HER2, EGFR tyrosine protein kinases enzymes. Two quinazolin-4(3*H*)-ones **2i** and **3i** act as ATP non-competitive type II inhibitor against CDK2 kinase and ATP competitive type-I inhibitor against EGFR kinase enzymes. On the other hand, **2i** act as ATP non-competitive type-II inhibitor and **3i** as ATP competitive type-I inhibitor against HER2 kinase enzyme. Comparison of docking results of reference and synthesised compounds exhibited that synthesised **2i** and **3i** are much more superior than reference compounds. *In silico* drug likeness properties of quinazolin-4(3*H*)-ones exhibited better predicted ADME values than lapatinib.

## Supplementary Material

Supplemental Material
