# Supplementary material for: Quinazolin-4(3H)-one based potential multiple tyrosine kinase inhibitors with excellent cytotoxicity
Source: J Enzyme Inhib Med Chem. 2021 Sep 22;36(1):2055–67. doi: 10.1080/14756366.2021.1972992 (PMC8462848; doi:10.1080/14756366.2021.1972992)

Quinazolin-4(3H)-one based Potential Multiple Tyrosine Kinase Inhibitors with Excellent Cytotoxicity

Tebyan O. Mirgany[a], Ashraf N. Abdalla[b], Md Arifuzzaman[c], A. F. M. Motiur Rahman[a,*] and Huda S. Al-Salem[a,*]

[a]Department of Pharmaceutical Chemistry, College of Pharmacy, King Saud University, 11421 Riyadh, Saudi Arabia

[b]Department of Pharmacology and Toxicology, Faculty of Pharmacy, Umm Al-Qura University, 21922 Makkah, Saudi Arabia

[c]College of Pharmacy, Yeungnam University, 38241 Gyeongsan, Korea

***** Corresponding Authors: hhalsalem@ksu.edu.sa (H.S.A.-S.); afmrahman@ksu.edu.sa (A.F.M.M.R.); Tel.: +933-11-29-22740 (H.S.A-S.); Tel.: +933-11-43-70237 (A.F.M.M.R.)

Entry	Contents	Page no.	Entry	Contents	Page no.
Table S1	Data of enzyme assays for quinazolin-4(*3H*)-ones **2f-j** and **3f-j**	5-8	Figure S14	^{1}HNMR of compound **2d**	22
Figure S1	Calculation of the IC_{20} Values of Protein Kinase Enzyme CDK2 for **2f**	9	Figure S15	^{1}HNMR of compound **2d (**extended**)**	23
Figure S2	^{1}HNMR of compound **2a**	10	Figure S16	^{1}HNMR of compound **2d (**extended**)**	24
Figure S3	^{1}HNMR of compound **2a (**extended**)**	11	Figure S17	^{13}CNMR of compound **2d**	25
Figure S4	^{1}HNMR of compound **2a (**extended**)**	12	Figure S18	^{13}CNMR of compound **2d** (extended)	26
Figure S5	^{13}CNMR of compound **2a**	13	Figure S19	^{13}CNMR of compound **2d** (extended)	27
Figure S6	^{13}CNMR of compound **2a** (extended)	14	Figure S20	^{1}HNMR of compound **2e**	28
Figure S7	^{1}HNMR of compound **2b**	15	Figure S21	^{1}HNMR of compound **2e (**extended**)**	29
Figure S8	^{13}CNMR of compound **2b**	16	Figure S22	^{13}CNMR of compound **2e**	30
Figure S9	^{13}CNMR of compound **2b** (extended)	17	Figure S23	^{13}CNMR of compound **2e** (extended)	31
Figure S10	^{1}HNMR of compound **2c**	18	Figure S24	^{1}HNMR of compound **2f**	32
Figure S11	^{1}HNMR of compound **2c (**extended**)**	19	Figure S25	^{1}HNMR of compound **2f (**extended**)**	33
Figure S12	^{13}CNMR of compound **2c**	20	Figure S26	^{1}HNMR of compound **2f (**extended**)**	34
Figure S13	^{13}CNMR of compound **2c** (extended)	21	Figure S27	^{13}CNMR of compound **2f**	35

Entry	Contents	Page no.	Entry	Contents	Page no.
Figure S28	^{13}CNMR of compound **2f** (extended)	36	Figure S42	^{1}HNMR of compound **3b**	50
Figure S29	^{13}CNMR of compound **2f** (extended)	37	Figure S43	^{1}HNMR of compound **3b** (extended)	51
Figure S30	^{1}HNMR of compound **2h**	38	Figure S44	^{13}CNMR of compound **3b**	52
Figure S31	^{1}HNMR of compound **2h** (extended)	39	Figure S45	^{13}CNMR of compound **3b** (extended)	53
Figure S32	^{13}CNMR of compound **2h**	40	Figure S46	^{1}HNMR of compound **3c**	54
Figure S33	^{13}CNMR of compound **2h** (extended)	41	Figure S47	^{1}HNMR of compound **3c** (extended)	55
Figure S34	^{1}HNMR of compound **2j**	42	Figure S48	^{1}HNMR of compound **3d**	56
Figure S35	^{1}HNMR of compound **2j** (extended)	43	Figure S49	^{1}HNMR of compound **3d** (extended)	57
Figure S36	^{13}CNMR of compound **2j**	44	Figure S50	^{1}HNMR of compound **3d** (extended)	58
Figure S37	^{13}CNMR of compound **2j** (extended)	45	Figure S51	^{13}CNMR of compound **3d**	59
Figure S38	^{1}HNMR of compound **3a**	46	Figure S52	^{13}CNMR of compound **3d** (extended)	60
Figure S39	^{1}HNMR of compound **3a** (extended)	47	Figure S53	^{13}CNMR of compound **3d** (extended)	61
Figure S40	^{13}CNMR of compound **3a**	48	Figure S54	^{1}HNMR of compound **3e**	62
Figure S41	^{13}CNMR of compound **3a** (extended)	49	Figure S55	^{1}HNMR of compound **3e** (extended)	63

Entry	Contents	Page no.	Entry	Contents	Page no.
Figure S56	^{13}CNMR of compound **3e**	64	Figure S70	^{1}HNMR of compound **3i**	78
Figure S57	^{13}CNMR of compound **3e** (extended)	65	Figure S71	^{1}HNMR of compound **3i (**extended**)**	79
Figure S58	^{1}HNMR of compound **3f**	66	Figure S72	^{13}CNMR of compound **3i**	80
Figure S59	^{1}HNMR of compound **3f (**extended**)**	67	Figure S73	^{13}CNMR of compound **3i** (extended)	81
Figure S60	^{1}HNMR of compound **3f (**extended**)**	68	Figure S74	^{13}CNMR of compound **3i** (extended)	82
Figure S61	^{13}CNMR of compound **3f**	69	Figure S75	^{1}HNMR of compound **3j**	83
Figure S62	^{13}CNMR of compound **3f** (extended)	70	Figure S76	^{1}HNMR of compound **3j (**extended**)**	84
Figure S63	^{13}CNMR of compound **3f** (extended)	71	Figure S77	^{13}CNMR of compound **3j**	85
Figure S64	^{1}HNMR of compound **3g**	72	Figure S78	^{13}CNMR of compound **3j** (extended)	86
Figure S65	^{1}HNMR of compound **3g (**extended**)**	73	Figure S79	Docking analysis of Imatinib with CDK2 protein kinase enzymes (2D)	87
Figure S66	^{1}HNMR of compound **3h**	74	Figure S80	Docking analysis of Imatinib with CDK2 protein kinase enzymes (3D)	88
Figure S67	^{1}HNMR of compound **3h (**extended**)**	75			
Figure S68	^{13}CNMR of compound **3h**	76			
Figure S69	^{13}CNMR of compound **3h** (extended)	77			

Table S1. Data of enzyme assays for quinazolin-4(*3H*)-ones **2f-j** and **3f-j**

Entry	Conc. (µM)	Log Conc	% Inhibition*			
			CDK2[a]	HER2[b]	EGFR[c]	VEGFR-2[d]
**2f**	10	4	83.98922	89.7829	83.22733	38.31214
	1	3	32.93412	74.78317	29.20912	40.07811
	0.1	2	31.2203	38.87342	19.13993	20.22937
	0.01	1	4.322933	2.332072	2.182347	3.49292
	0	0	0	0	0	0
**2g**	10	4	91.40237	94.41883	91.22333	29.22188
	1	3	78.23093	83.47739	80.11322	44.11811
	0.1	2	20.72383	44.2223	48.919	18.83473
	0.01	1	8.377334	17.33424	22.87472	2.432422
	0	0	0	0	0	0
**2h**	10	4	80.22473	82.34772	77.28212	87.99939
	1	3	24.41772	32.32294	21.00403	70.79978
	0.1	2	12.47312	38.87997	27.49311	38.87422
	0.01	1	2.433132	9.072877	3.707978	13.33288
	0	0	0	0	0	0

Continue

Entry	Conc. (µM)	Log Conc	% Inhibition*			
			CDK2[a]	HER2[b]	EGFR[c]	VEGFR-2[d]
**2i**	10	4	83.72223	94.80949	82.33233	77.83293
	1	3	37.84212	83.71031	39.14992	20.3214
	0.1	2	40.49277	24.393	43.72023	31.82722
	0.01	1	4.281938	20.37133	11.77113	8.129233
	0	0	0	0	0	0
**2j**	10	4	83.72223	93.47413	90.24999	33.87023
	1	3	37.84212	82.38943	84.32823	38.89939
	0.1	2	40.49277	47.03143	49.87498	20.32732
	0.01	1	4.281938	12.3242	22.24732	0.324072
	0	0	0	0	0	0
**3f**	10	4	81.0179	90.2318	84.84279	79.32414
	1	3	33.28243	82.23039	31.39133	23.41347
	0.1	2	34.70842	47.82378	31.32724	29.80742
	0.01	1	2.229427	17.2713	3.307094	3.712741
	0	0	0	0	0	0

Continue

Entry	Conc. (µM)	Log Conc	% Inhibition*			
			CDK2[a]	HER2[b]	EGFR[c]	VEGFR-2[d]
3g	10	4	89.0487	94.82233	94.00319	90.30374
	1	3	74.07129	83.8043	81.27912	71.90292
	0.1	2	47.0792	23.42332	40.30789	40.89493
	0.01	1	14.94703	23.38303	11.70979	3.727423
	0	0	0	0	0	0
3h	10	4	82.11073	89.78738	87.24379	33.09233
	1	3	70.2323	77.32094	29.21273	33.42122
	0.1	2	19.91212	44.3982	38.01248	19.47413
	0.01	1	3.741398	2.734039	8.397923	3.172342
	0	0	0	0	0	0
3i	10	4	90.37338	94.84083	92.44329	89.43343
	1	3	72.07978	84.3382	77.20833	37.2347
	0.1	2	44.30812	20.3282	41.22191	33.49032
	0.01	1	10.2842	17.88828	3.377791	10.18993
	0	0	0	0	0	0

Continue

Entry	Conc. (µM)	Log Conc	% Inhibition*			
			CDK2[a]	HER2[b]	EGFR[c]	VEGFR-2[d]
**3j**	10	4	83.98308	92.21808	89.90274	74.38022
	1	3	38.38783	72.04332	81.01149	41.13224
	0.1	2	42.84933	39.4822	48.83702	22.30779
	0.01	1	8.129147	2.332031	18.72822	1.983174
	0	0	0	0	0	0
**Control Drugs**	10	4	92.32944	92.24928	93.94249	91.37209
	1	3	77.22823	84.3391	83.30032	74.77711
	0.1	2	24.0709	23.23323	30.22733	24.42391
	0.01	1	14.94703	23.32399	28.48922	23.19801
	0	0	0	0	0	0

*Here, only one experimental data is given, and average of triplicated data are provided in the table 1. [a]Imatinib, [b]Lapatinib, [c]Erlotinib, [d]Sorafenib were used as control for protein kinase enzymes CDK2, HER2, EGFR and VEGFR2 inhibitory evaluation, respectively.

Figure S1. Calculation of the IC_{20} Values of Protein Kinase Enzyme CDK2 for **2f**

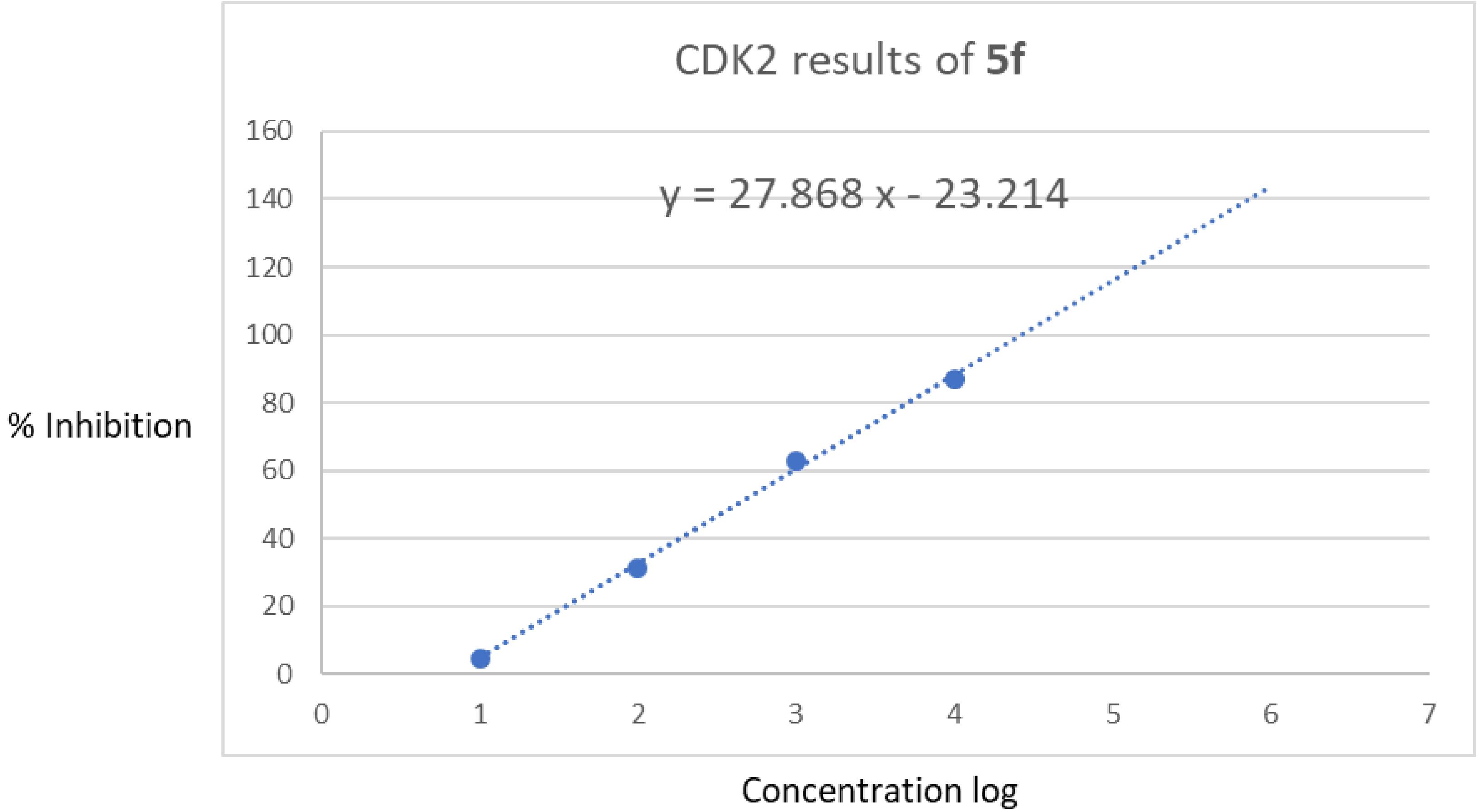

$y = 27.838\ x - 23.214$

$20 = 27.838\ x - 23.214$

$20 + 23.214 = 27.838\ x$

$73.214 = 27.838\ x$

$x = 73.214 / 27.838$

$x = 2.3272$

So, IC_{20} = 2.3272 (conc log) = 0.4238 µM

* All the kinase IC_{20} (µM) were calculated according to the above equations.

Figure S2. ^{1}HNMR of compound **2a**

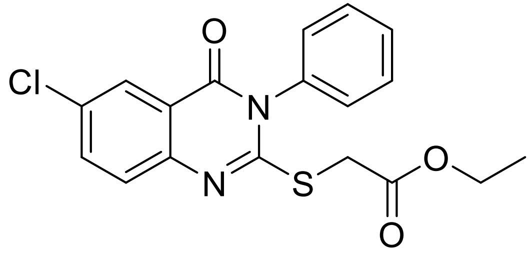

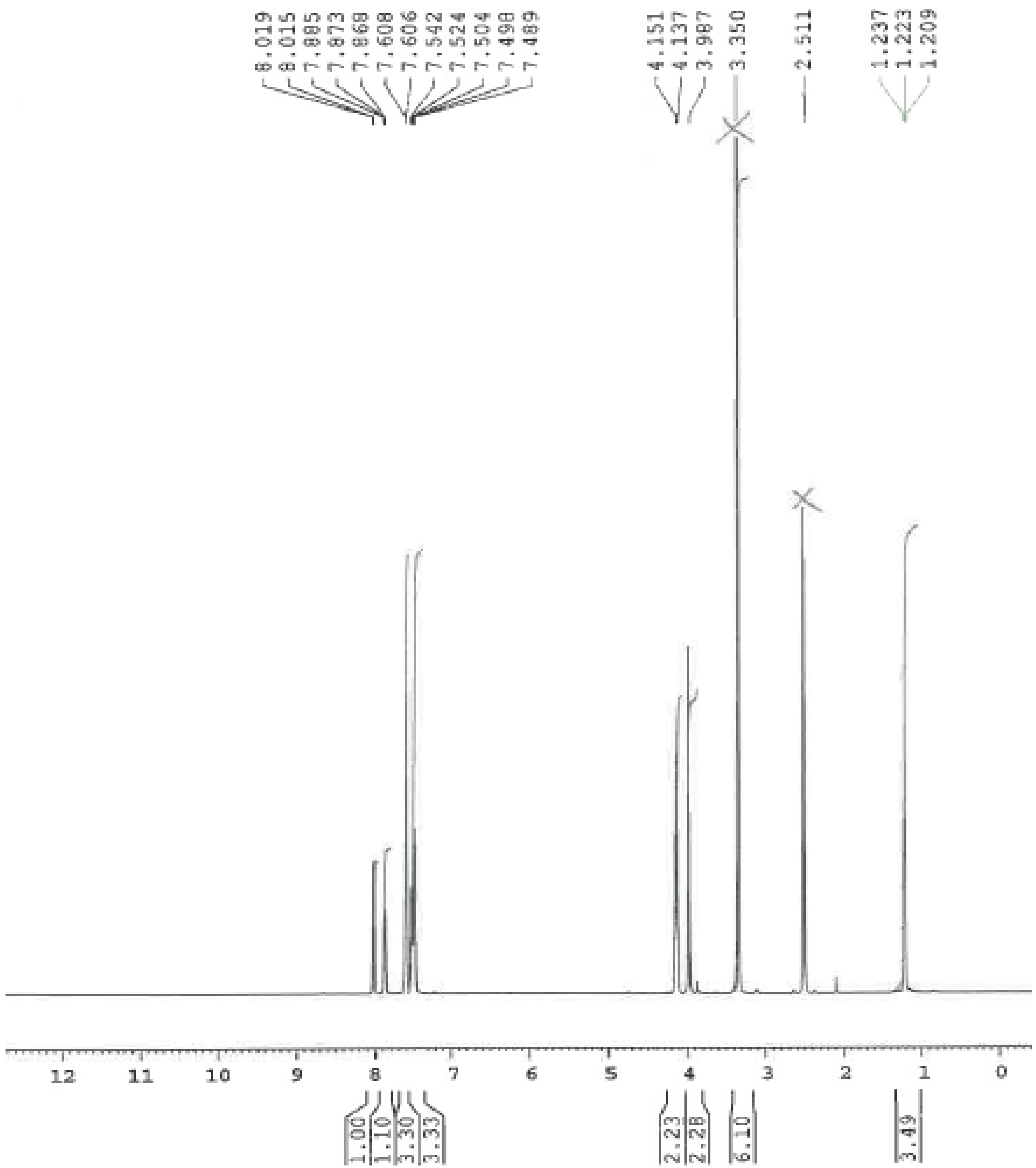

Figure S3. ^{1}HNMR of compound **2a (**extended**)**

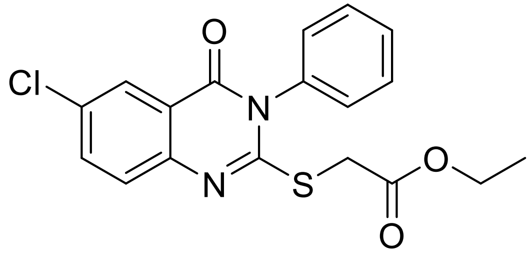

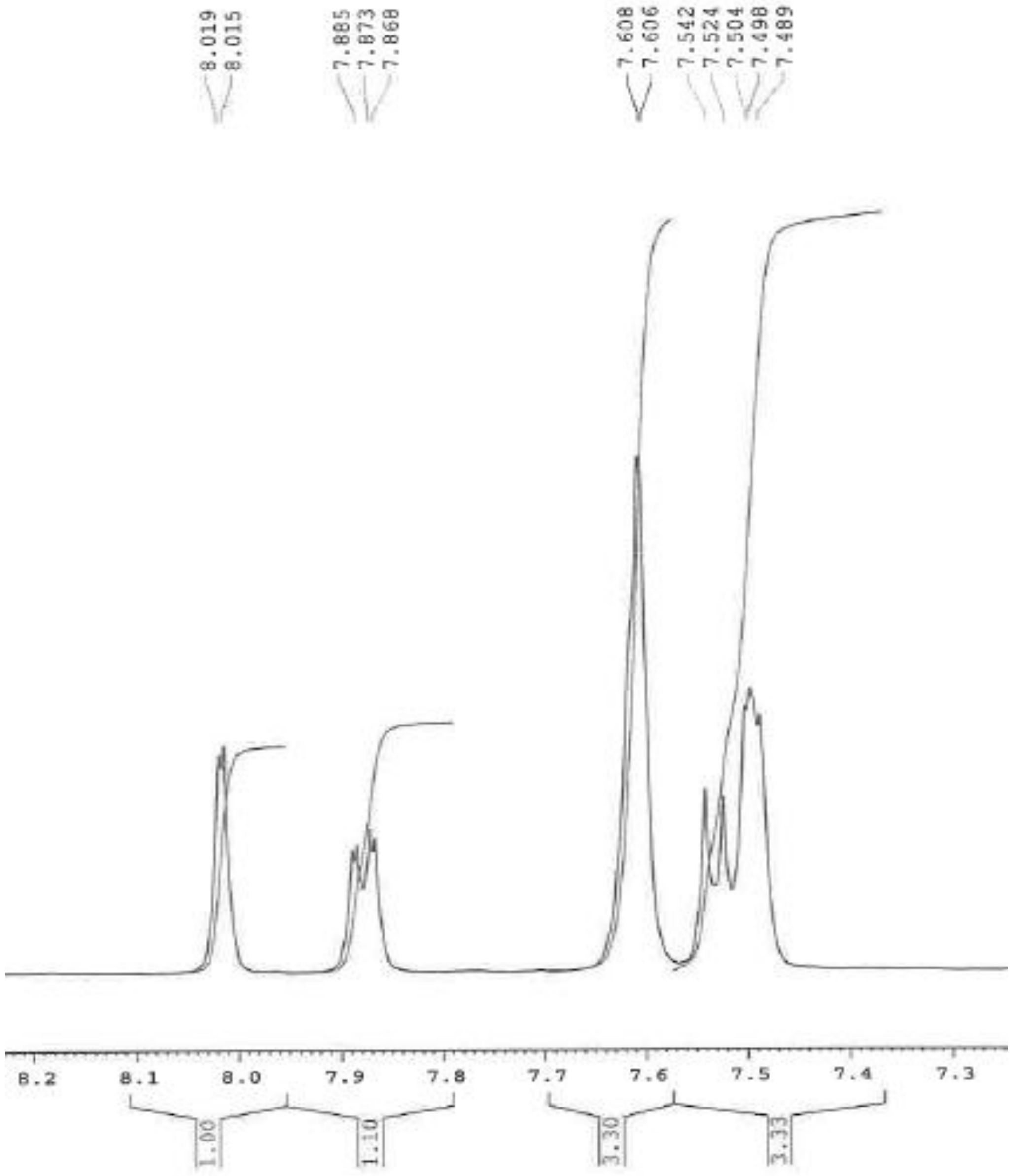

Figure S4. ^{1}HNMR of compound **2a (**extended**)**

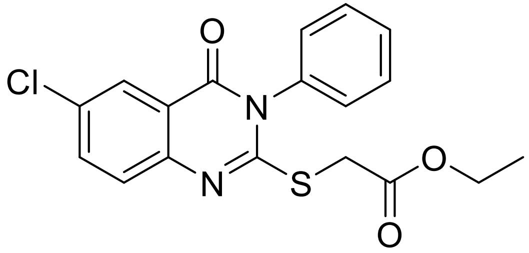

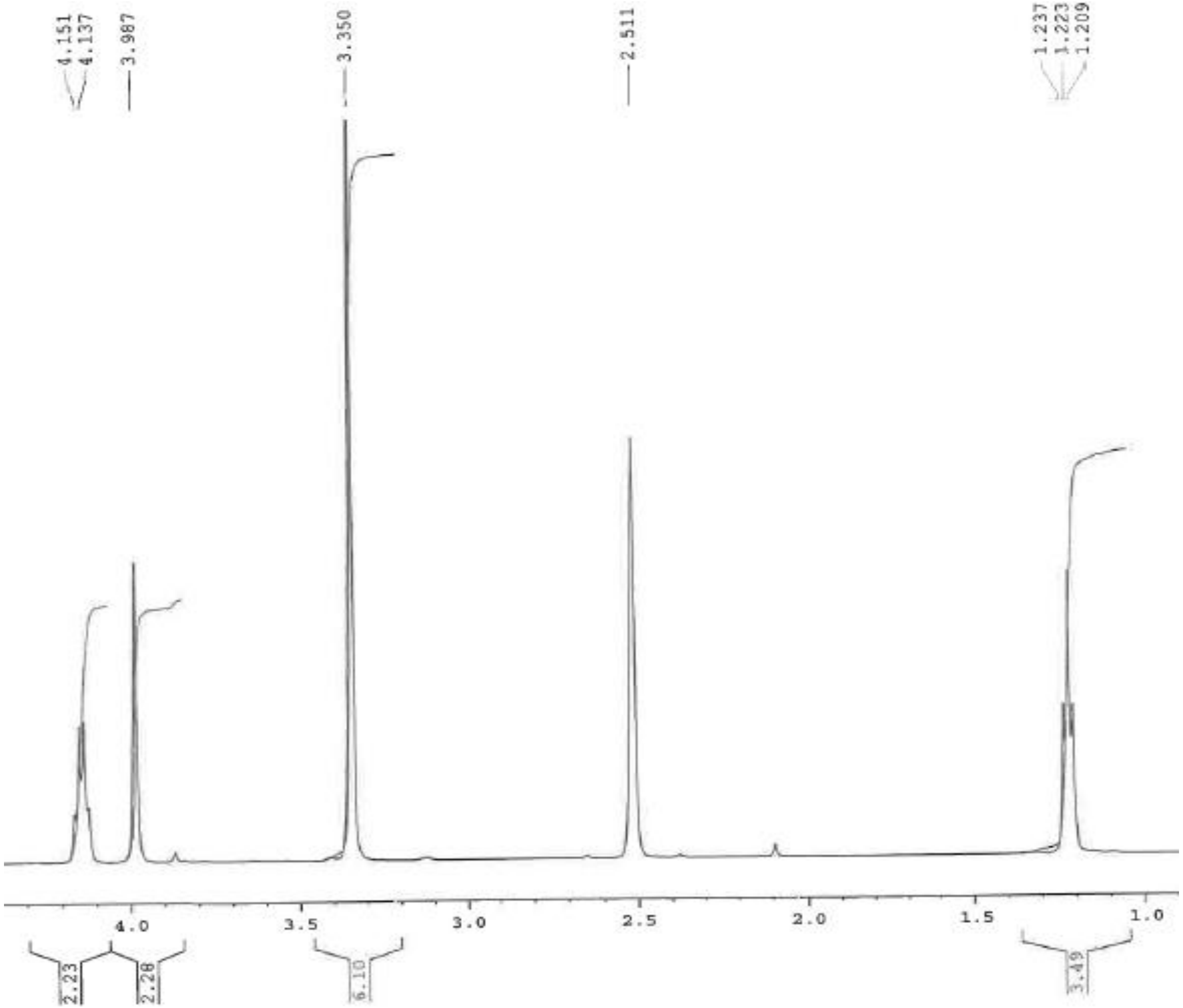

Figure S5. ^{13}CNMR of compound **2a**

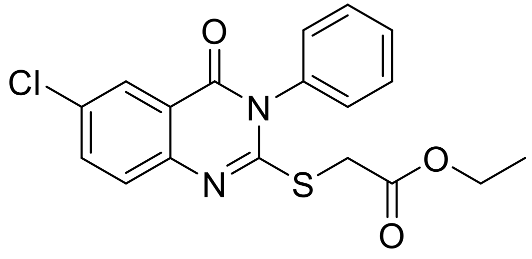

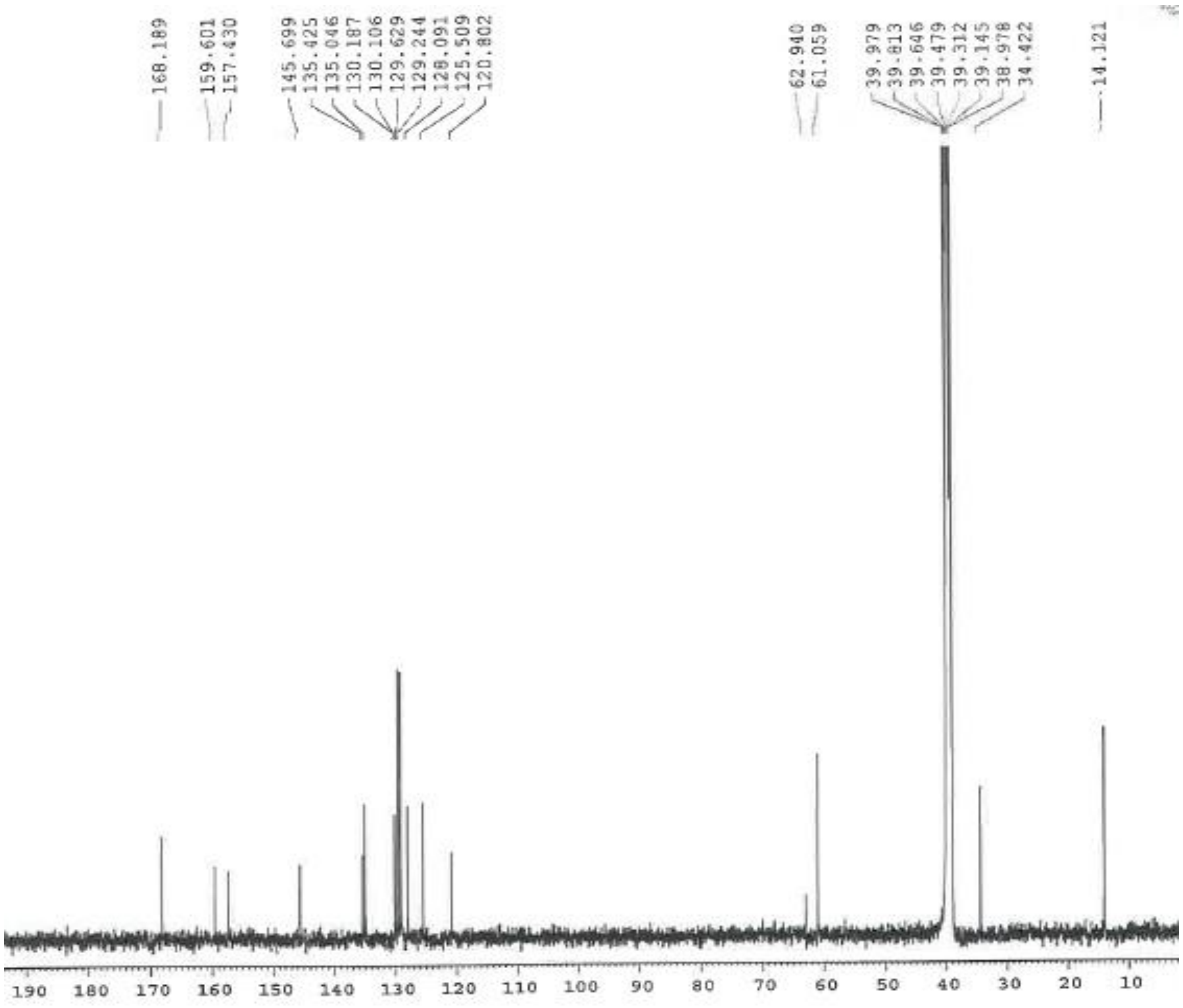

Figure S6. ^{13}CNMR of compound **2a** (extended)

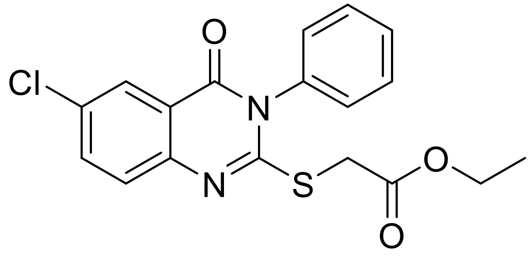

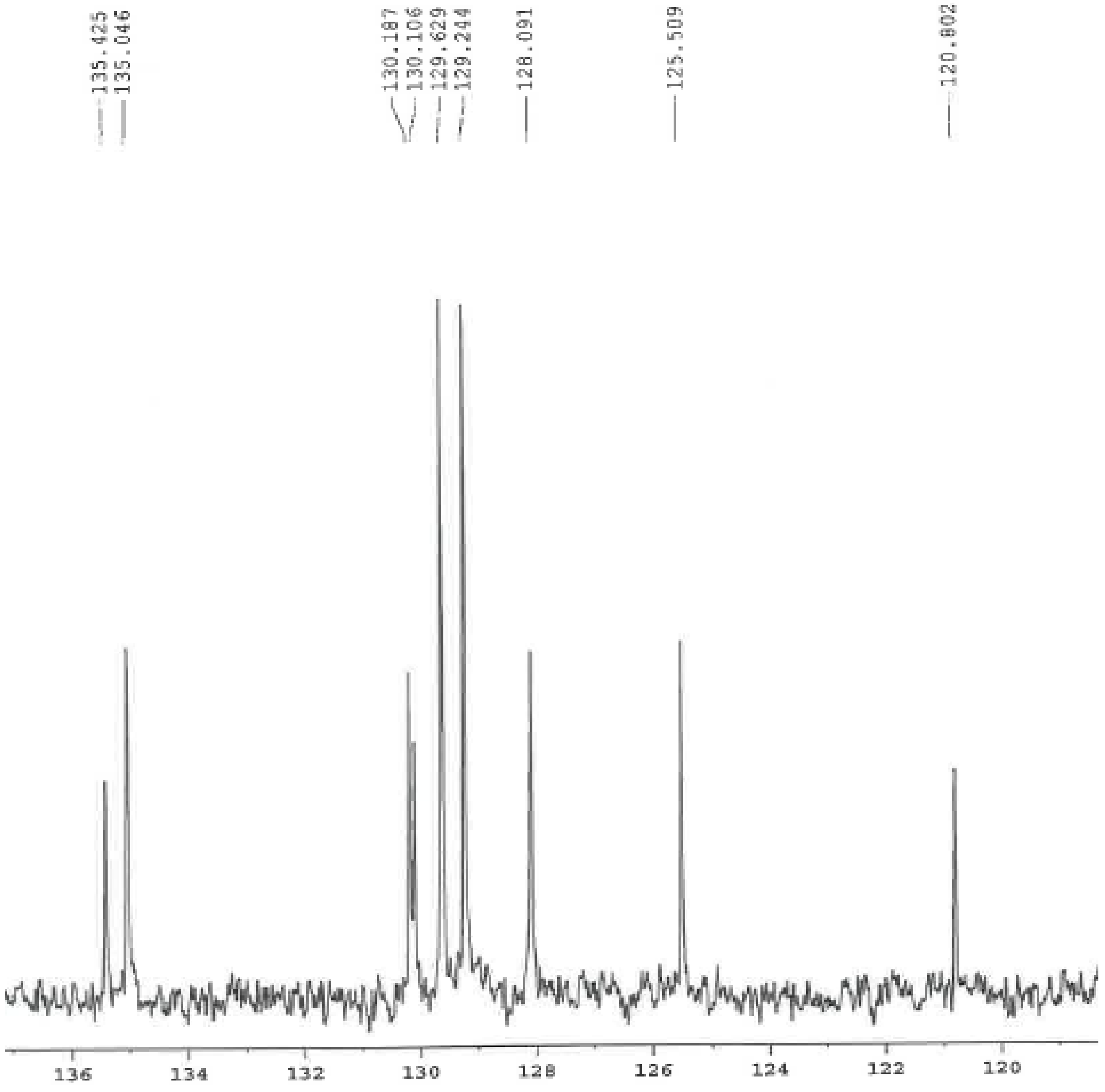

Figure S7. ^{1}HNMR of compound **2b**

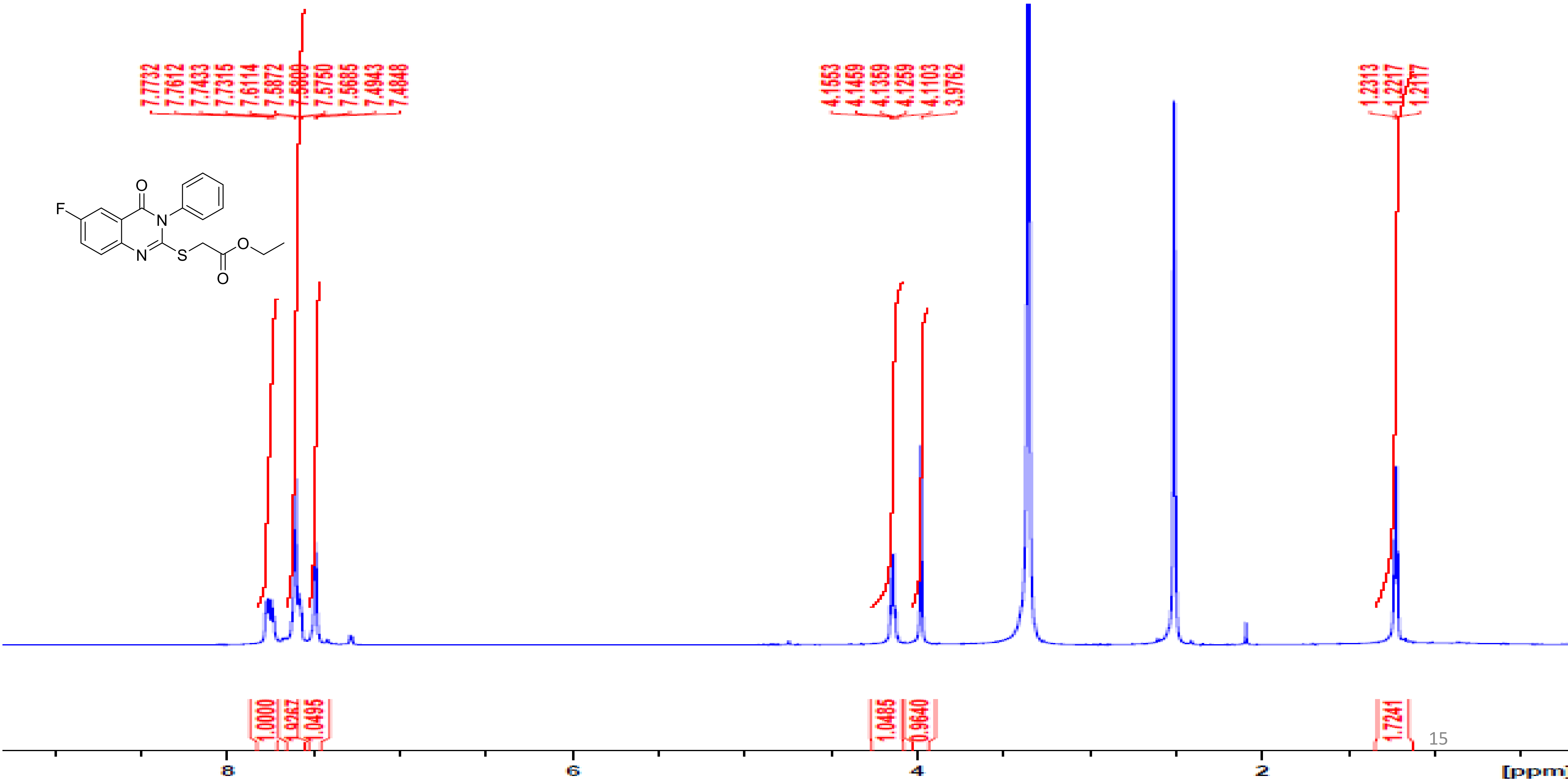

Figure S8. ^{13}CNMR of compound **2b**

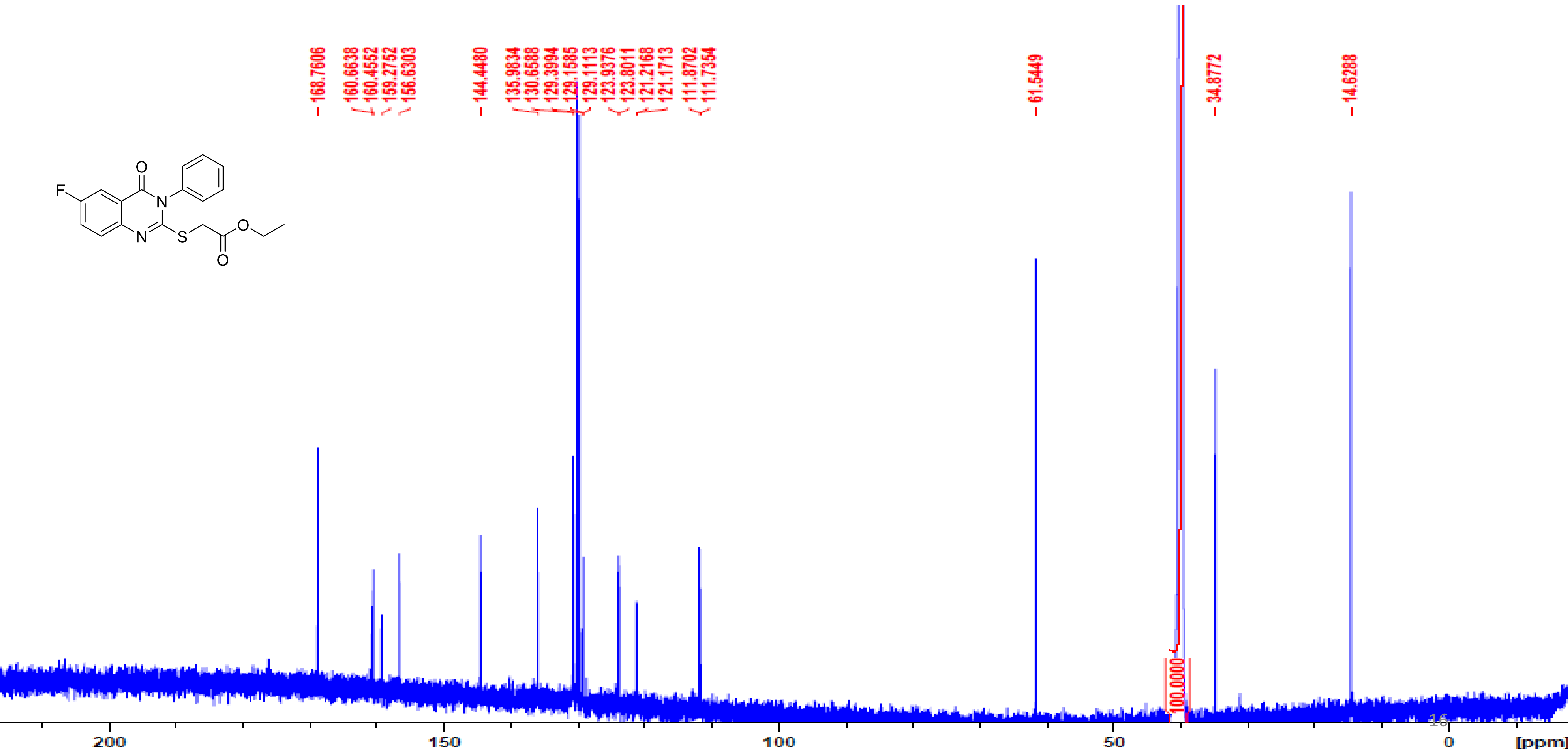

Figure S9. ^{13}CNMR of compound **2b (**extended**)**

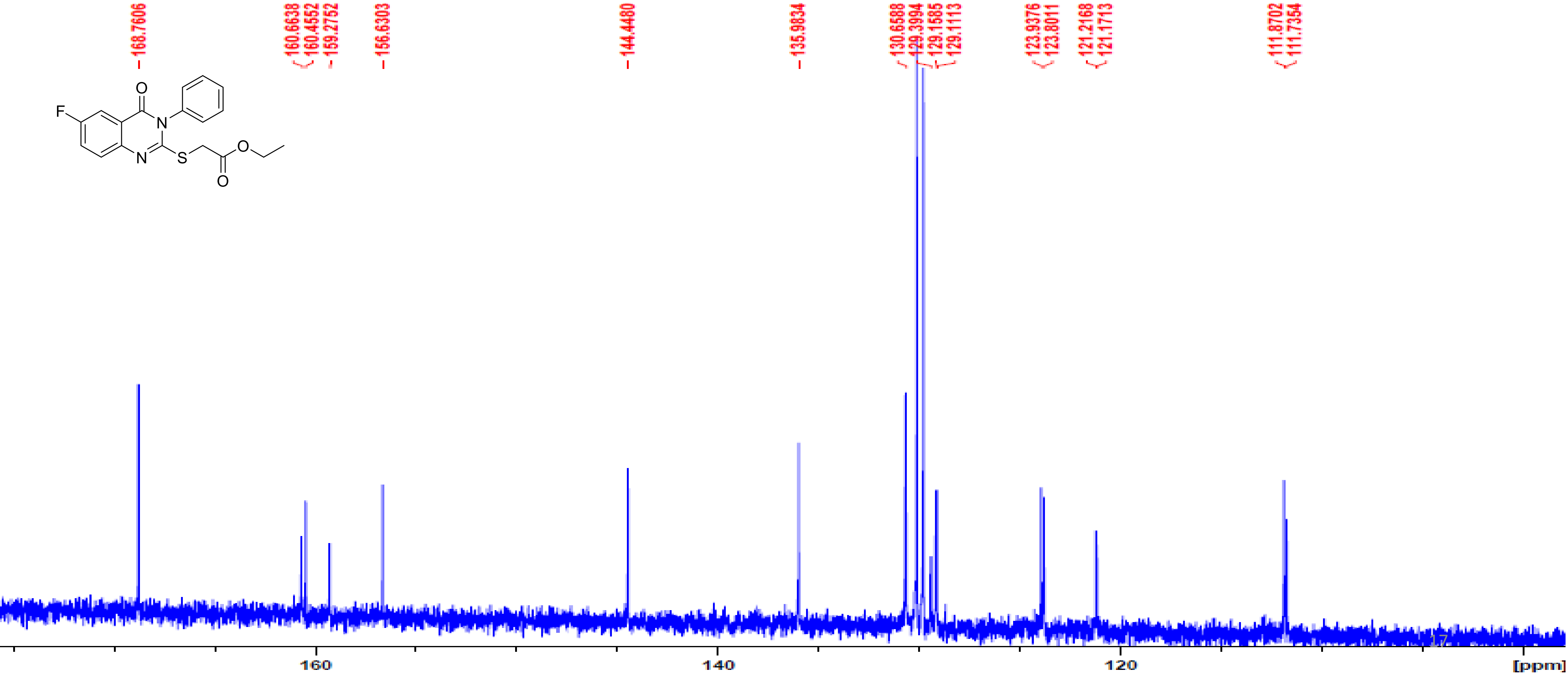

Figure S10. ^{1}HNMR of compound **2c**

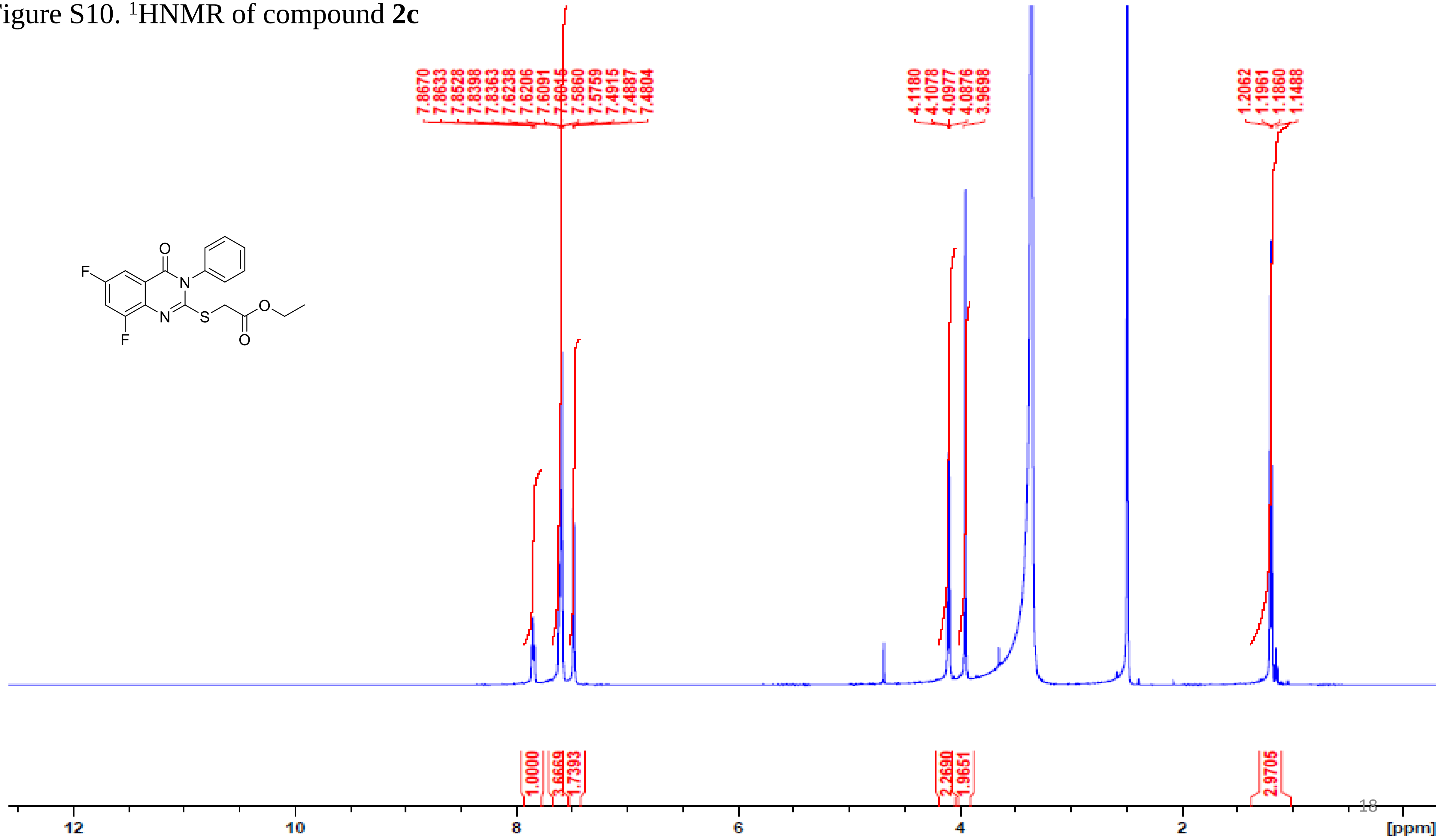

Figure S11. ^{1}HNMR of compound **2c (**extended**)**

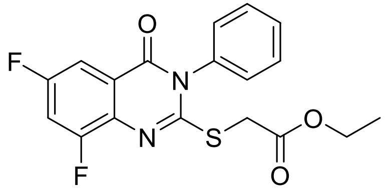

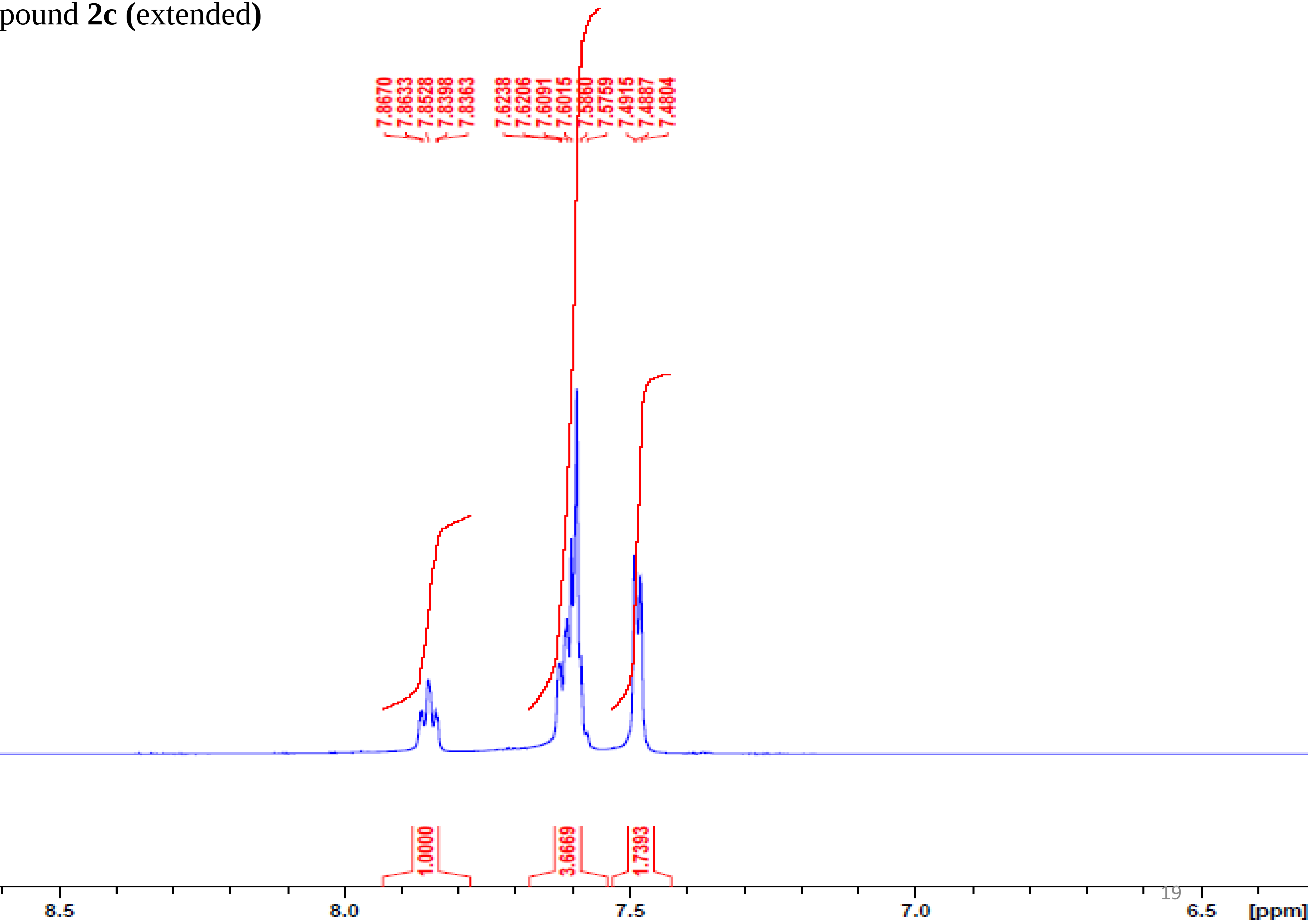

Figure S12. ^{13}CNMR of compound **2c**

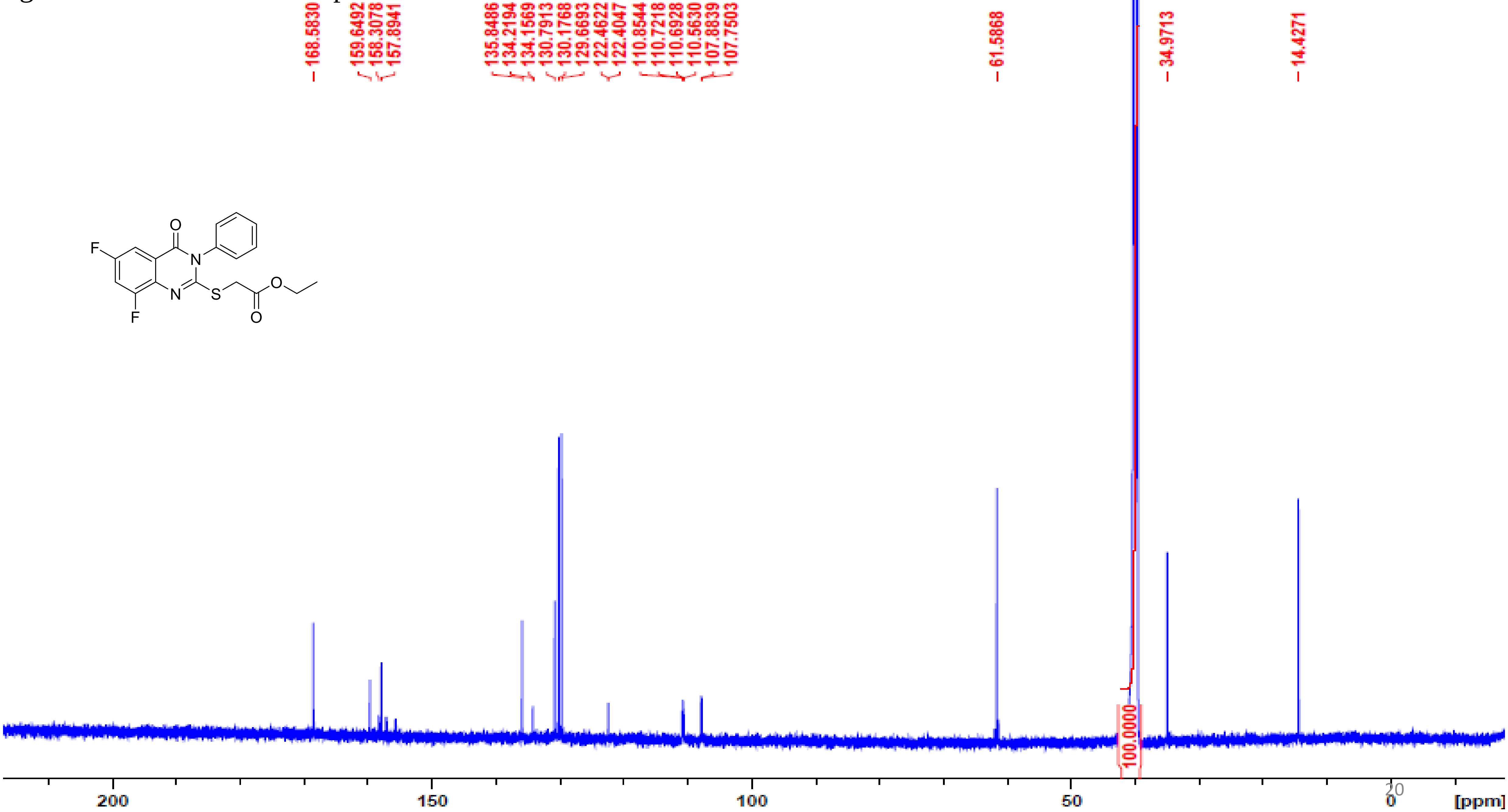

Figure S13. ^{13}CNMR of compound **2c (**extended**)**

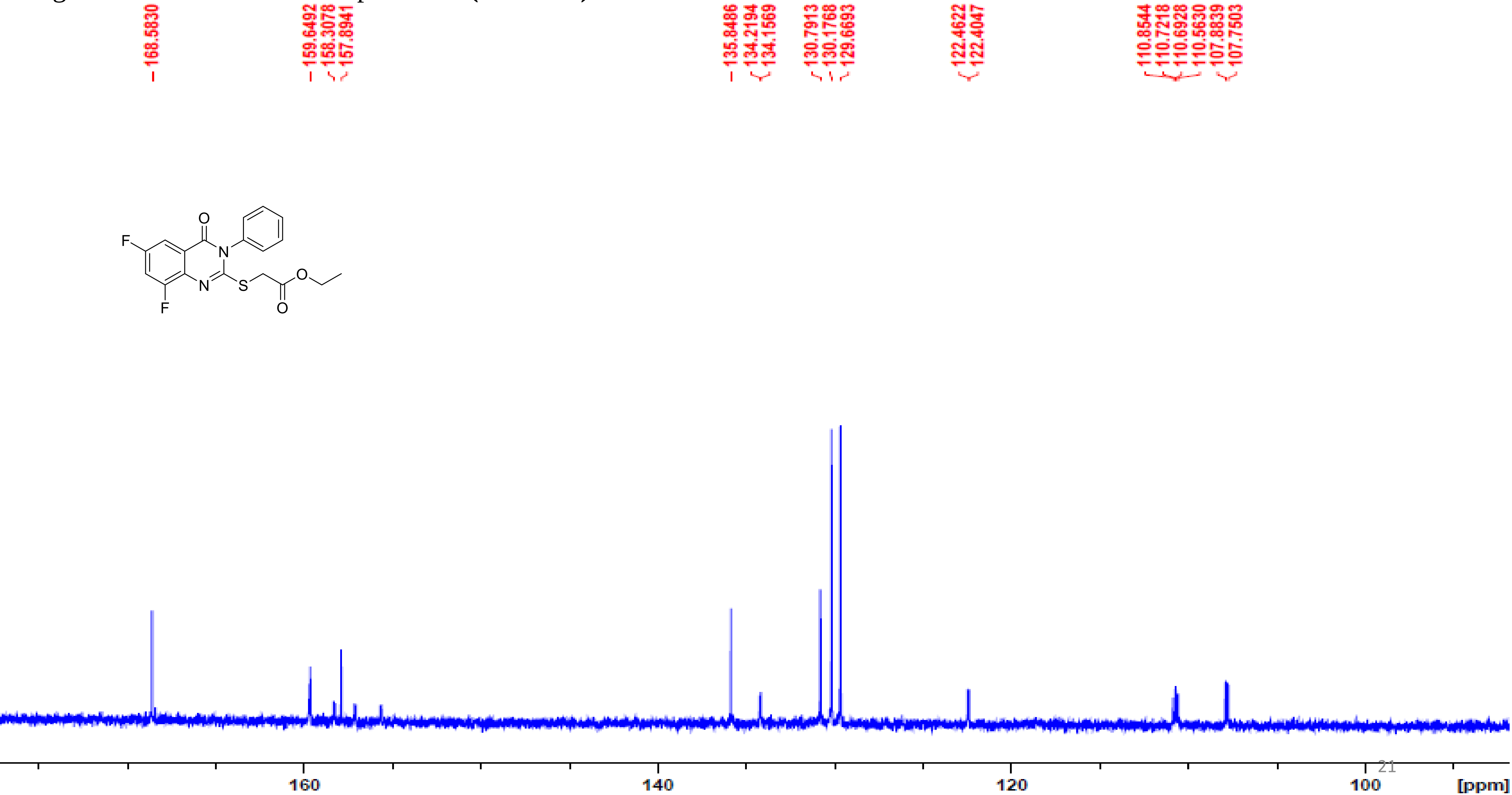

Figure S14. ^{1}HNMR of compound **2d**

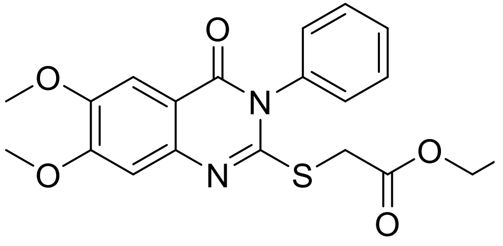

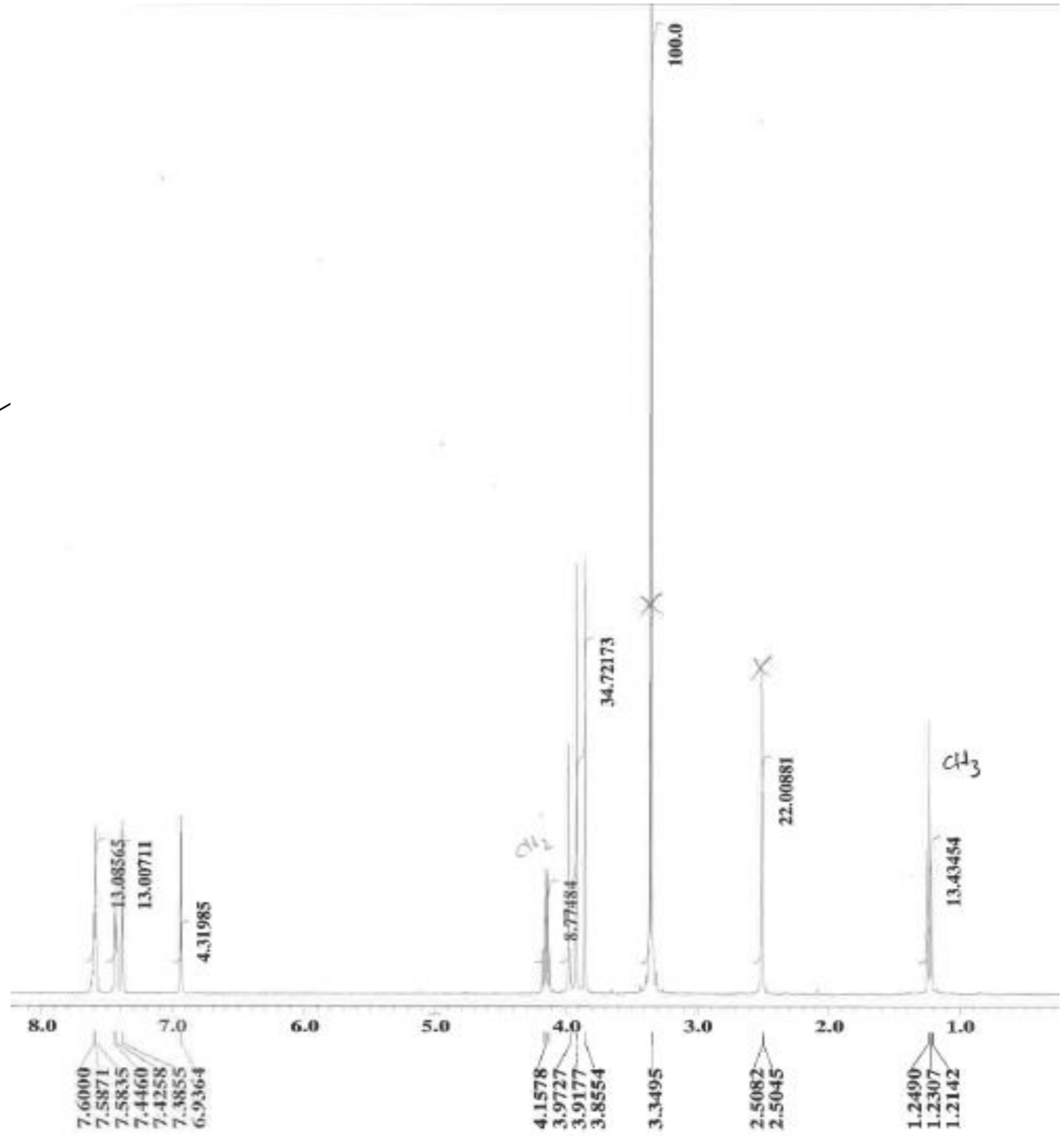

Figure S15. ^{1}HNMR of compound **2d (**extended**)**

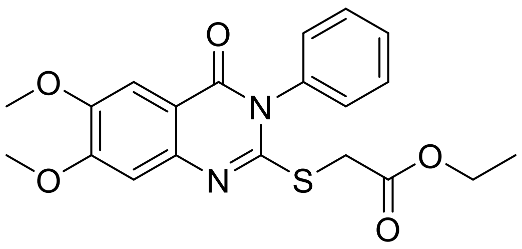

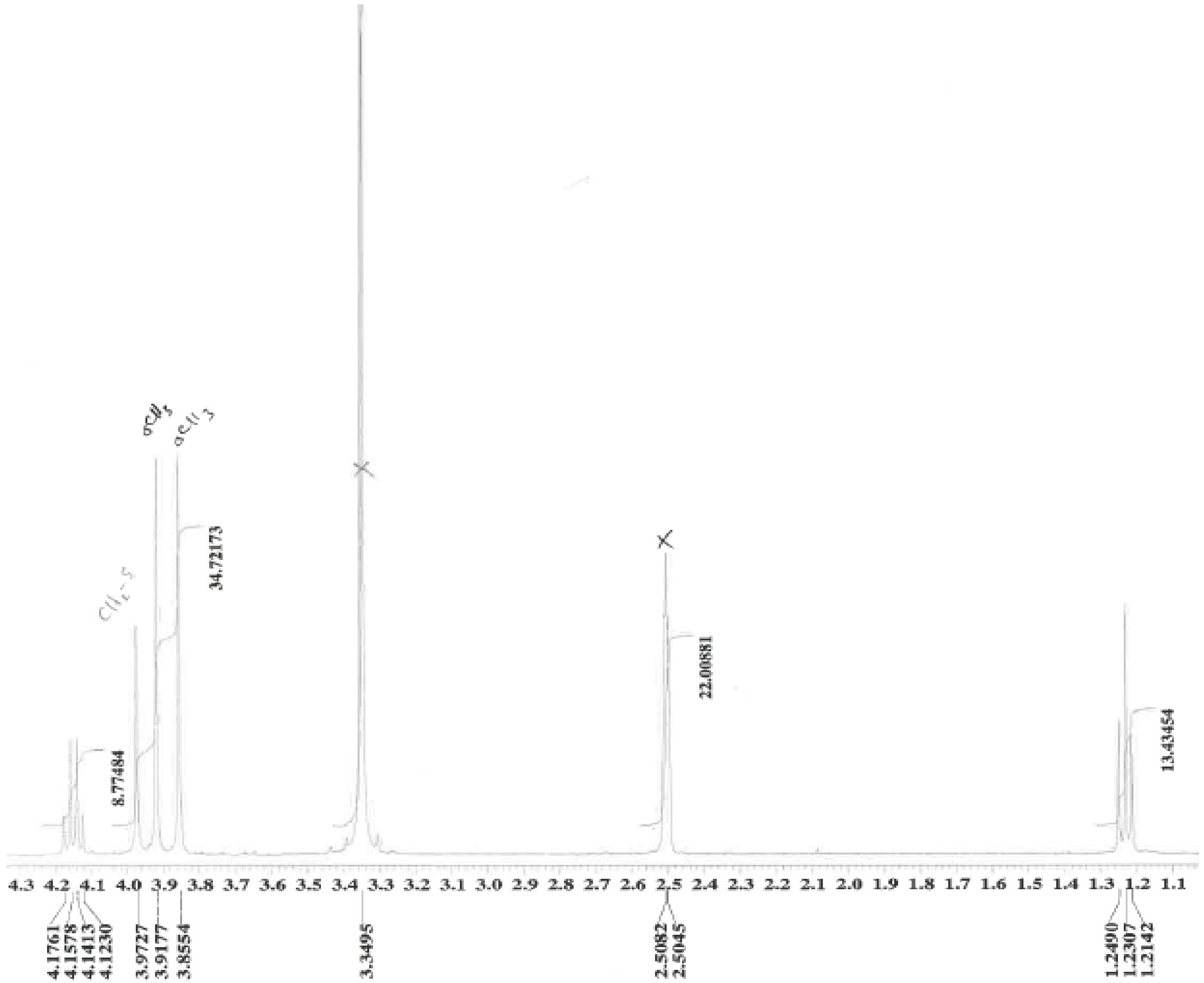

Figure S16. ^{1}HNMR of compound **2d (**extended**)**

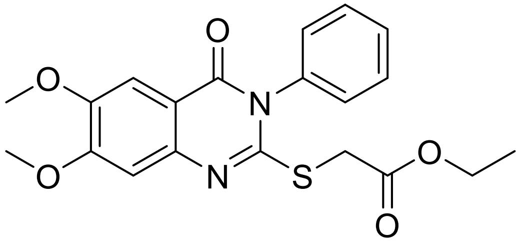

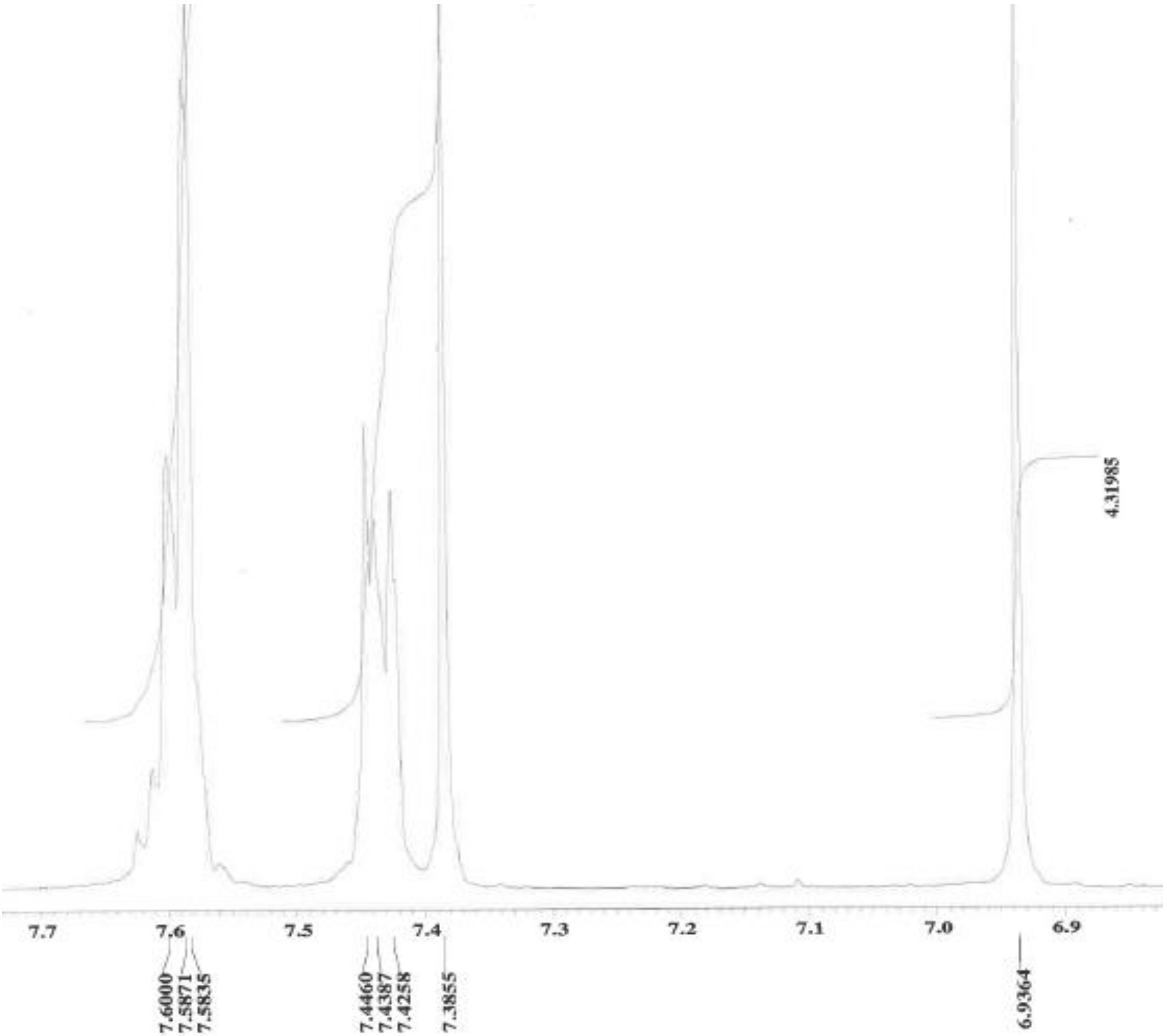

Figure S17. ^{13}CNMR of compound **2d**

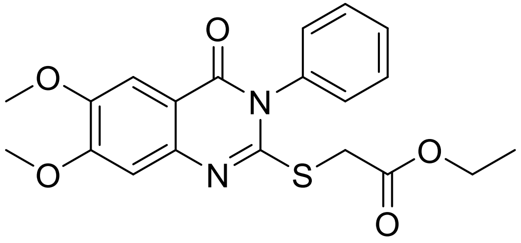

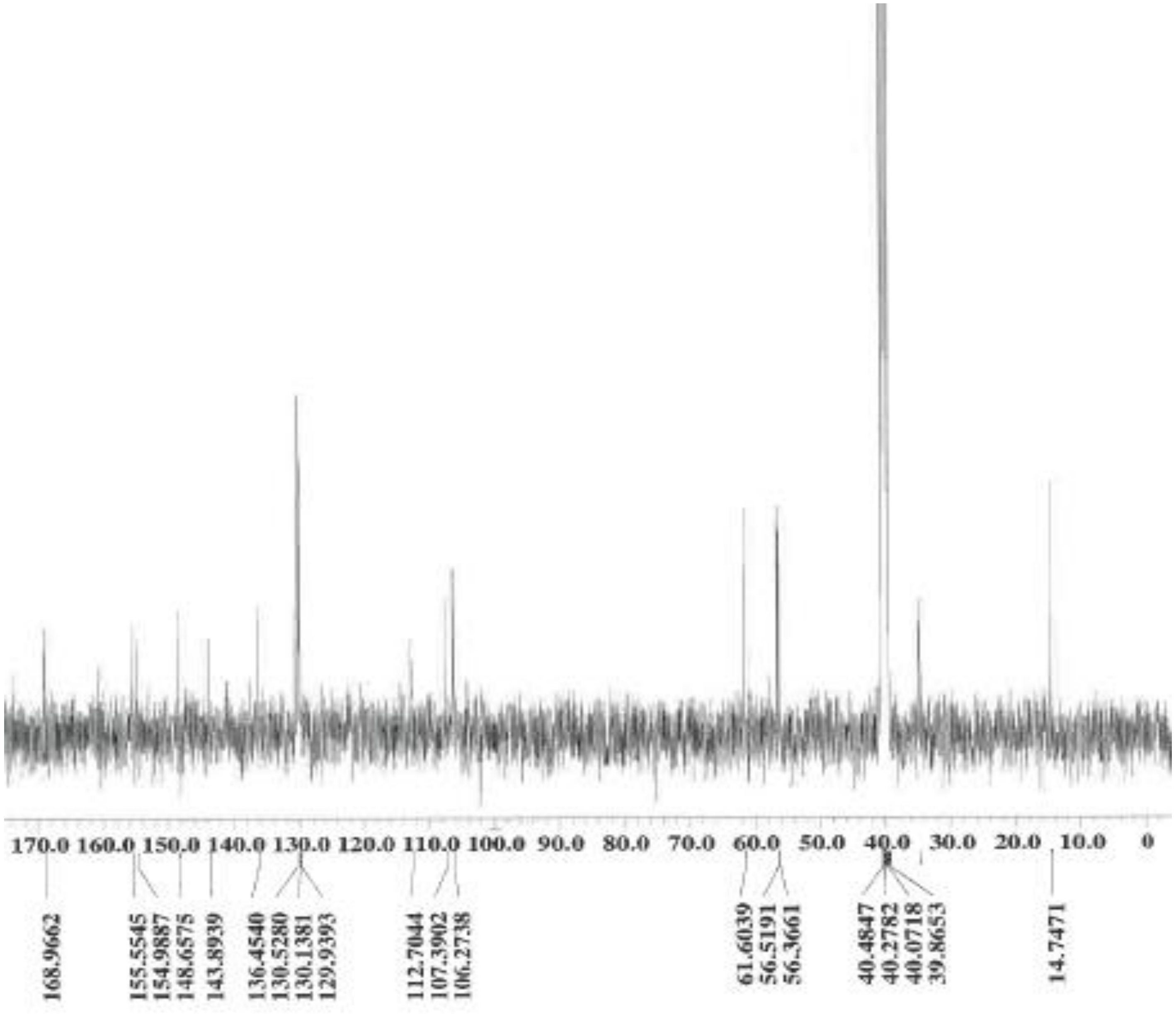

Figure S18. ^{13}CNMR of compound **2d** (extended)

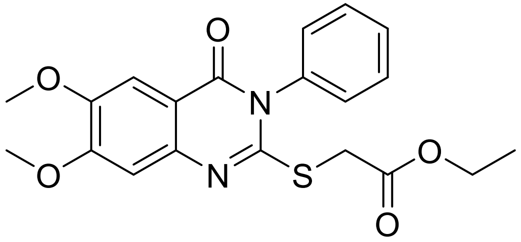

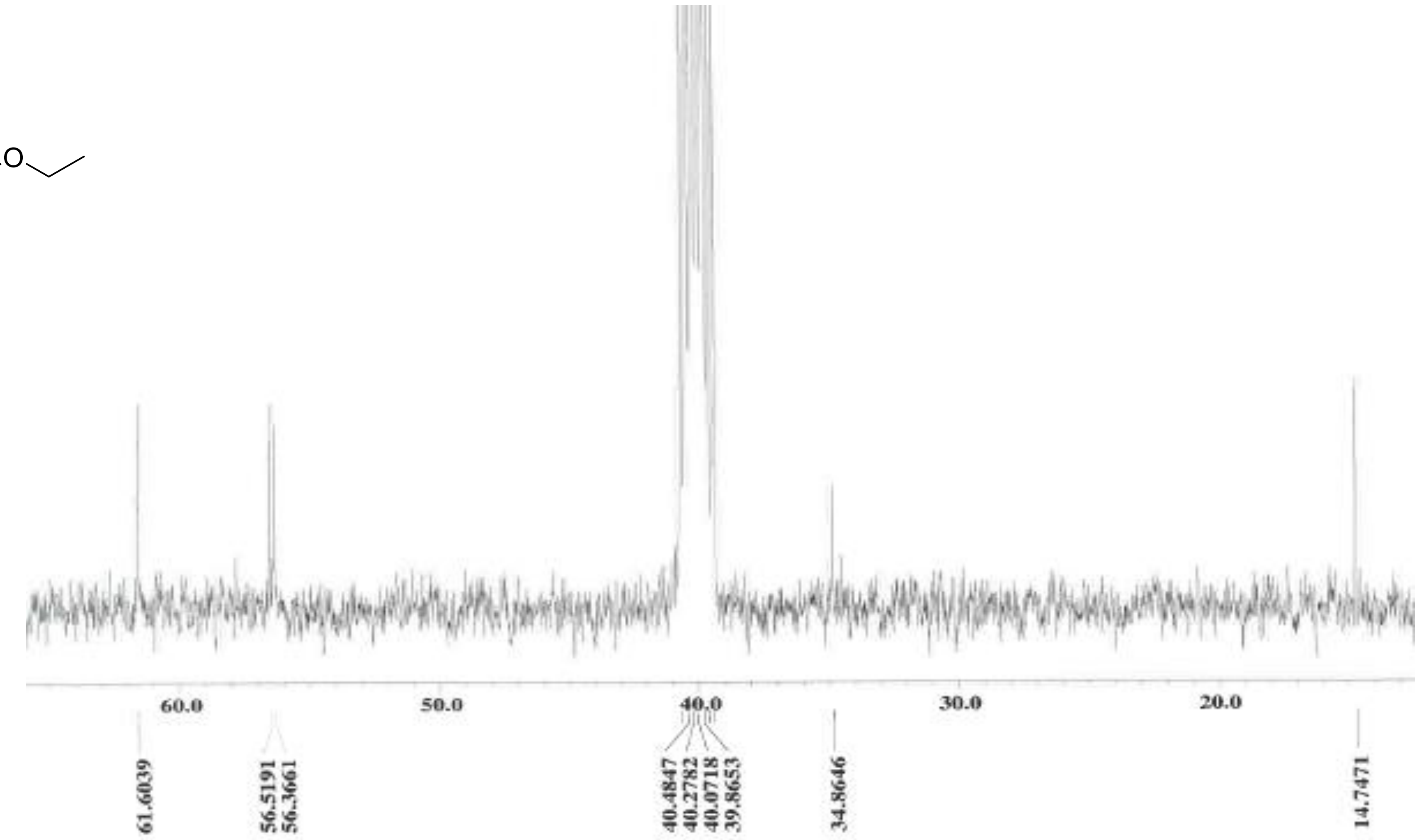

Figure S19. ^{13}CNMR of compound **2d** (extended)

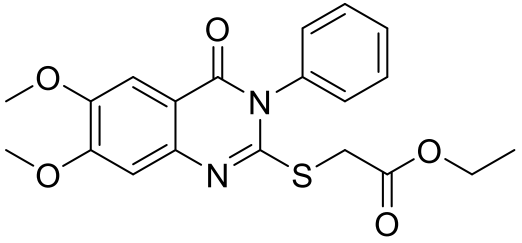

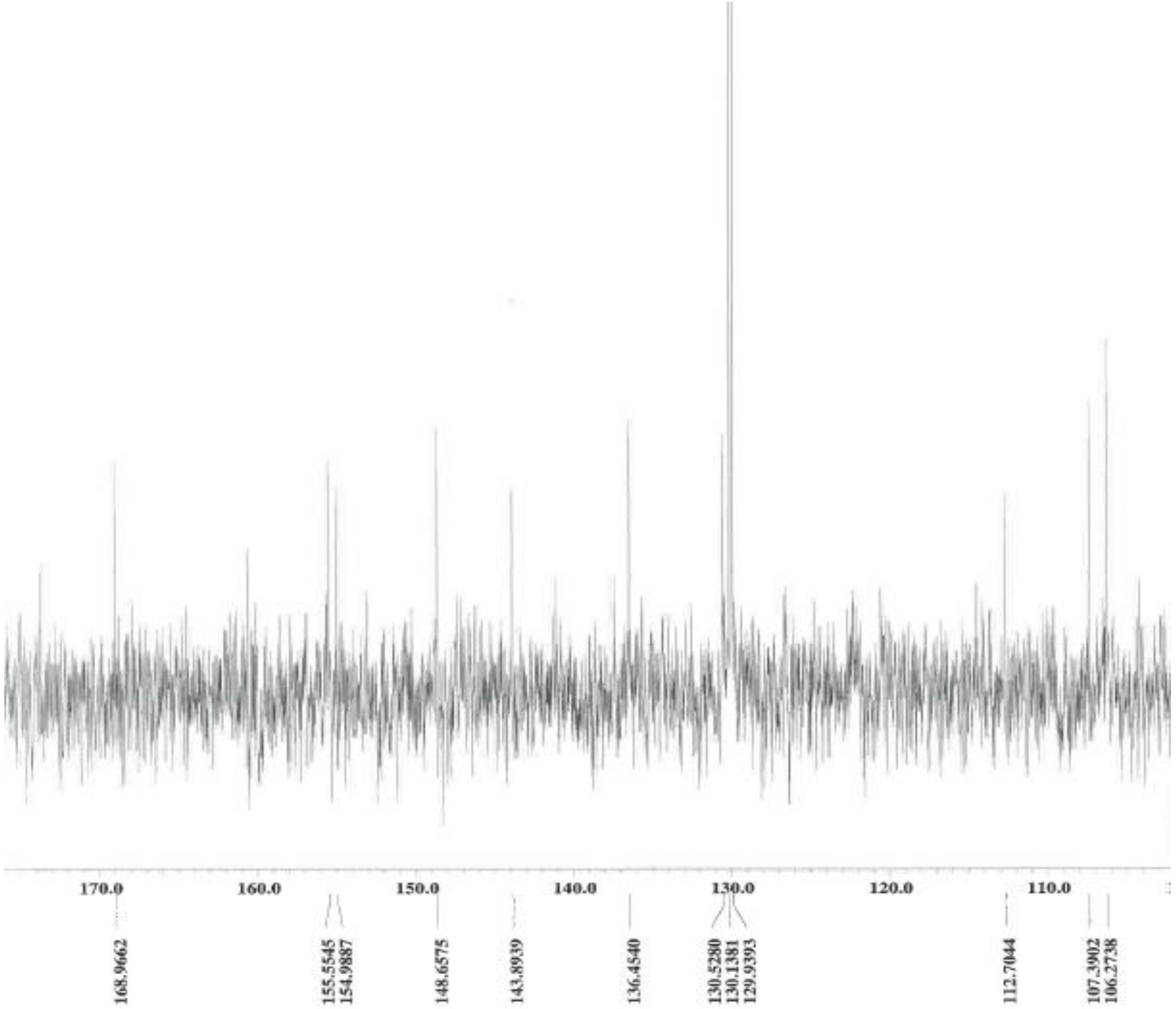

Figure S20. ^{1}HNMR of compound **2e**

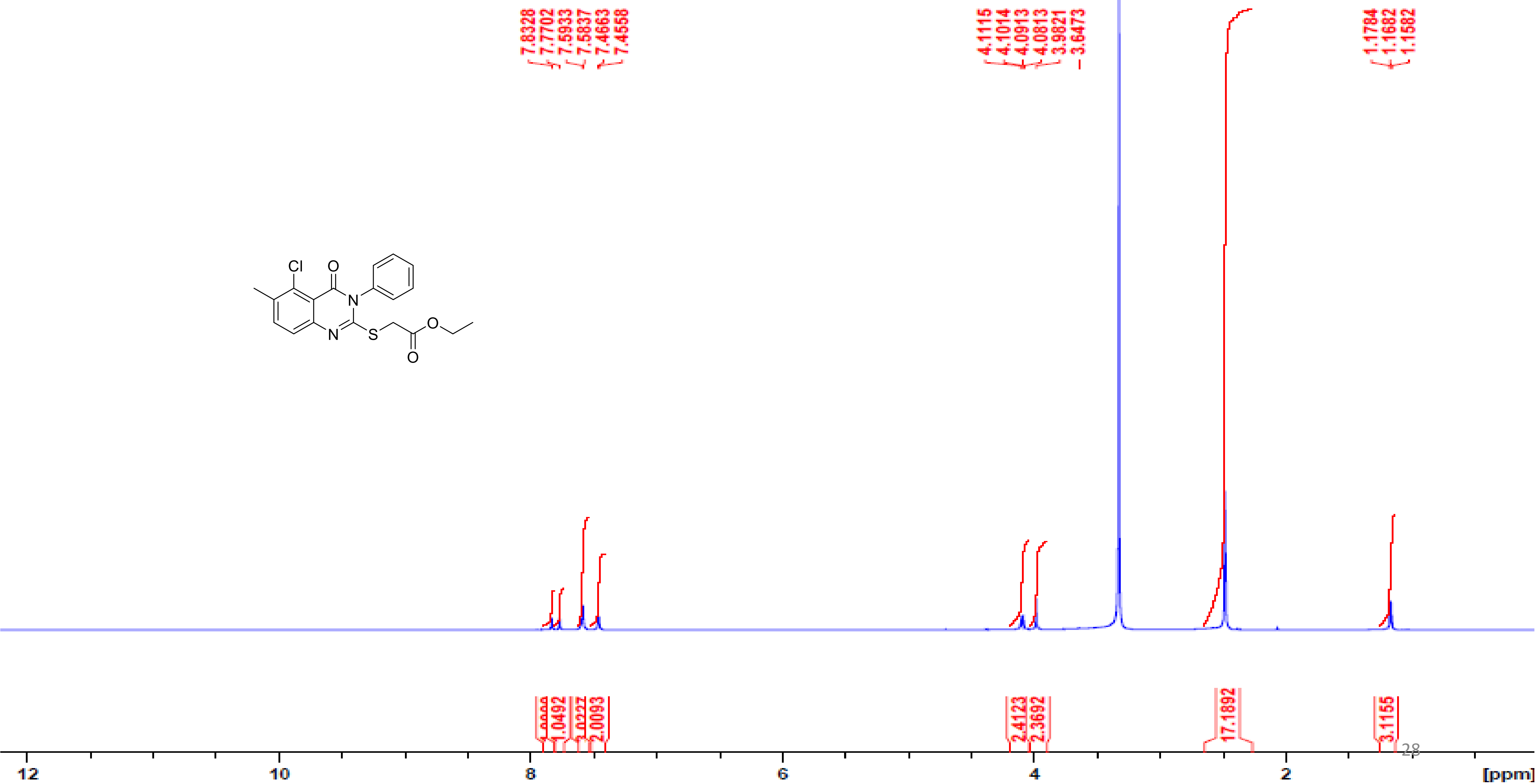

Figure S21. ^{1}HNMR of compound **2e (**extended**)**

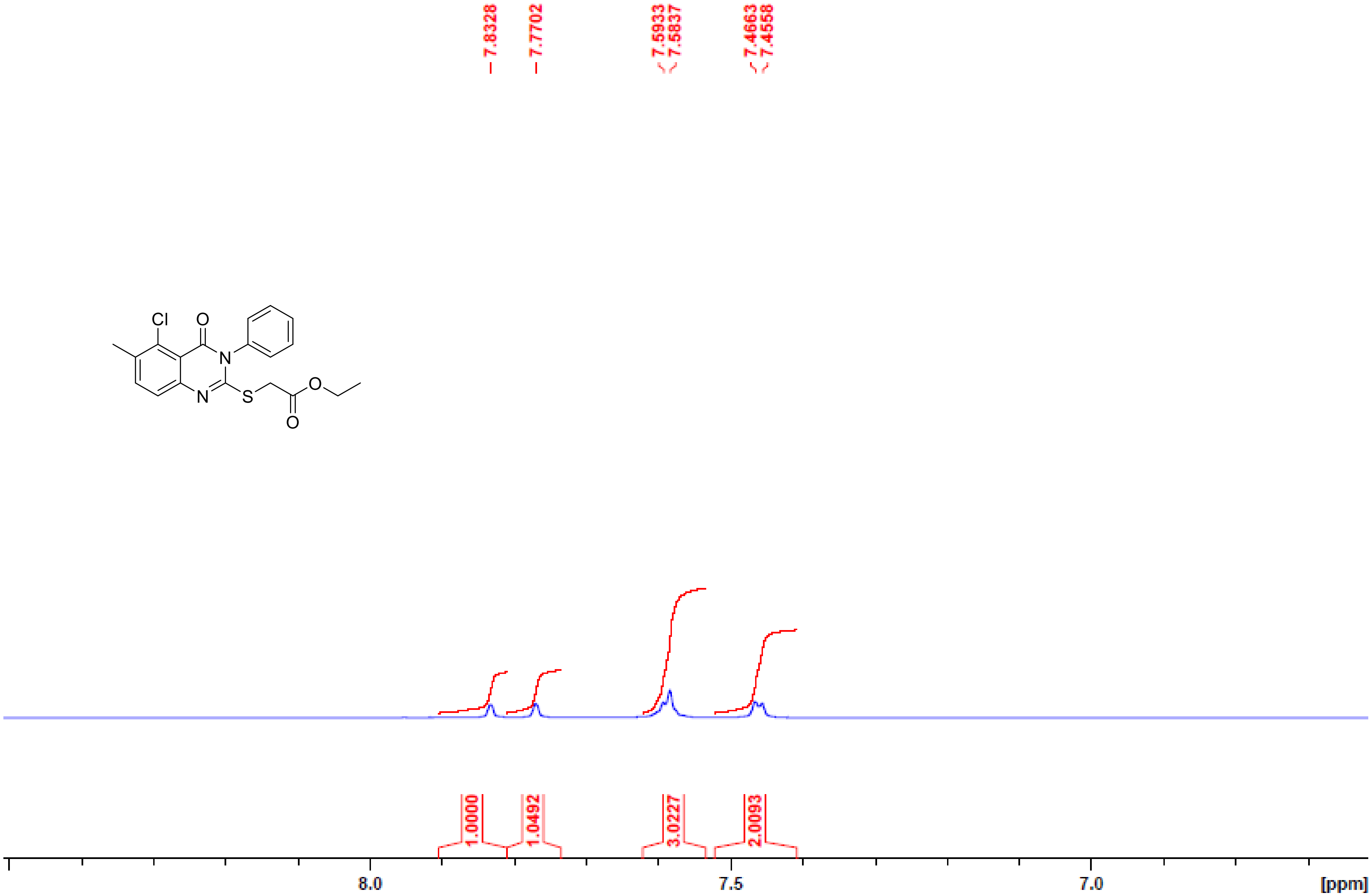

Figure S22. ^{13}CNMR of compound **2e**

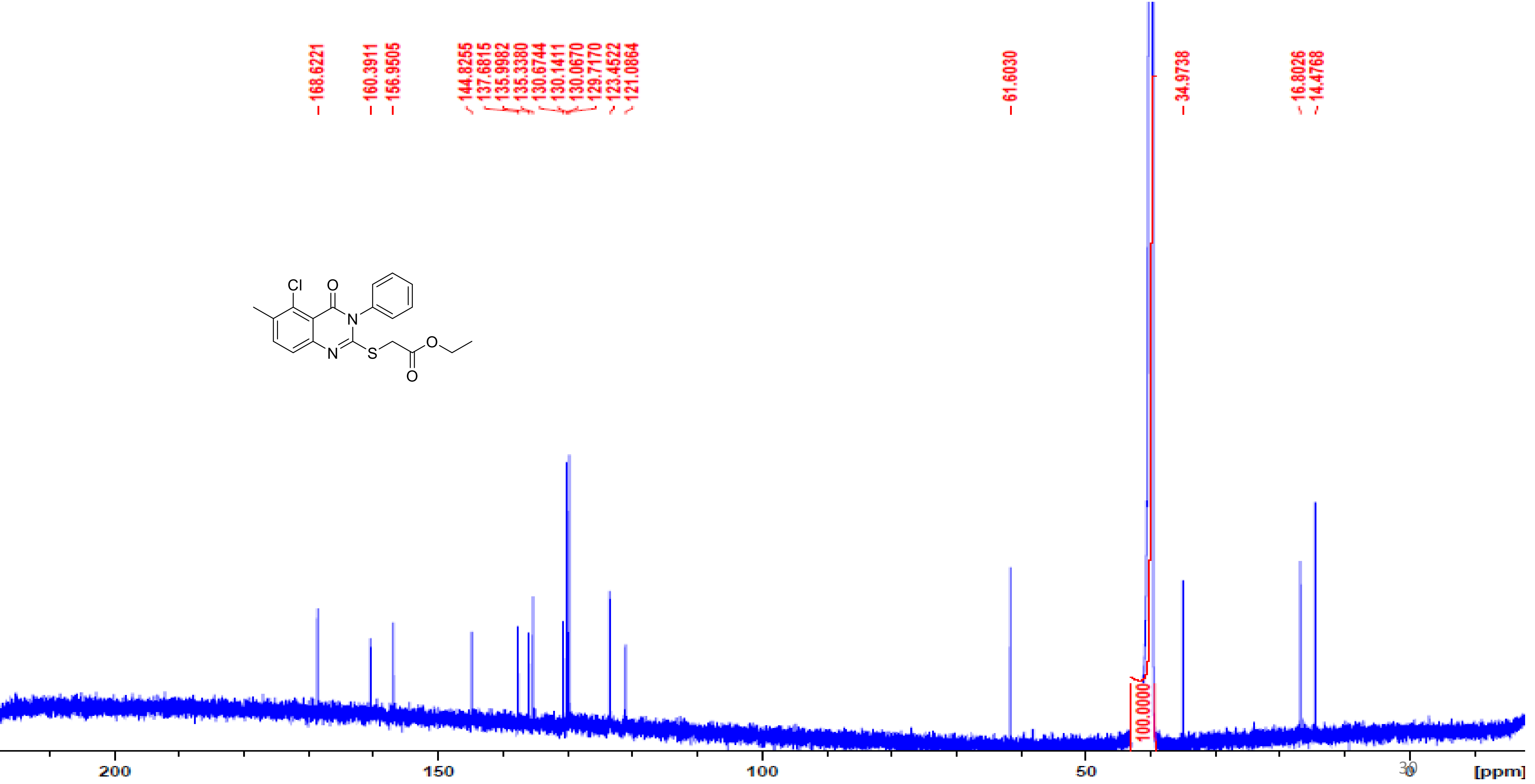

Figure S23. ^{13}CNMR of compound **2e** (extended)

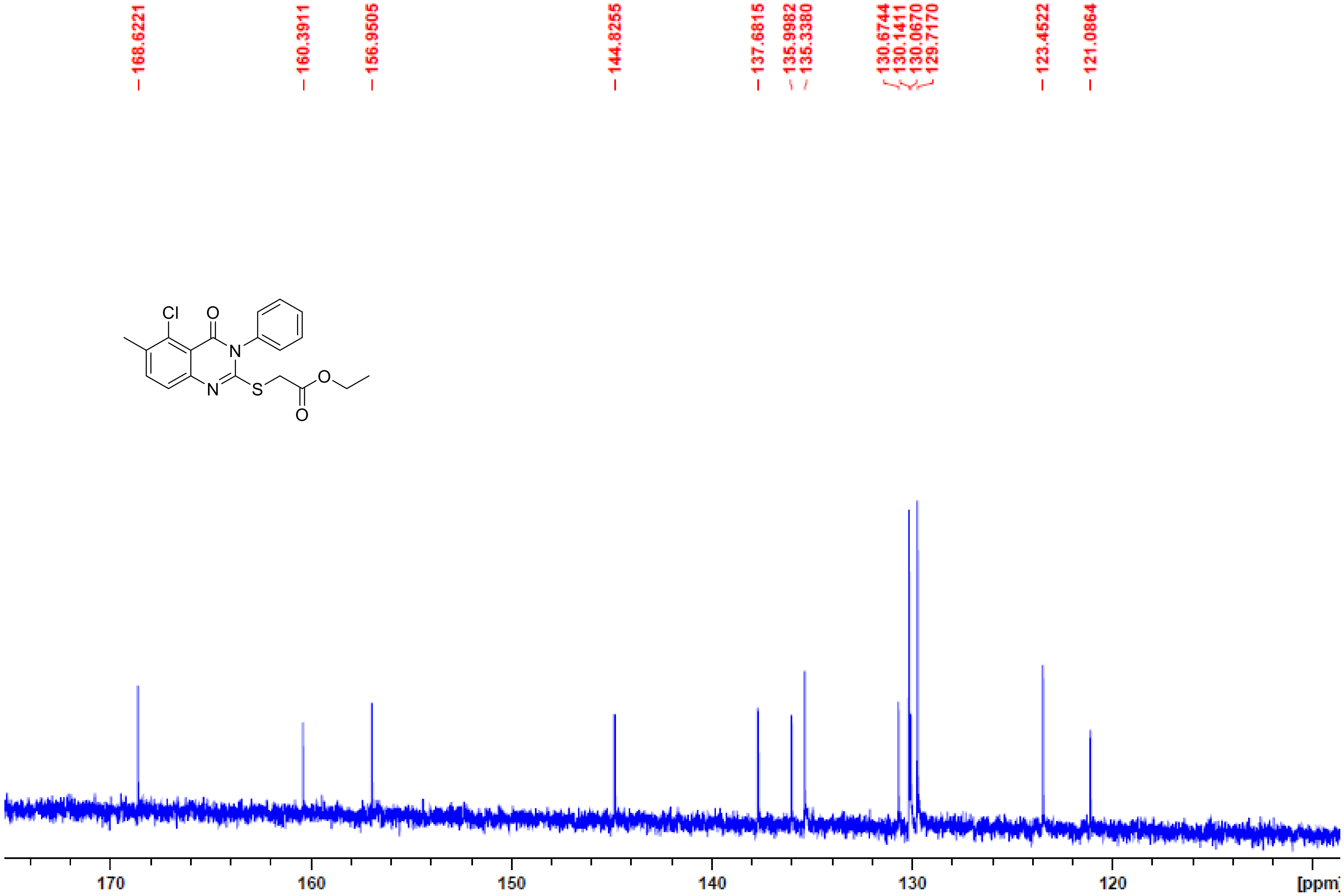

Figure S24. ^{1}HNMR of compound **2f**

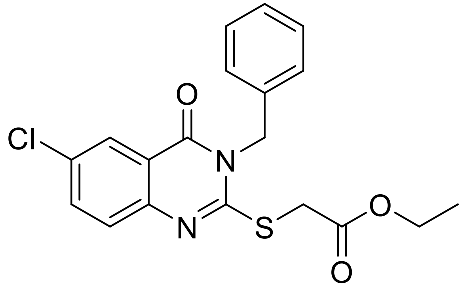

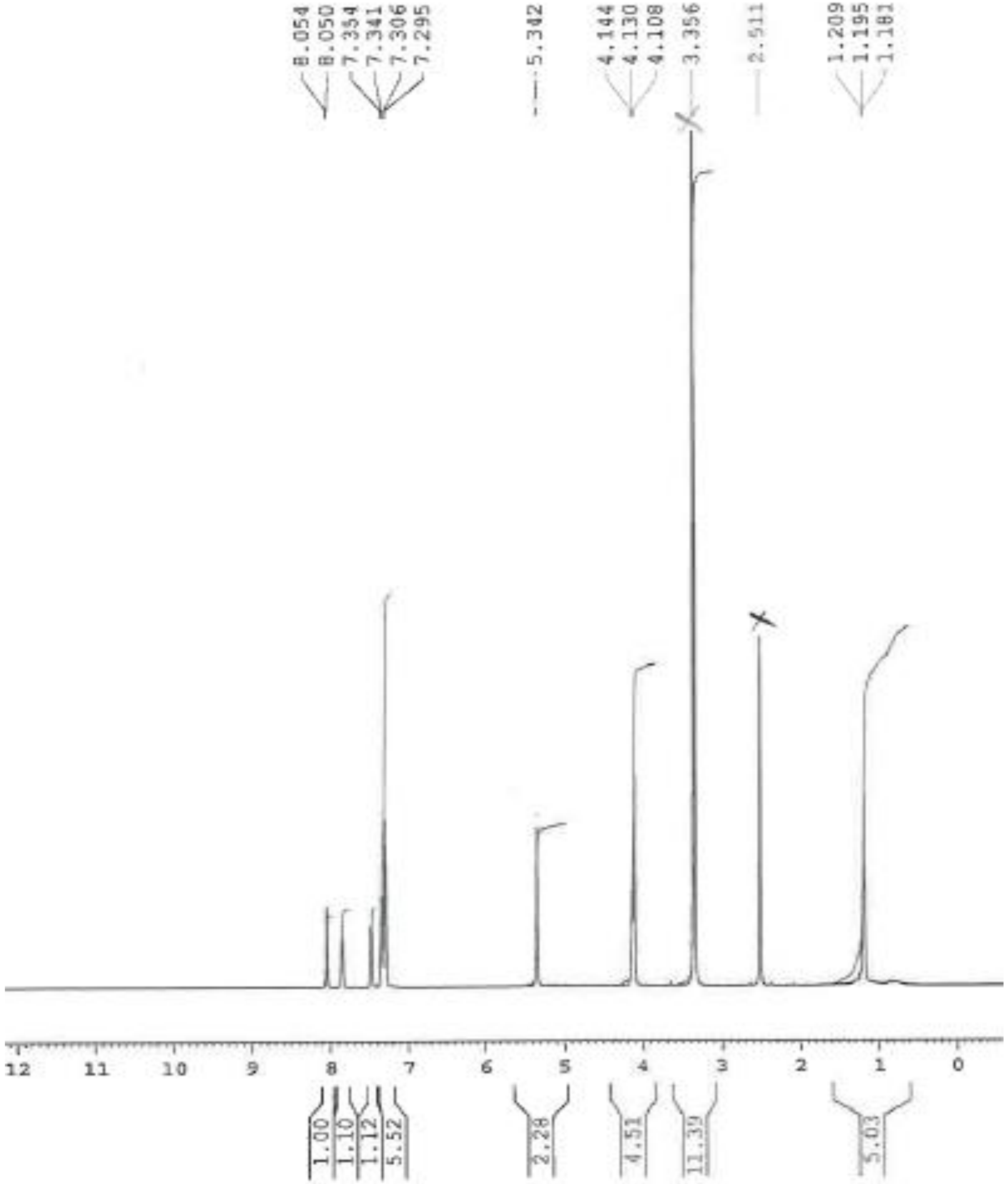

Figure S25. ^{1}HNMR of compound **2f (**extended**)**

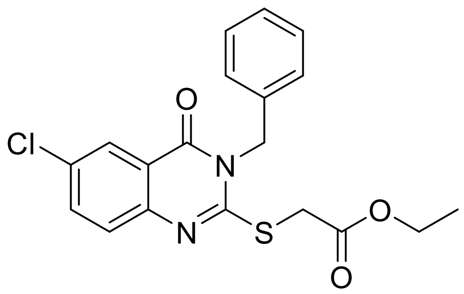

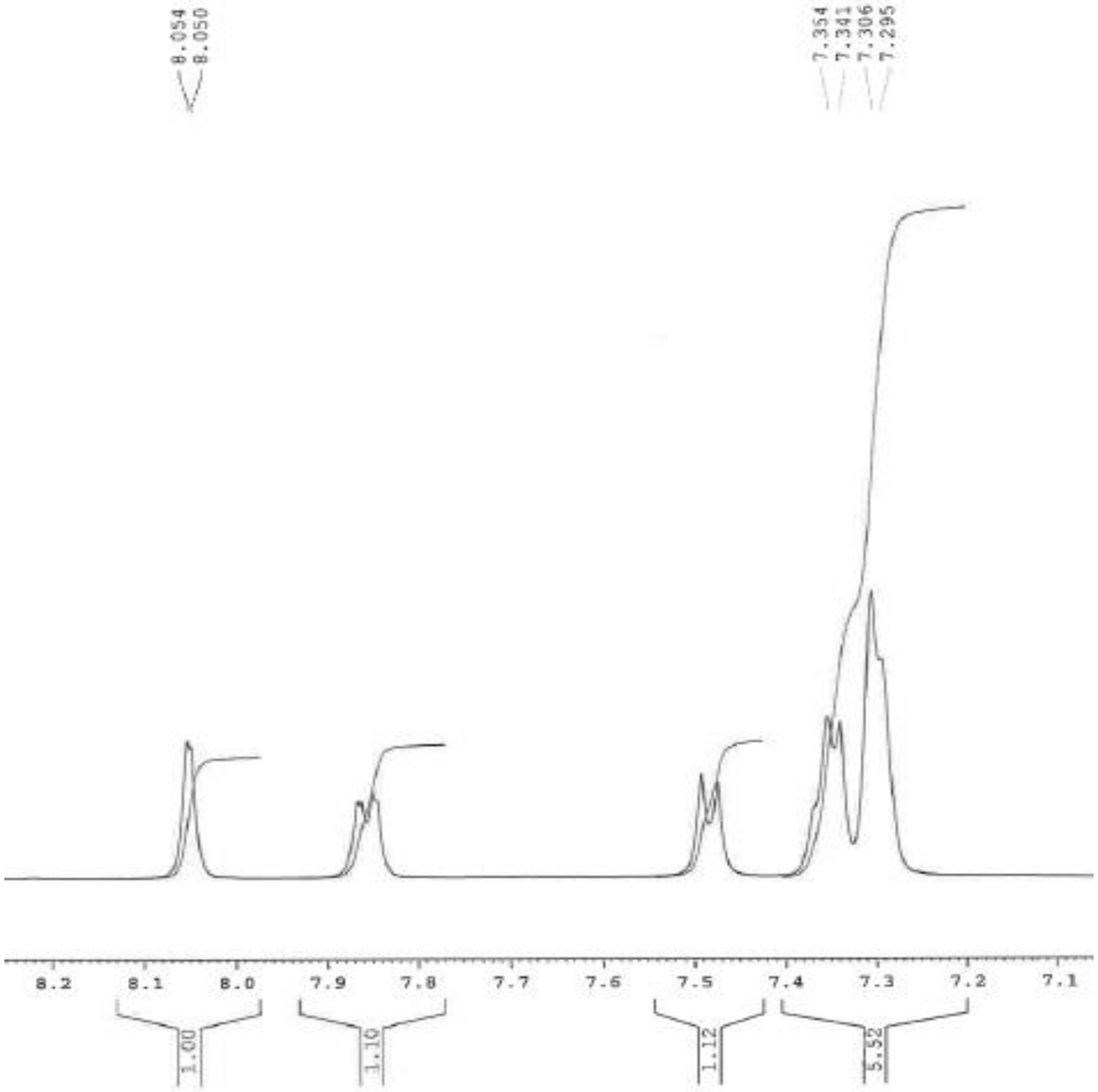

Figure S26. ^{1}HNMR of compound **2f (**extended**)**

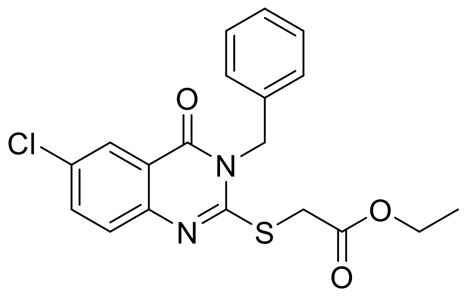

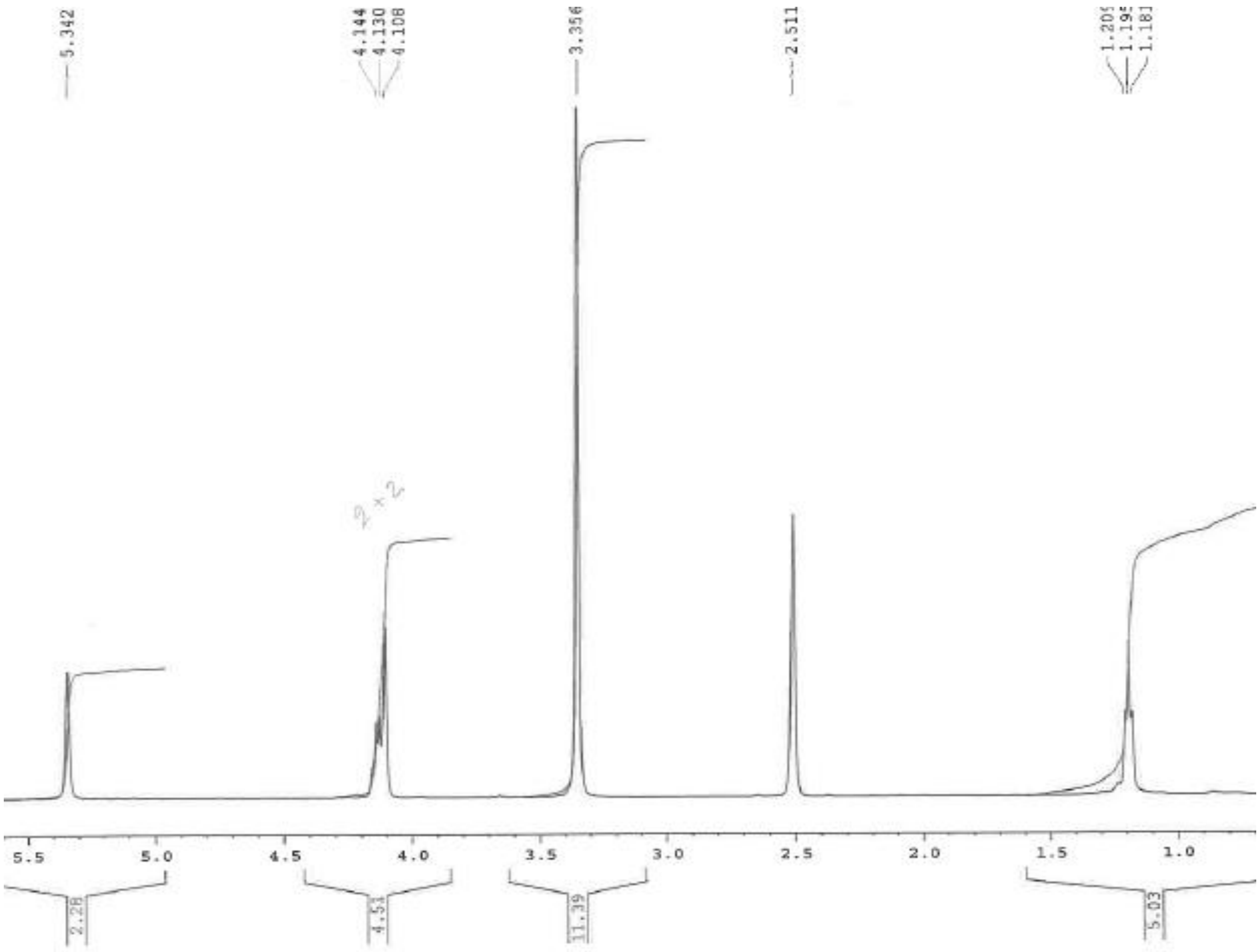

Figure S27. ^{13}CNMR of compound **2f**

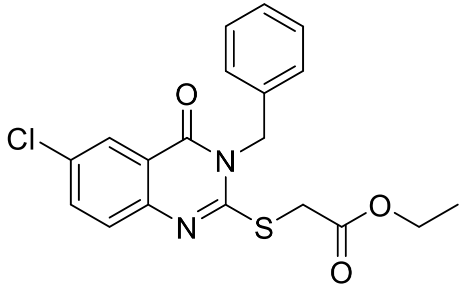

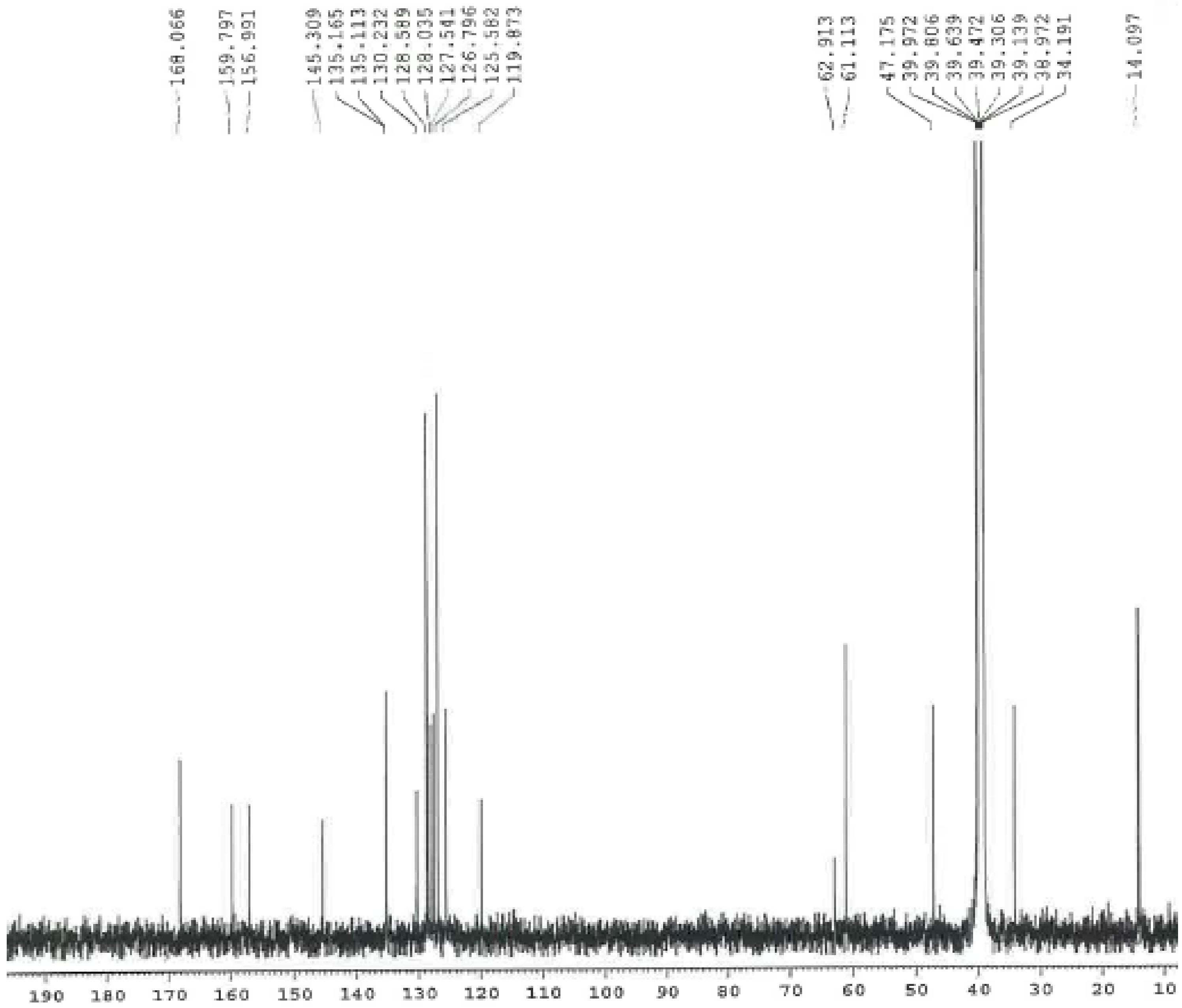

Figure S28. ^{13}CNMR of compound **2f** (extended)

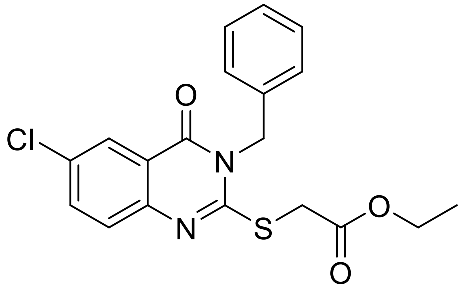

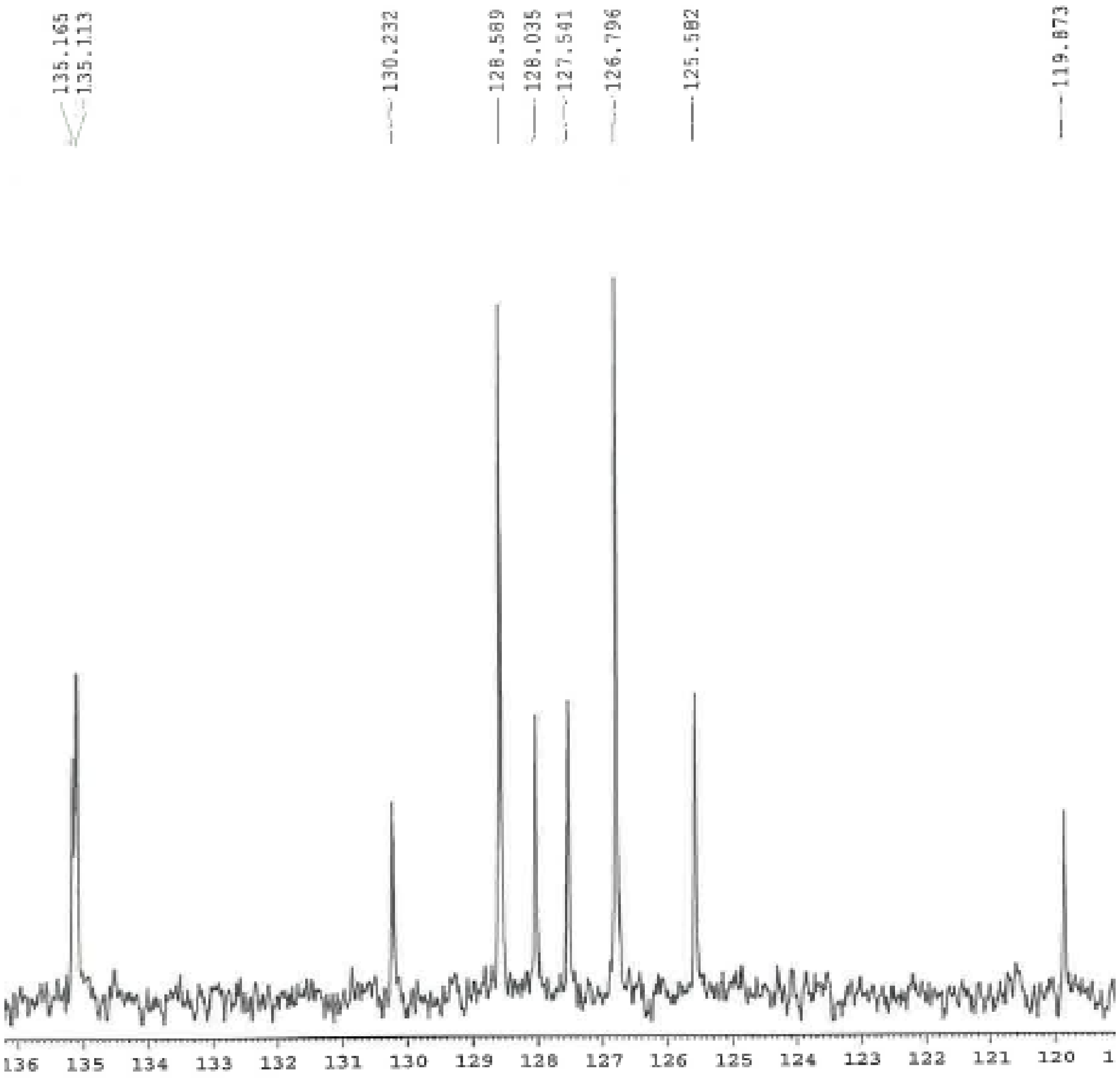

Figure S29. ^{13}CNMR of compound **2f** (extended)

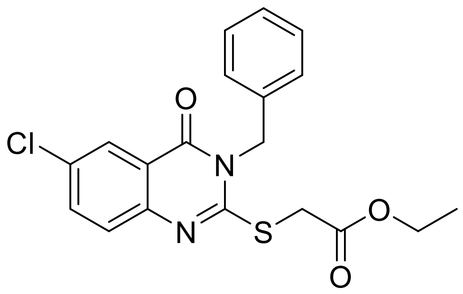

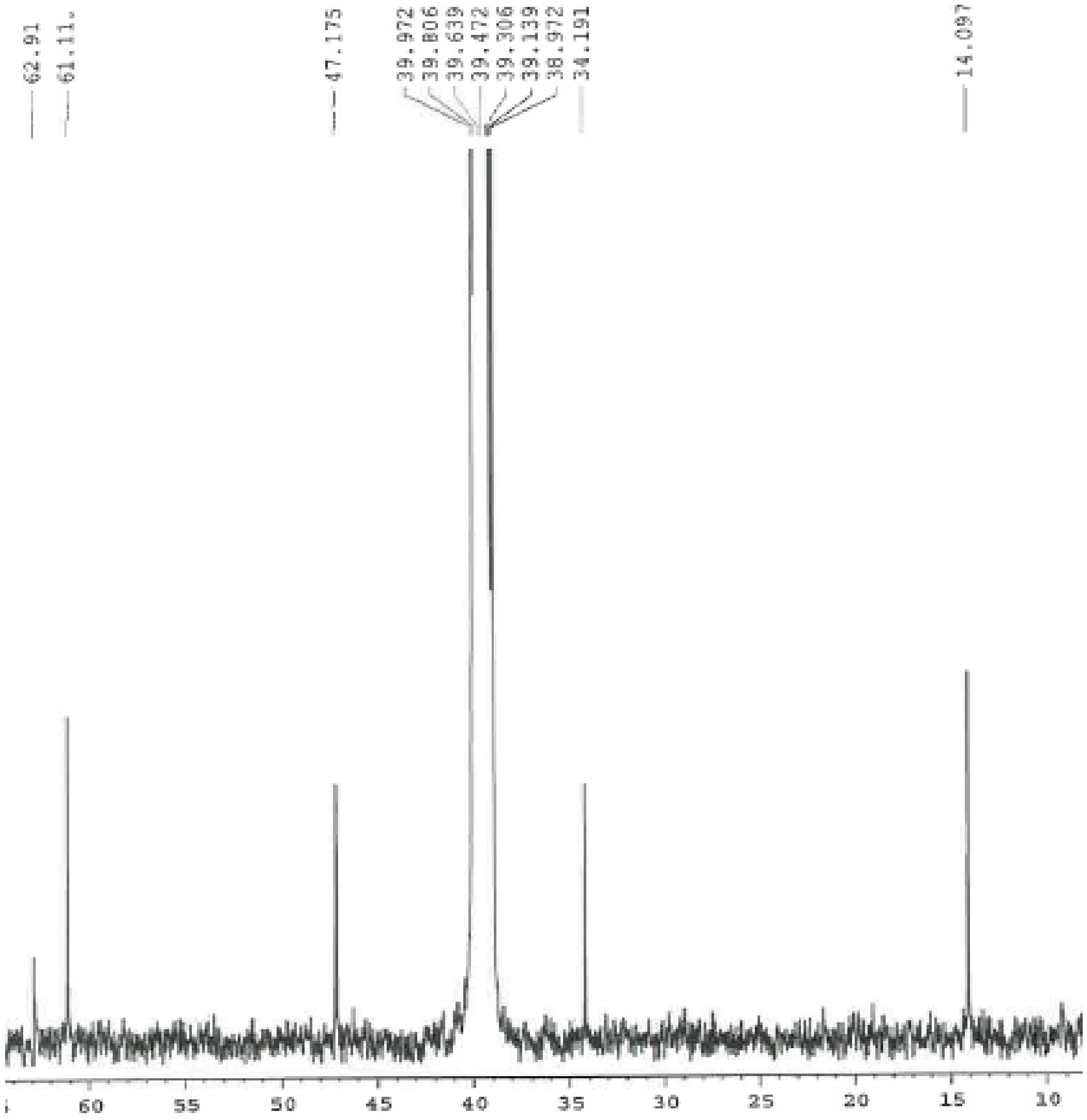

Figure S30. ^{1}HNMR of compound **2h**

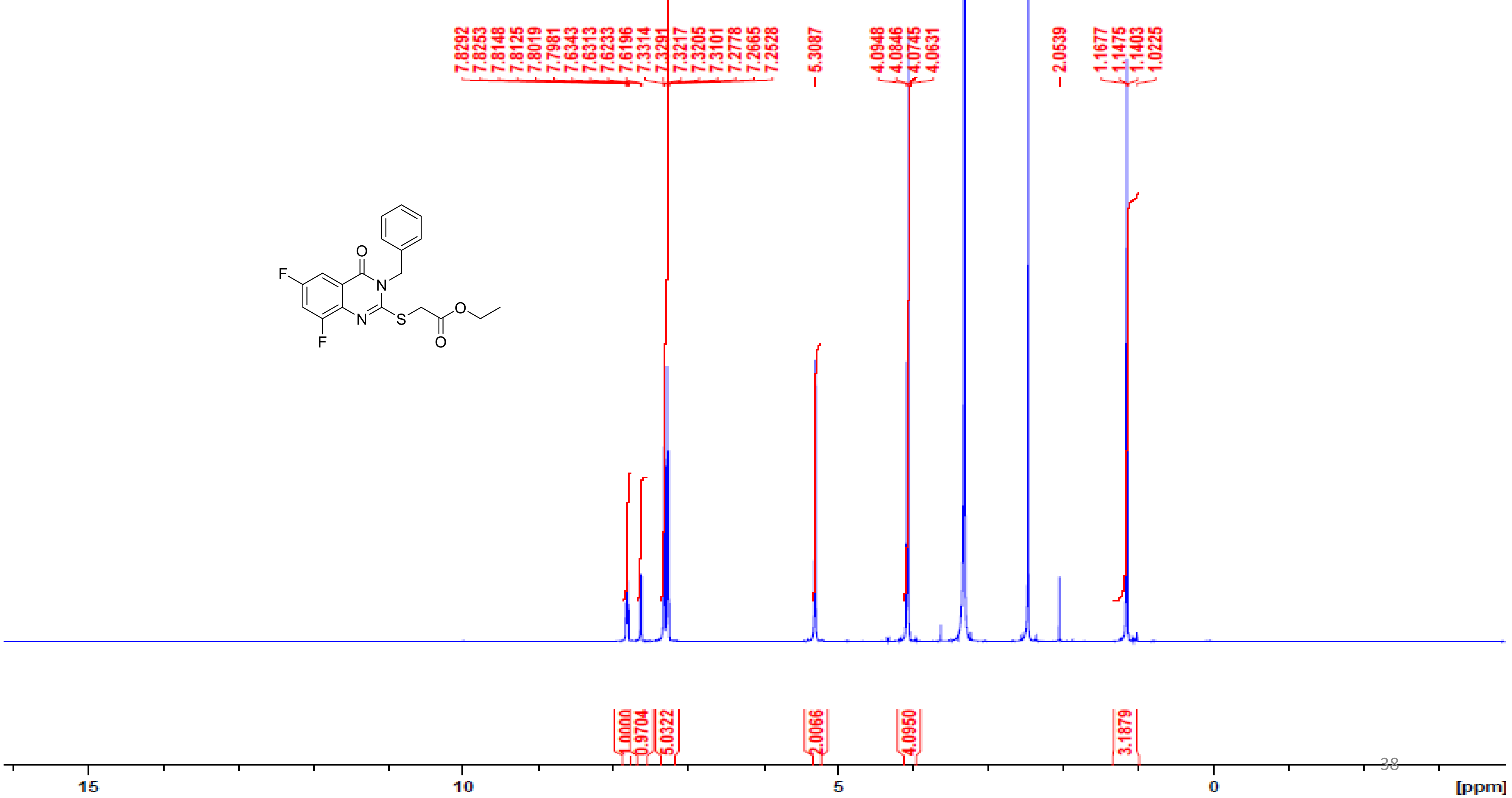

Figure S31. ^{1}HNMR of compound **2h (**extended**)**

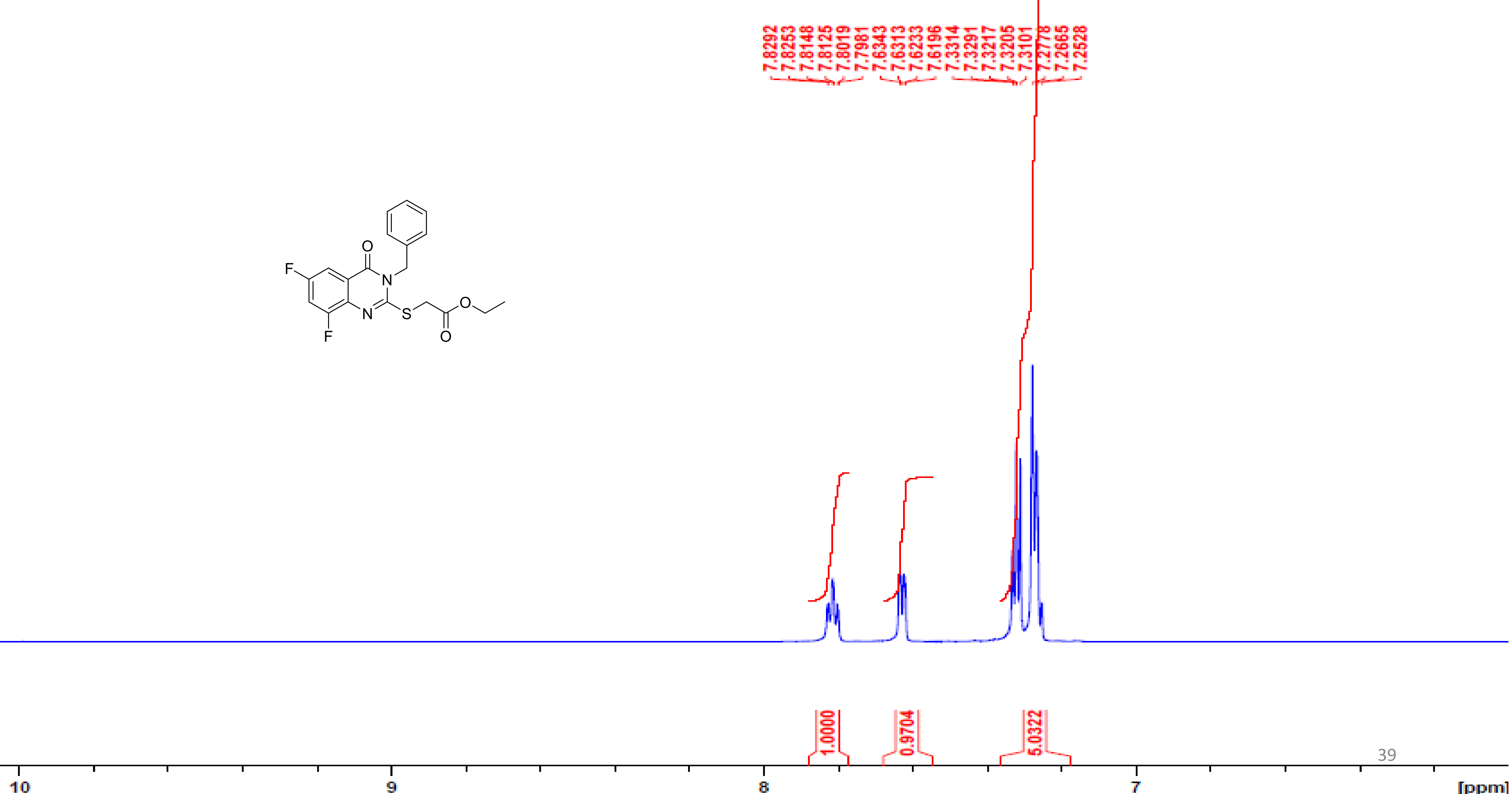

Figure S32. ^{13}CNMR of compound **2h**

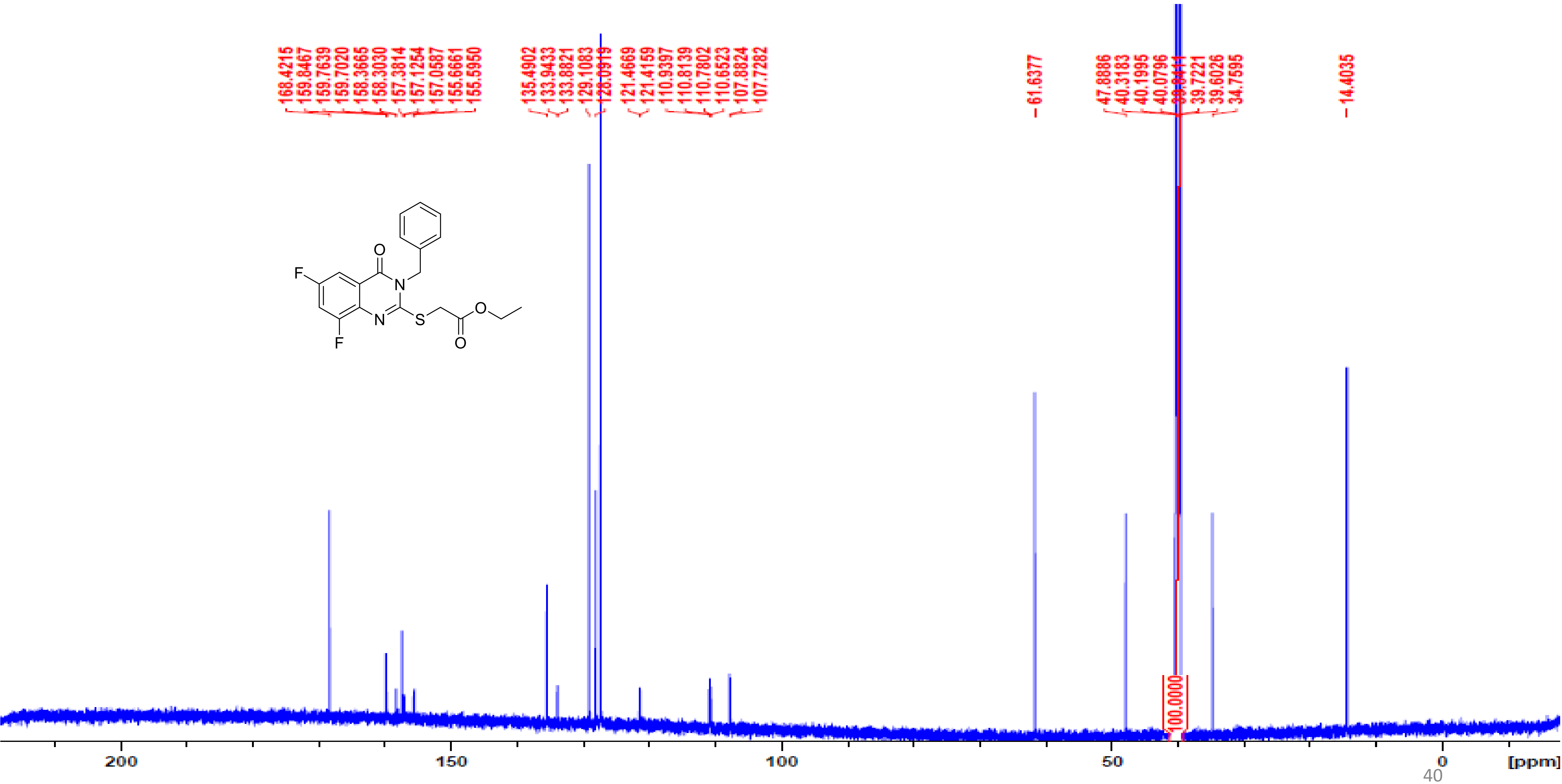

Figure S33. ^{13}CNMR of compound **2h** (extended)

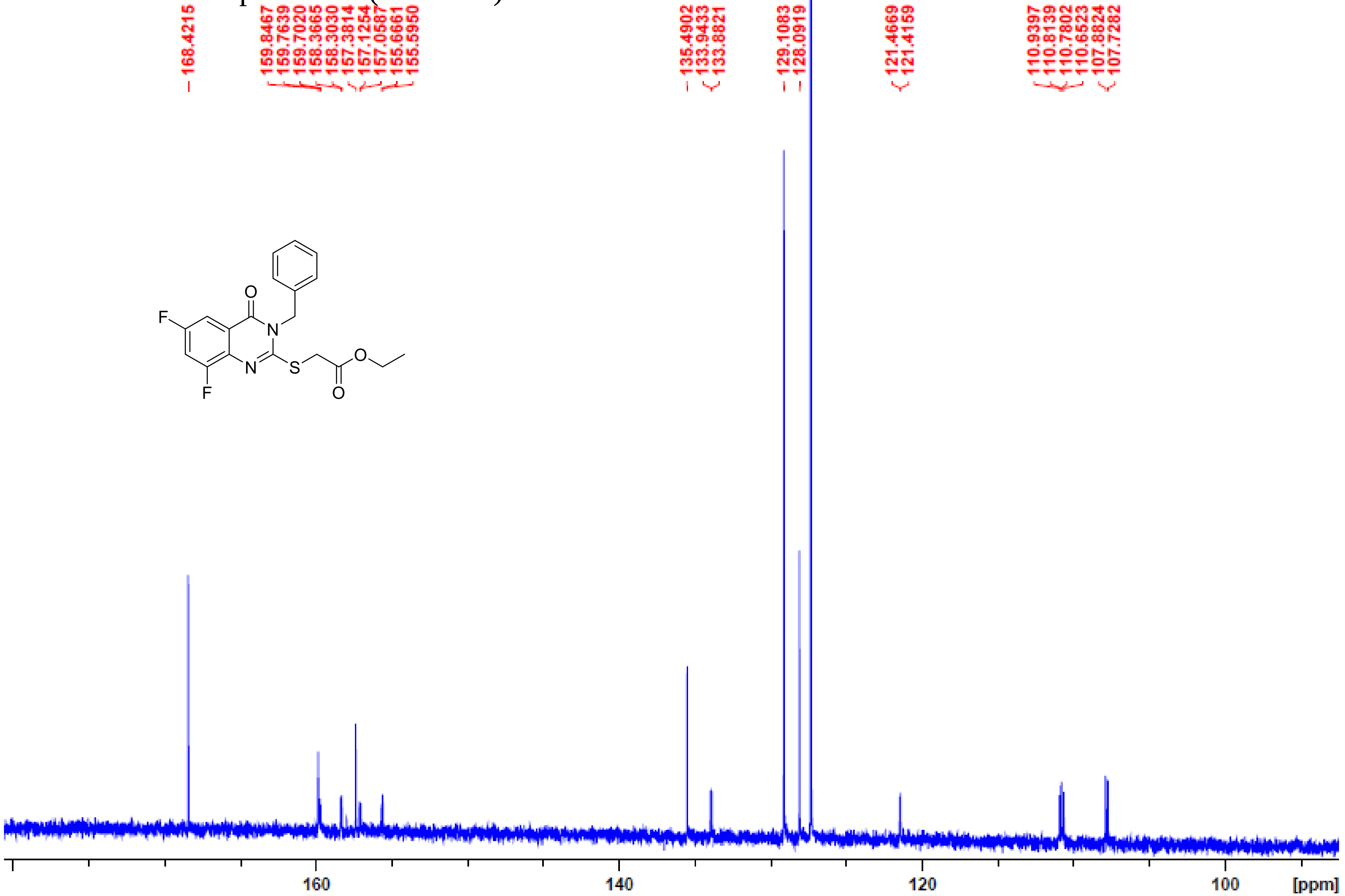

Figure S34. ^{1}HNMR of compound **2j**

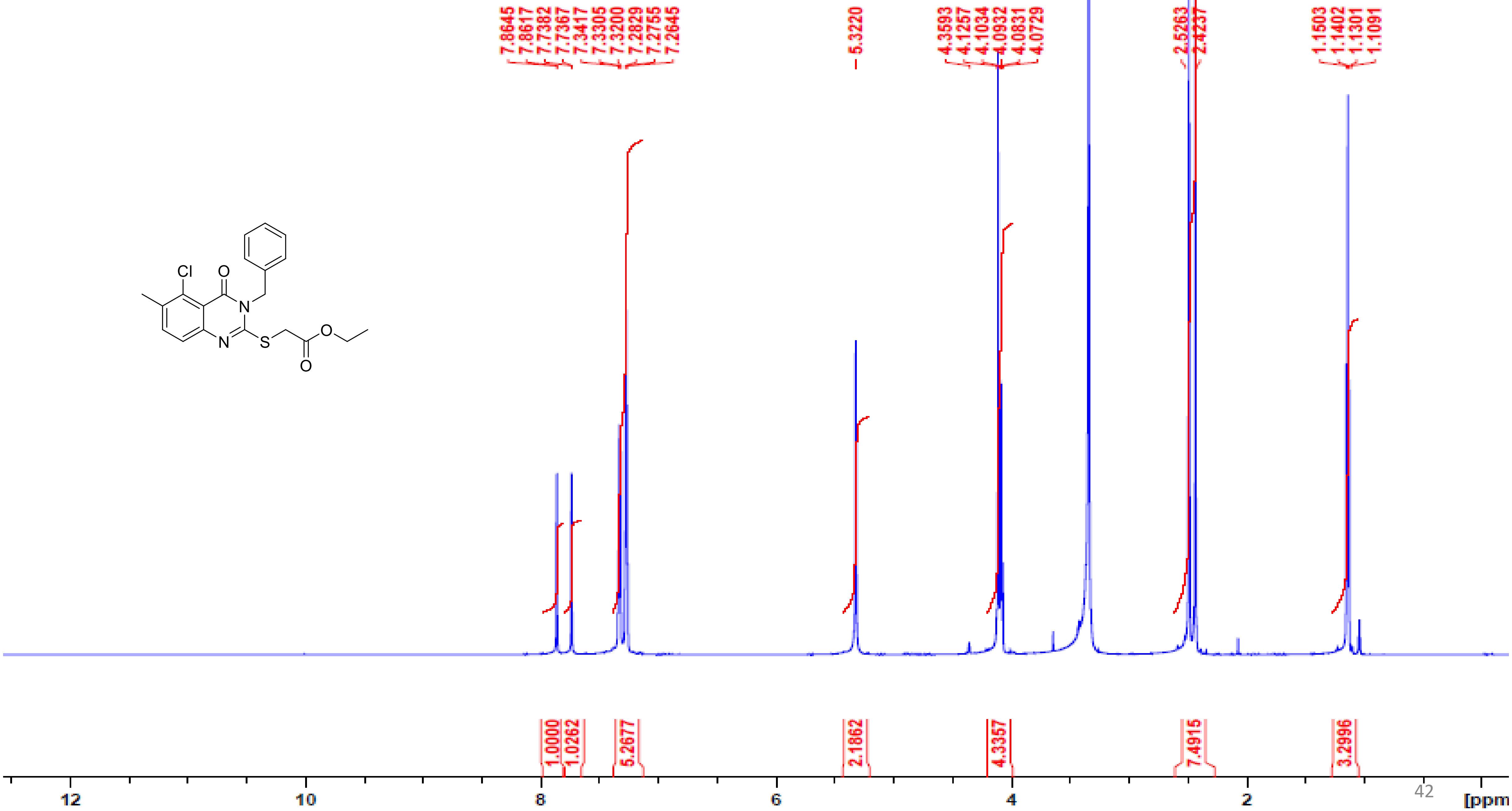

Figure S35. ^{1}HNMR of compound **2j** (extended)

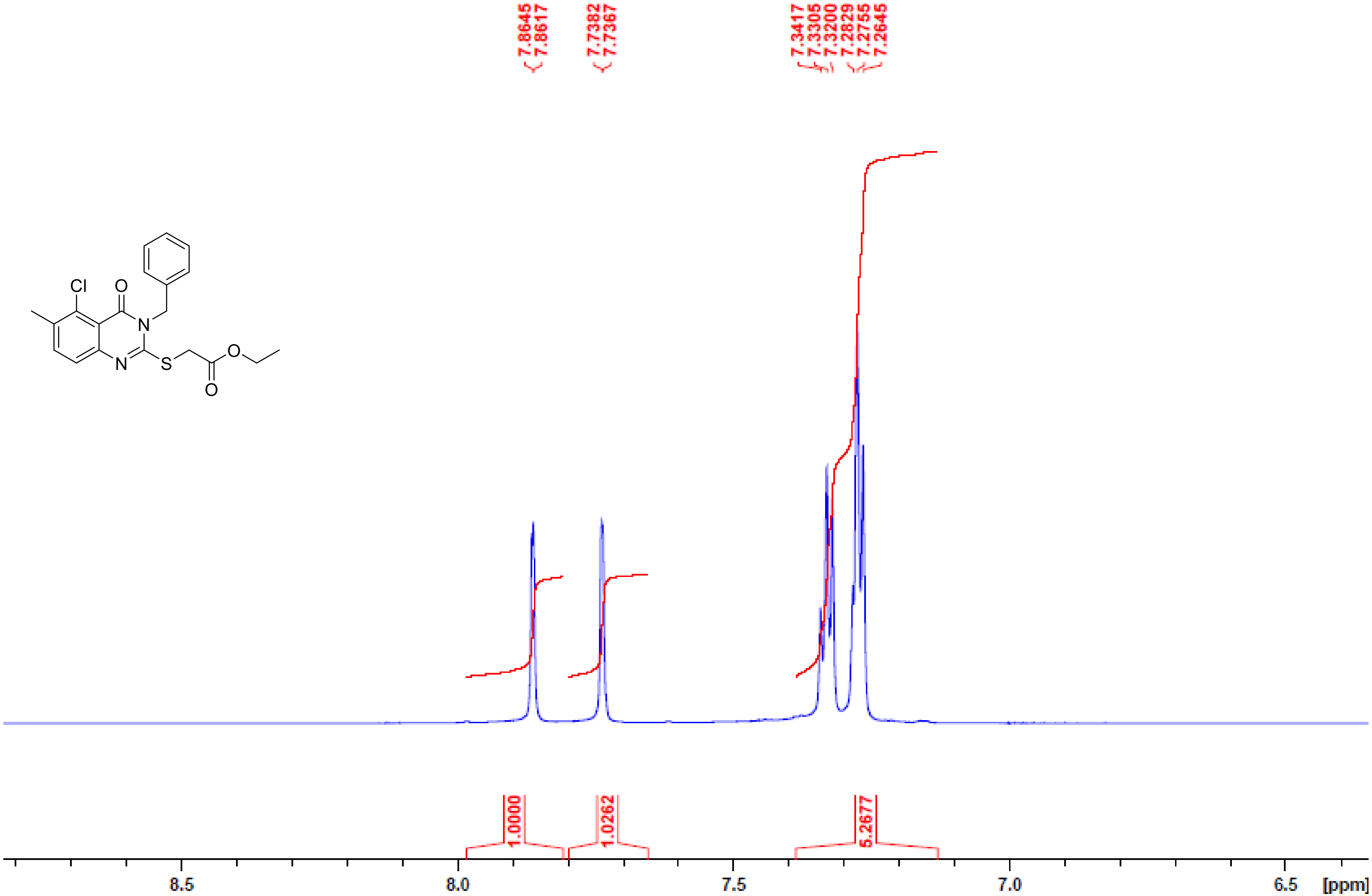

Figure S36. ^{13}CNMR of compound **2j**

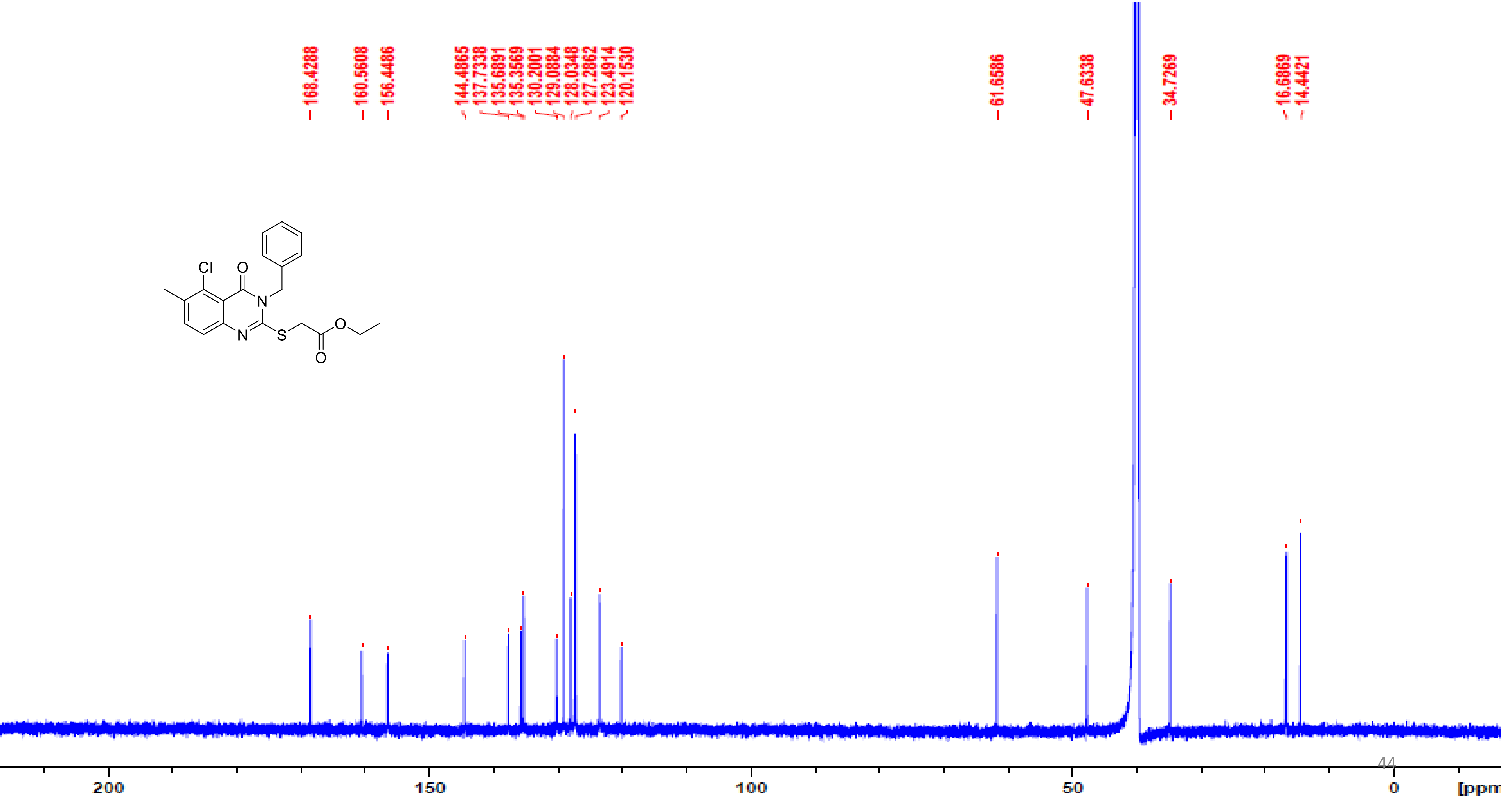

Figure S37. ^{13}CNMR of compound **2j** (extended)

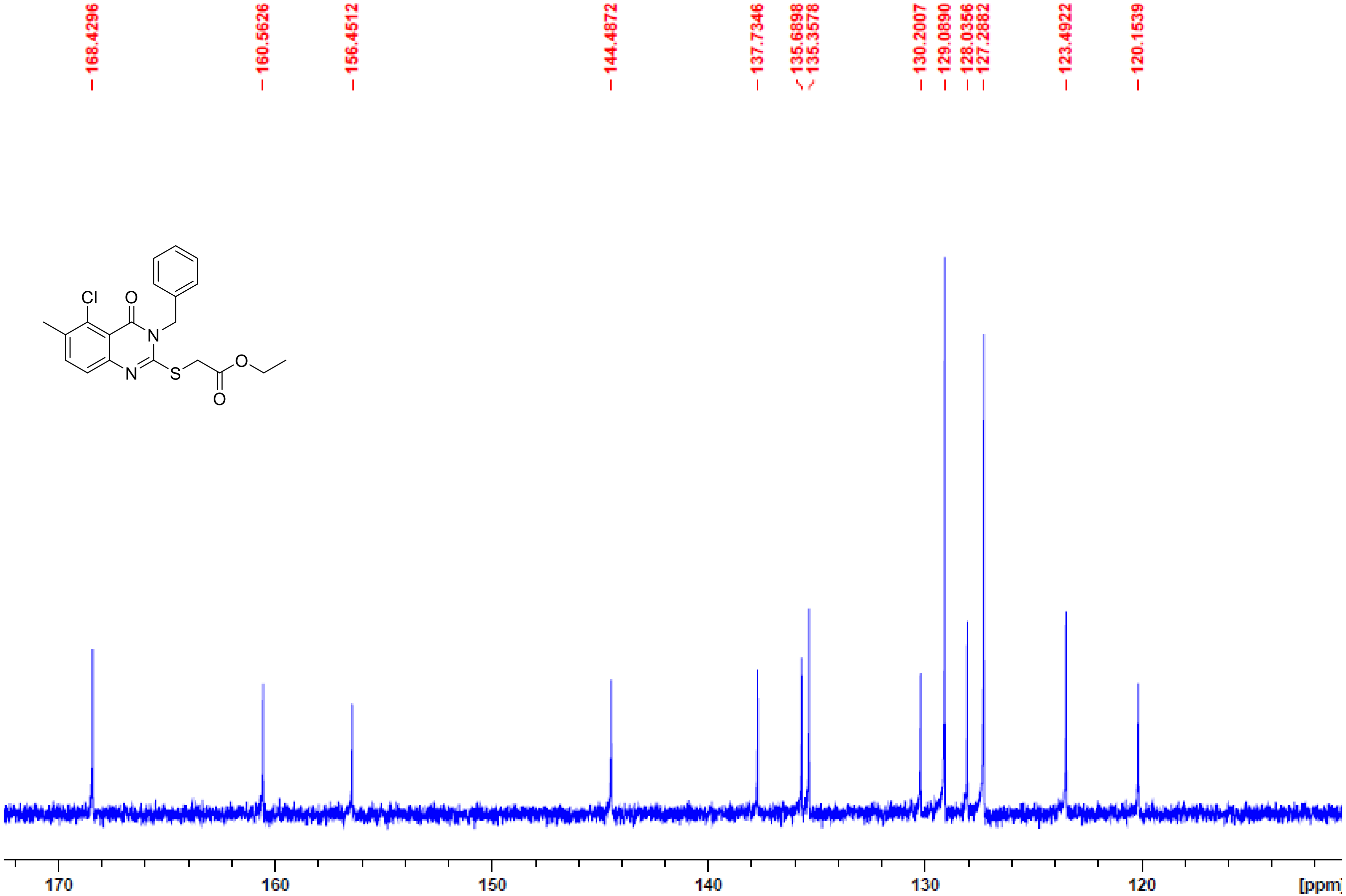

Figure S38. ^{1}HNMR of compound **3a**

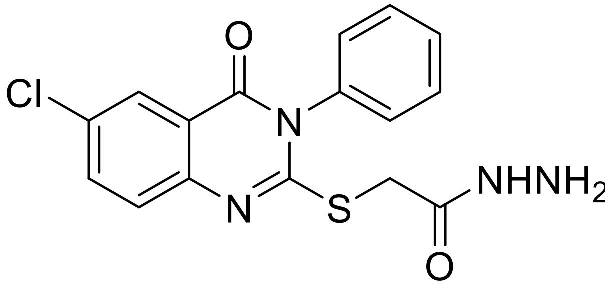

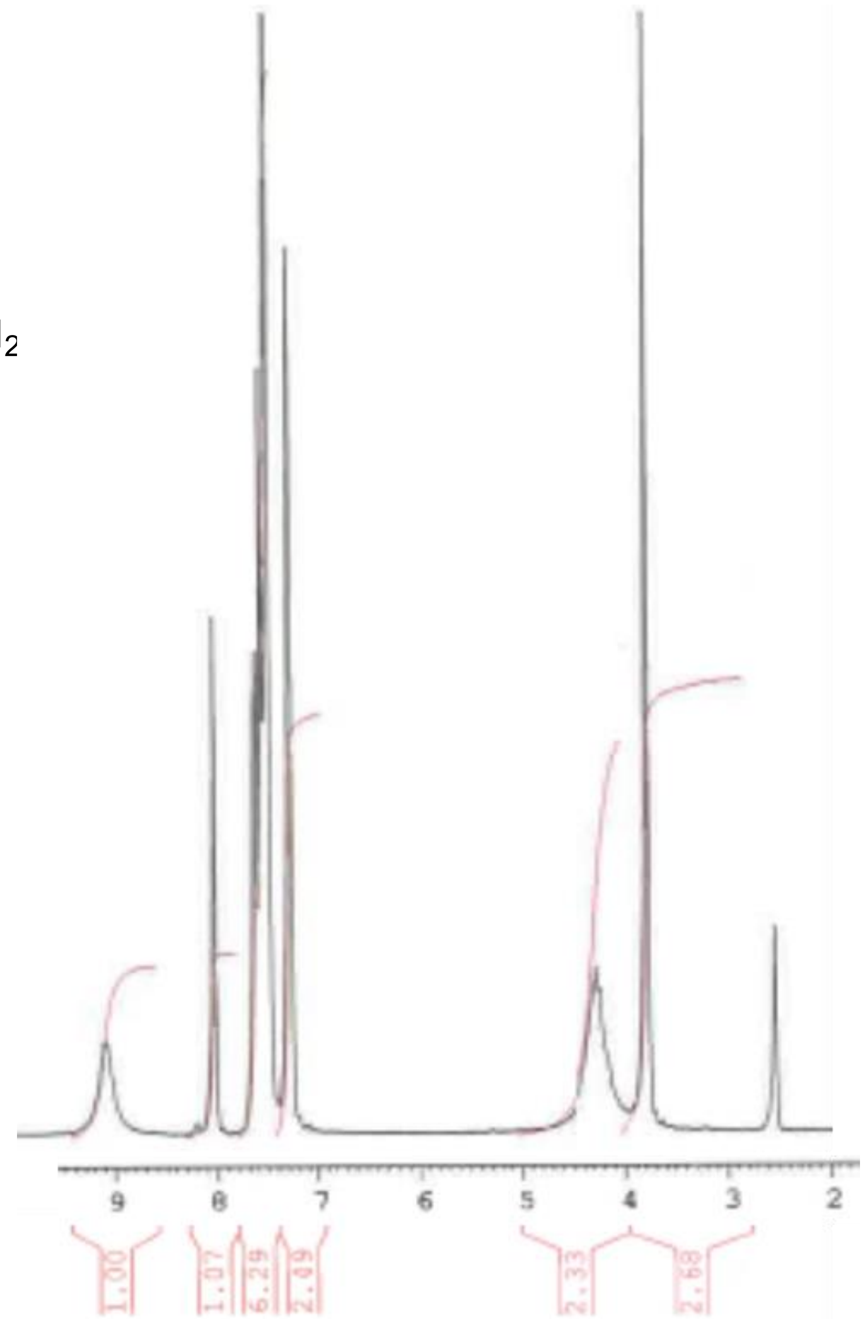

Figure S39. ^{1}HNMR of compound **3a (**extended**)**

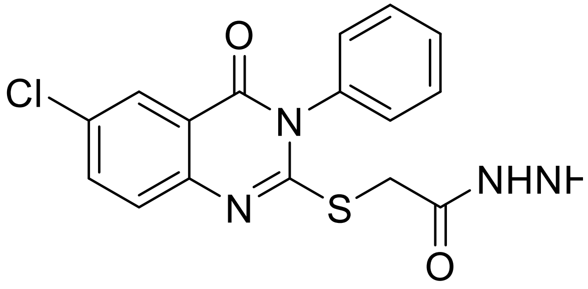

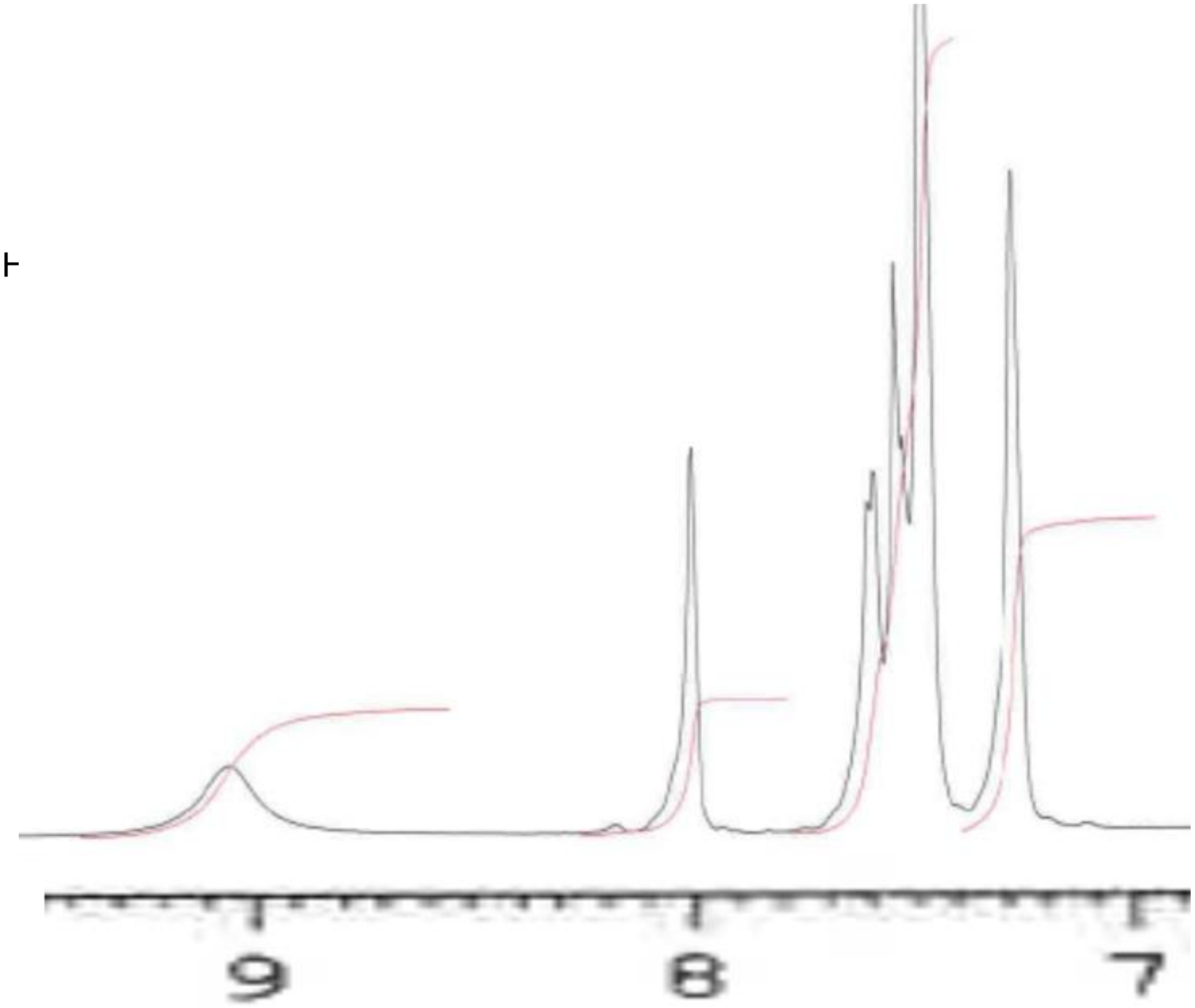

Figure S40. ^{13}CNMR of compound **3a**

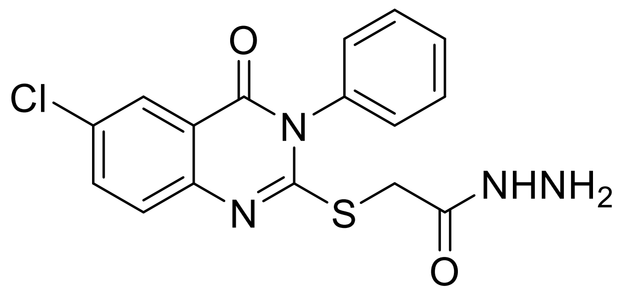

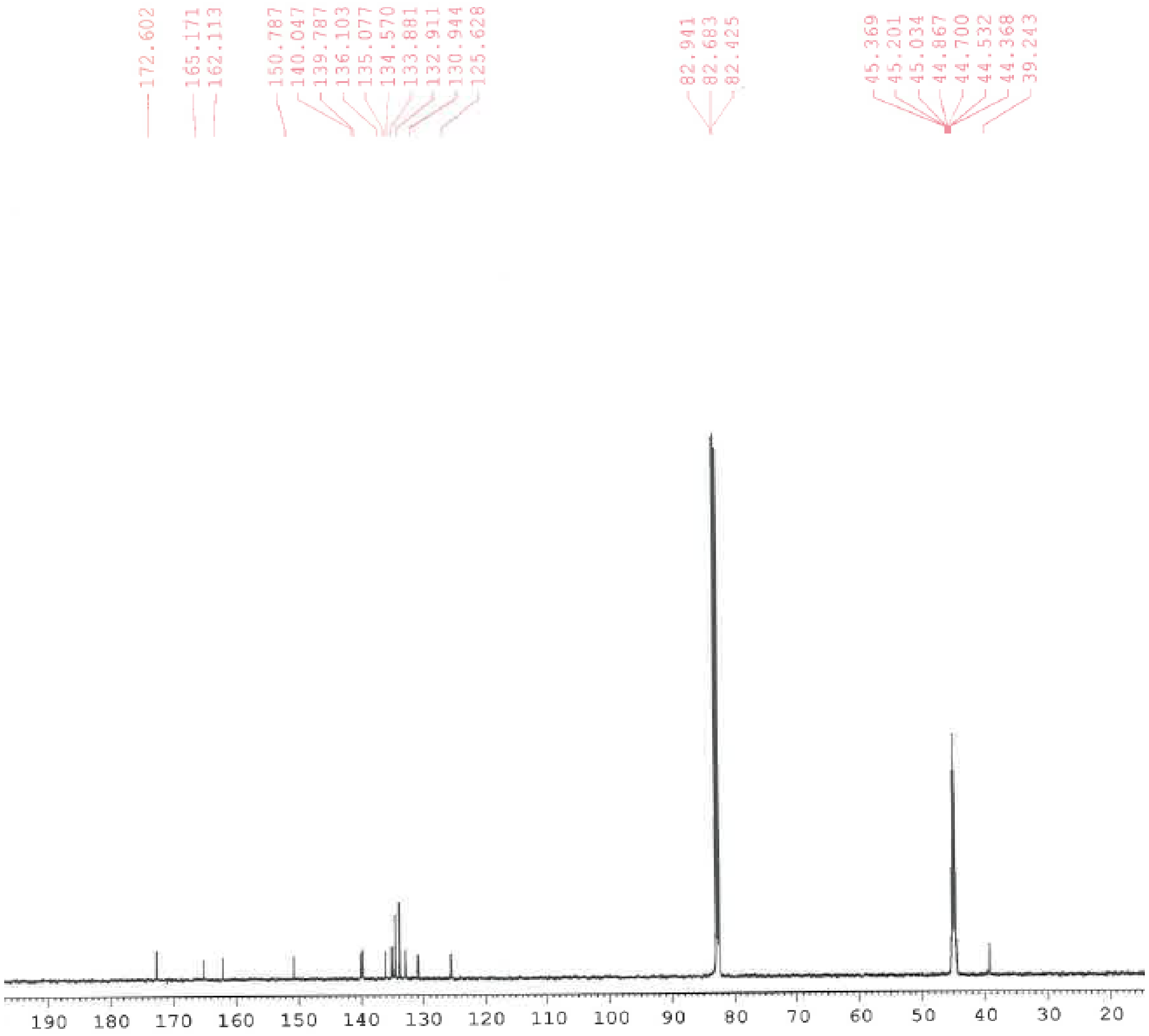

Figure S41. ^{13}CNMR of compound **3a** (extended)

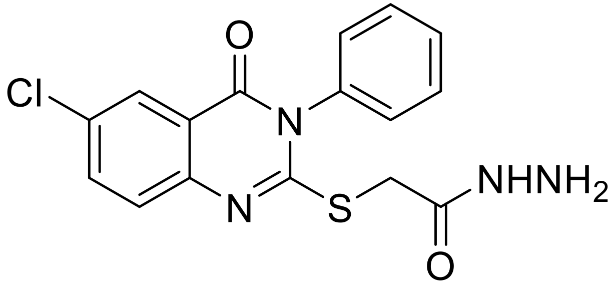

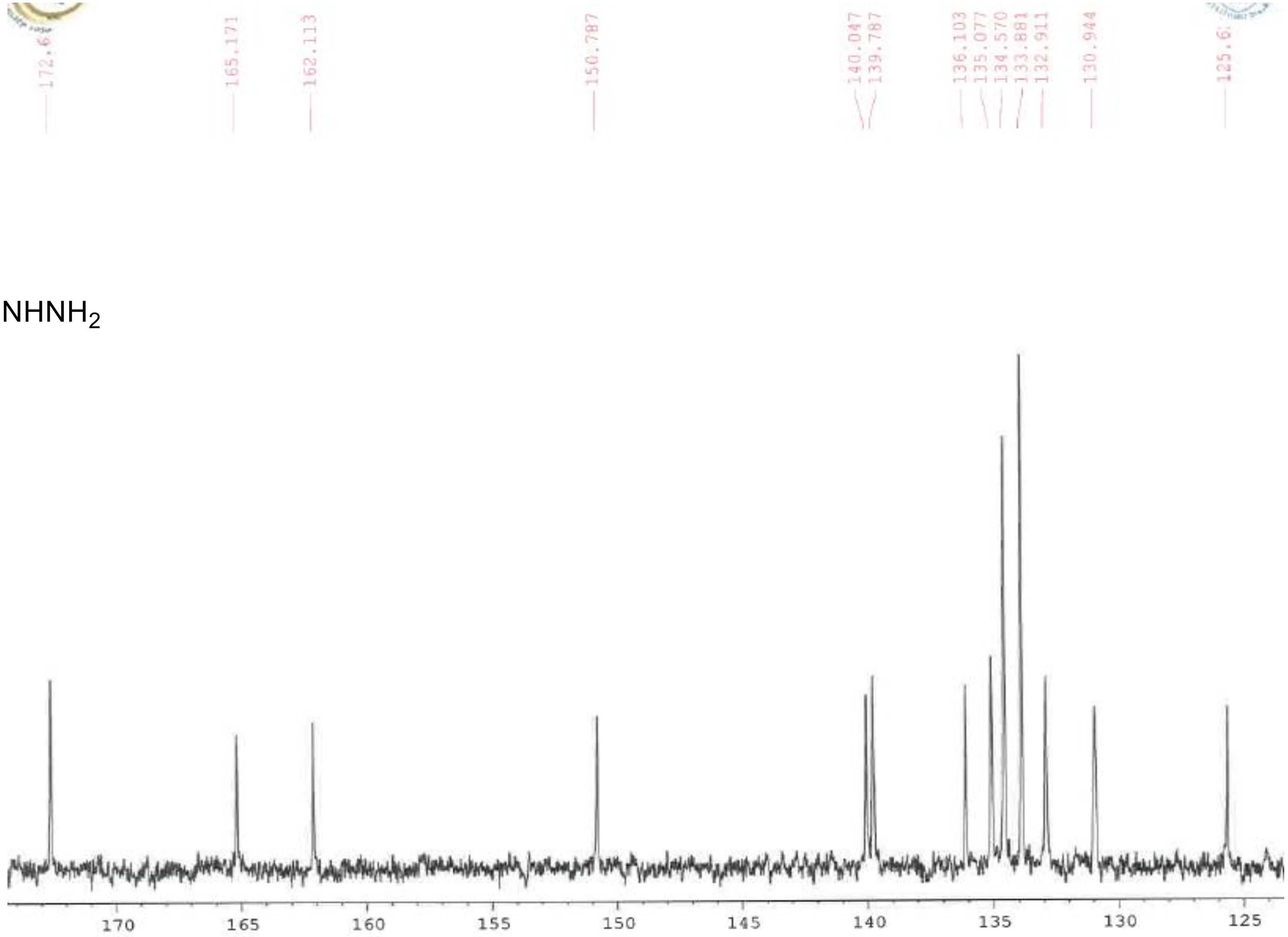

Figure S42. ^{1}HNMR of compound **3b**

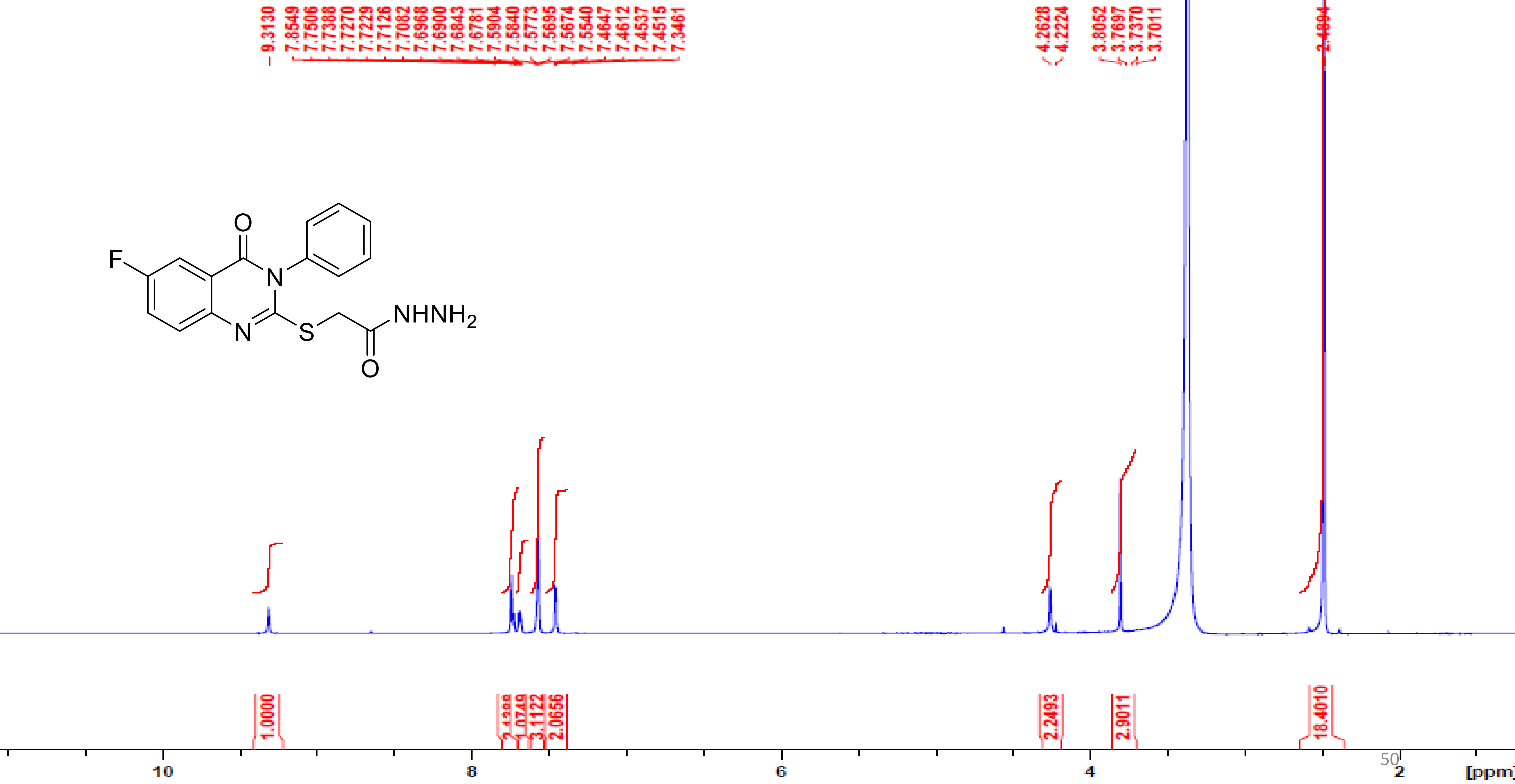

Figure S43. ^{1}HNMR of compound **3b (**extended**)**

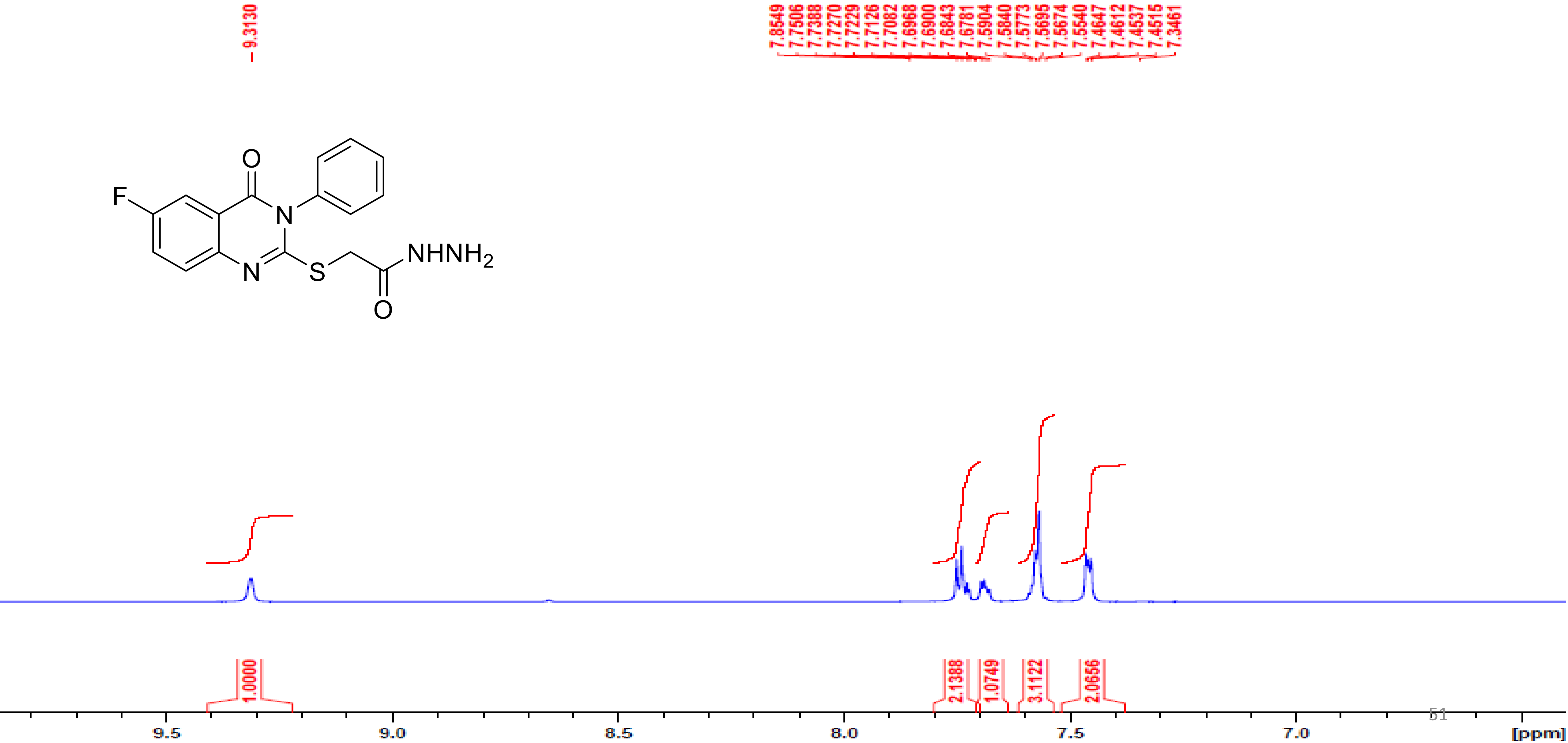

Figure S44. ^{13}CNMR of compound **3b**

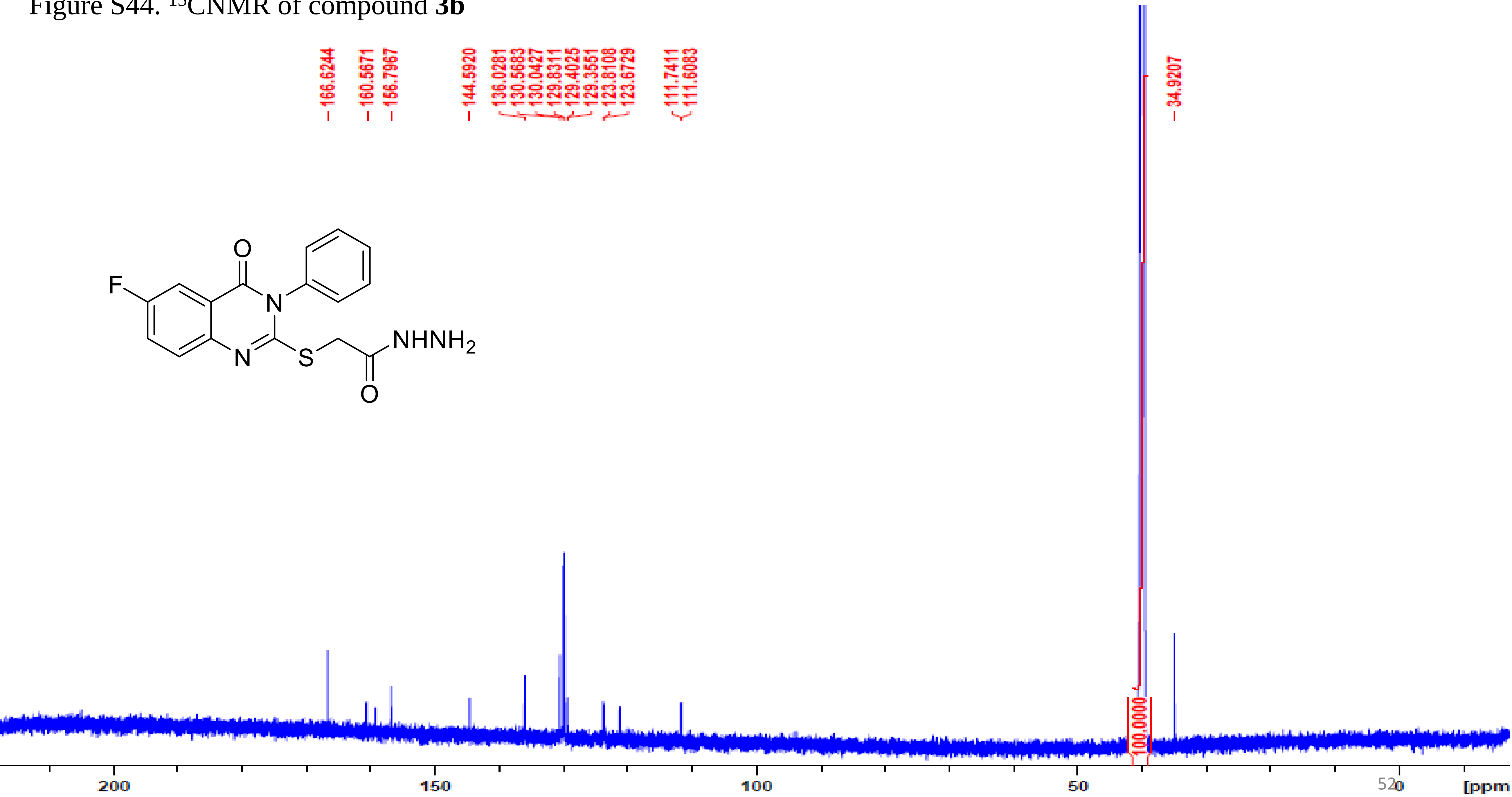

Figure S45. ^{13}CNMR of compound **3b** (extended)

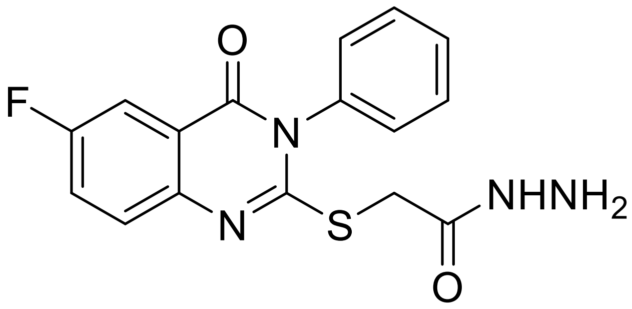

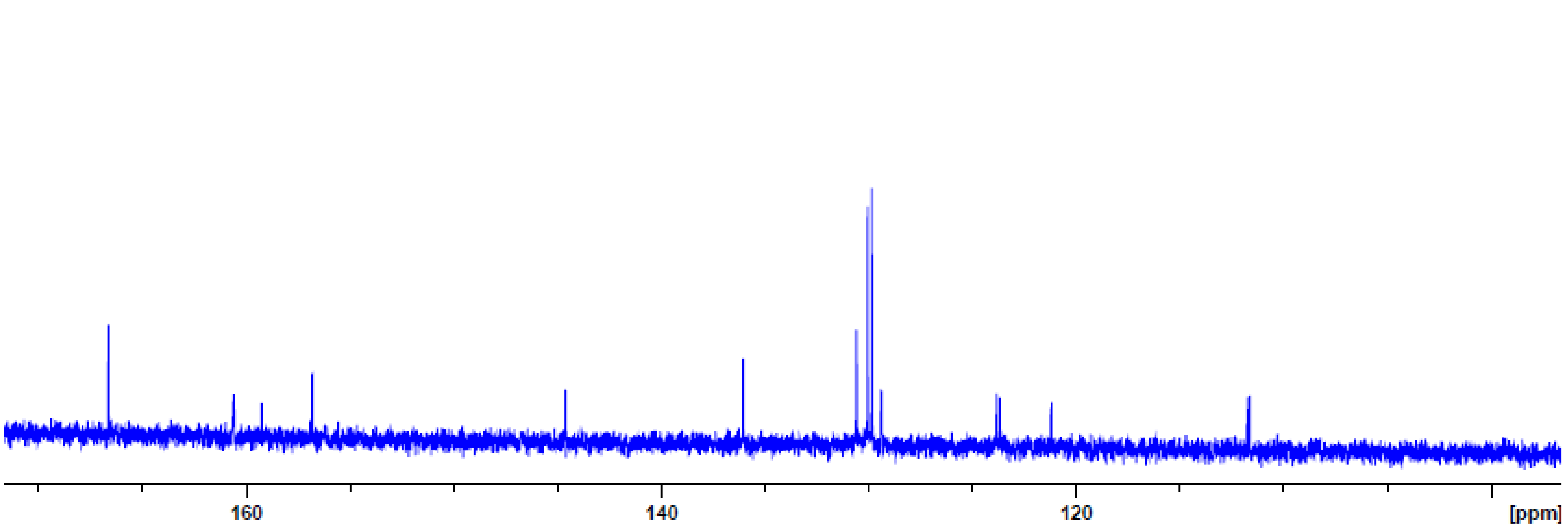

Figure S46. ^{1}HNMR of compound **3c**

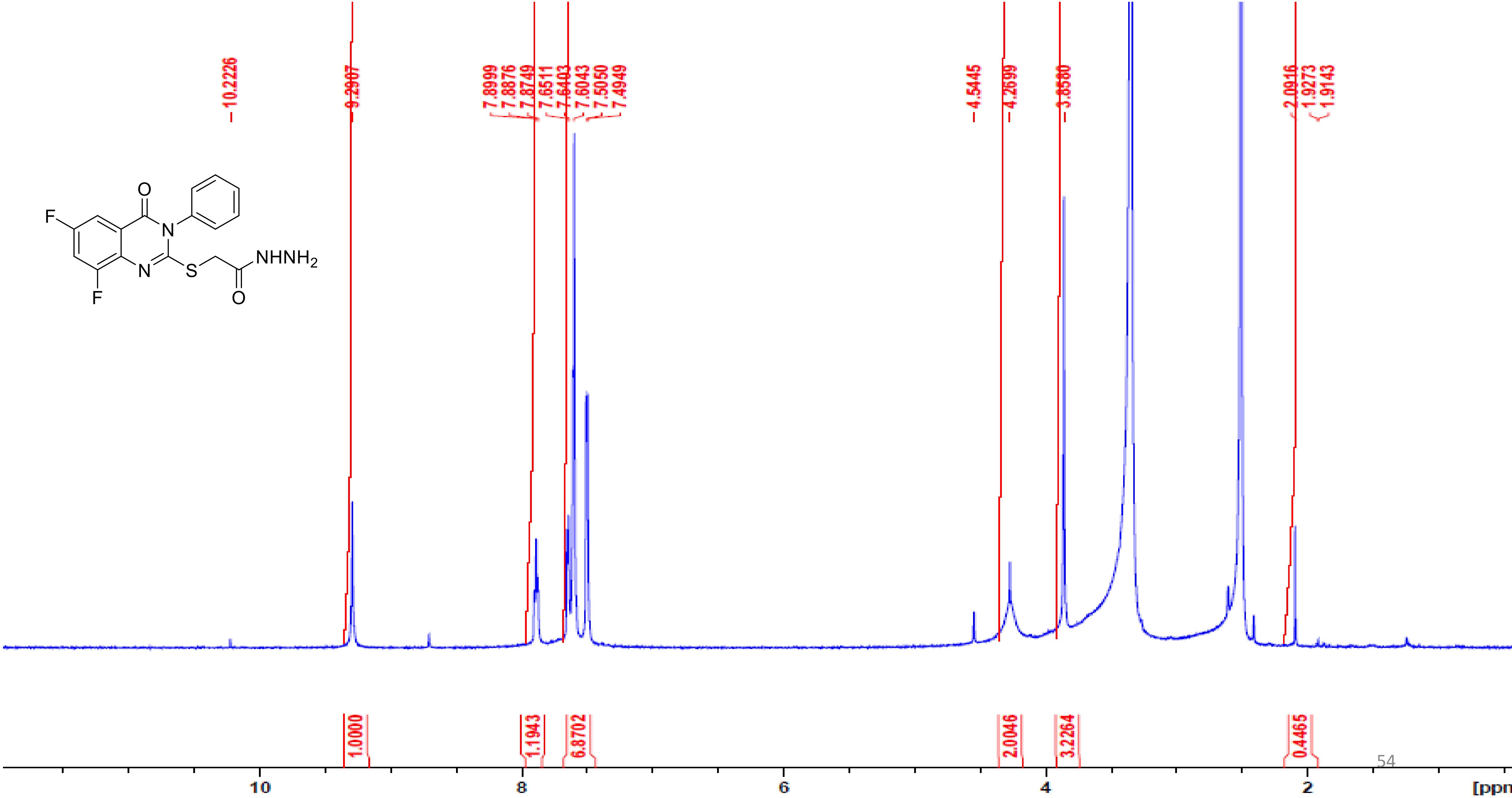

Figure S47. ^{1}HNMR of compound **3c (**extended**)**

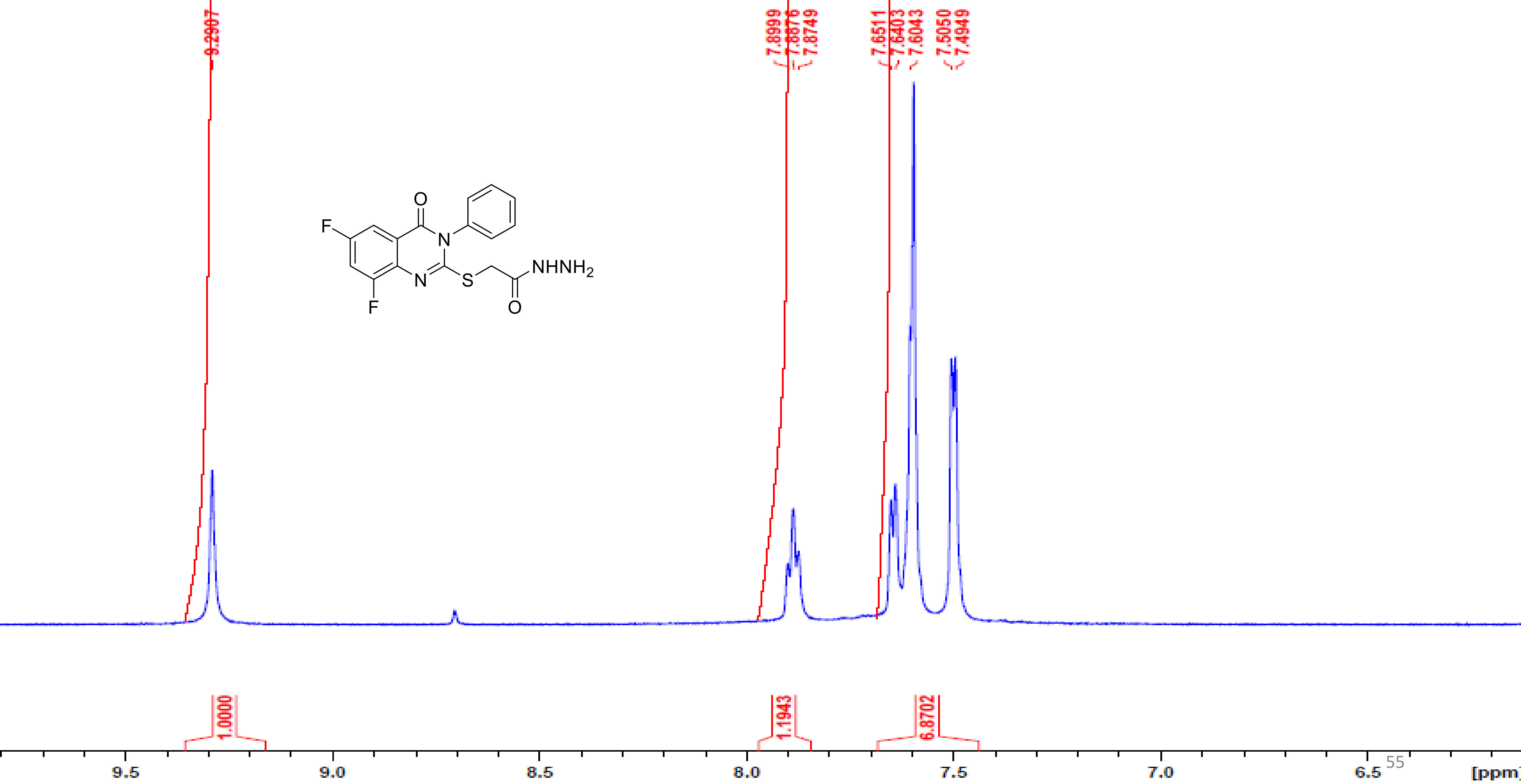

Figure S48. ^{1}HNMR of compound **3d**

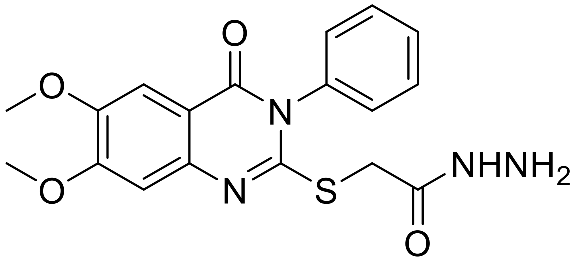

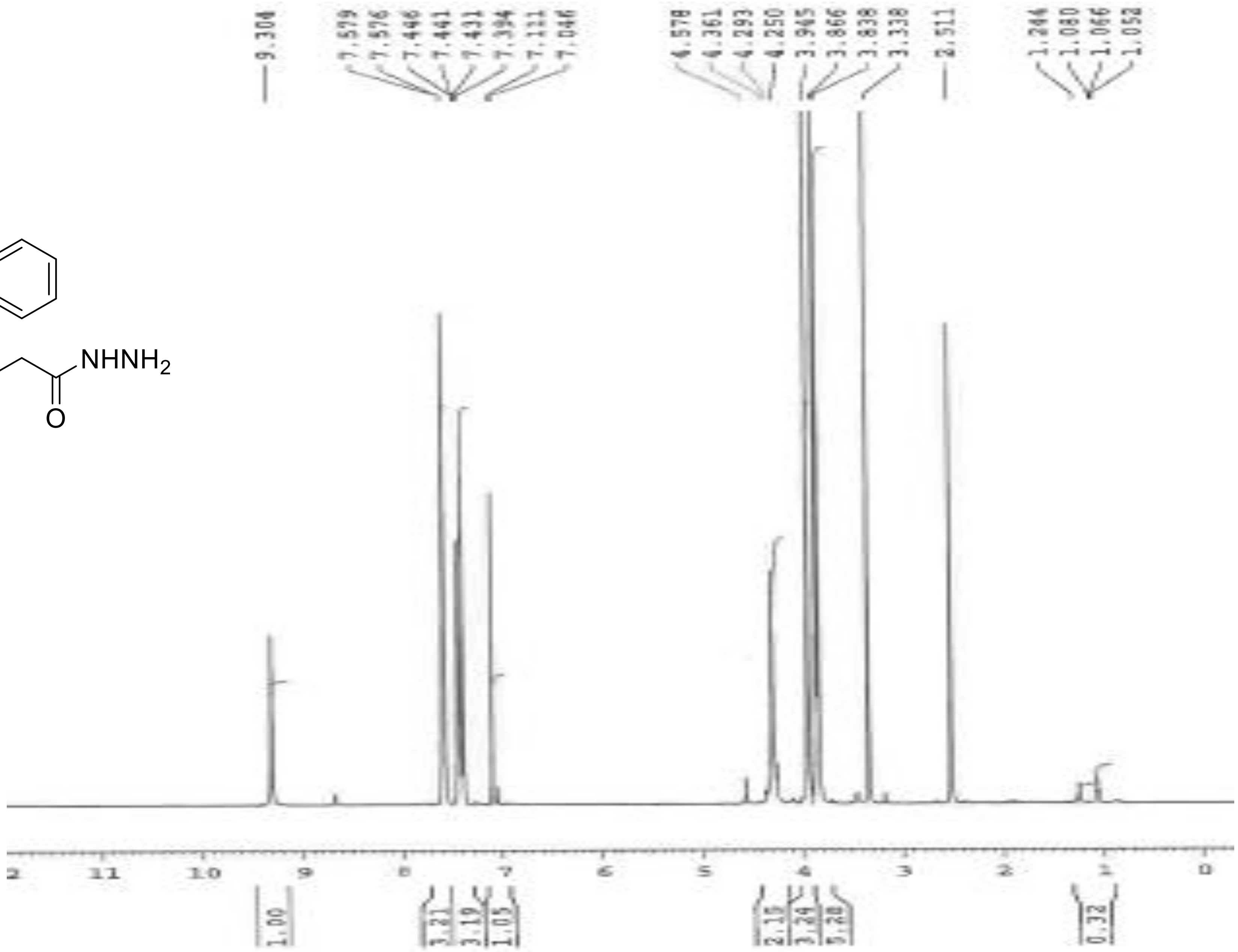

Figure S49. ^{1}HNMR of compound **3d (**extended**)**

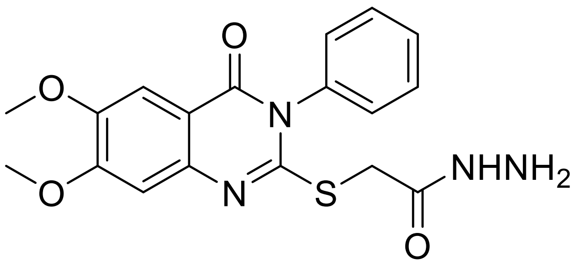

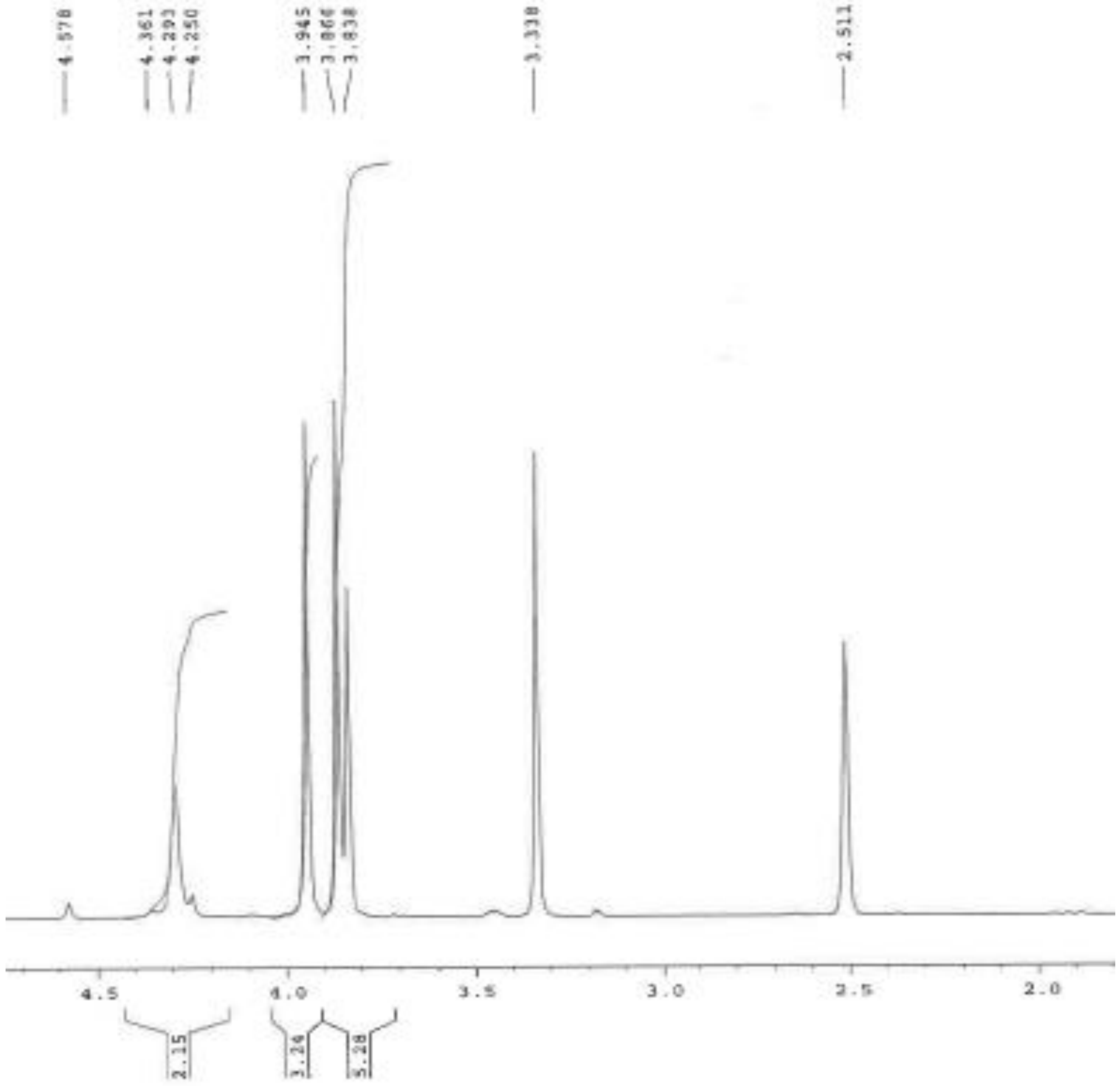

Figure S50. ^{1}HNMR of compound **3d (**extended**)**

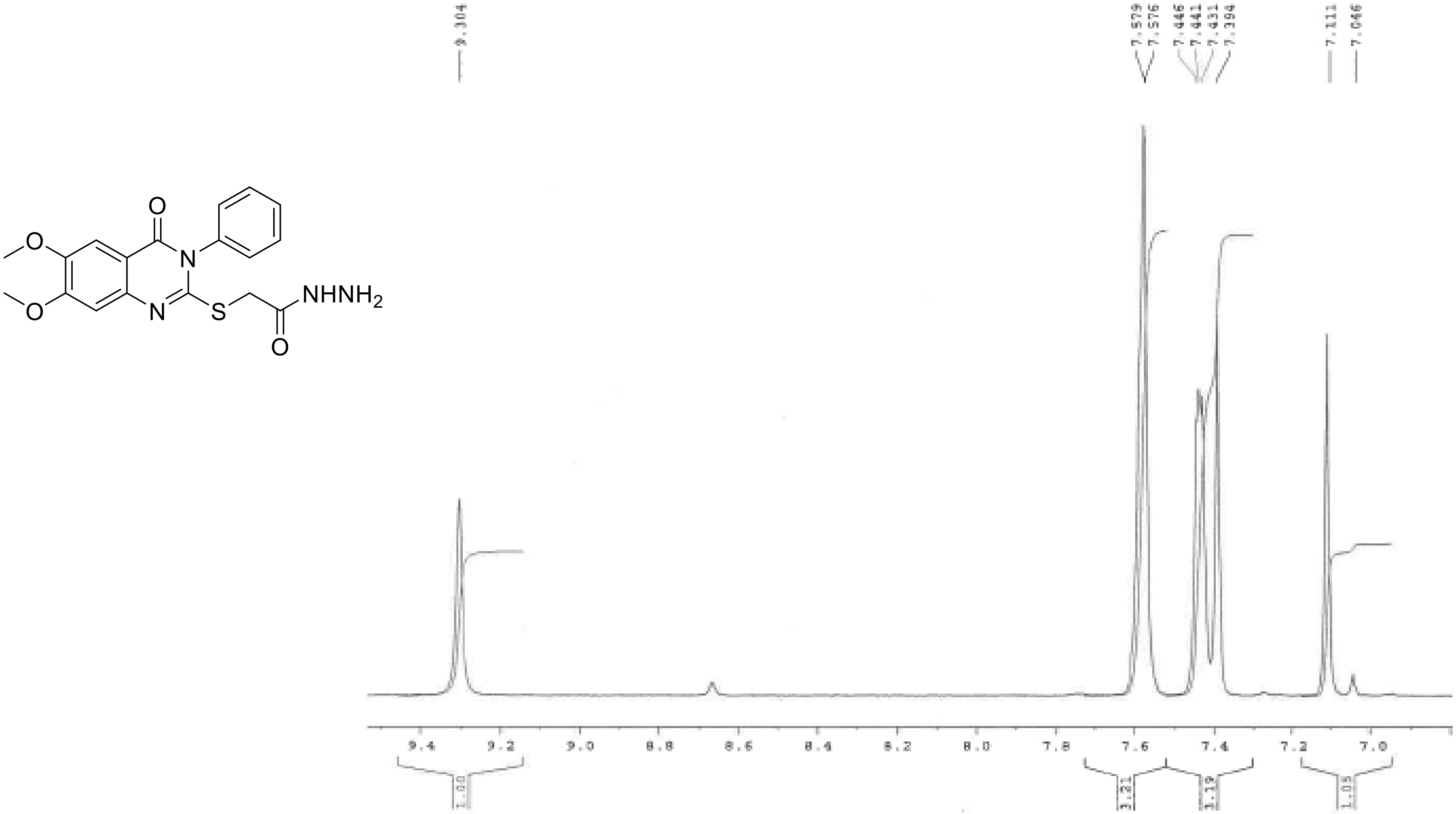

Figure S51. ^{13}CNMR of compound **3d**

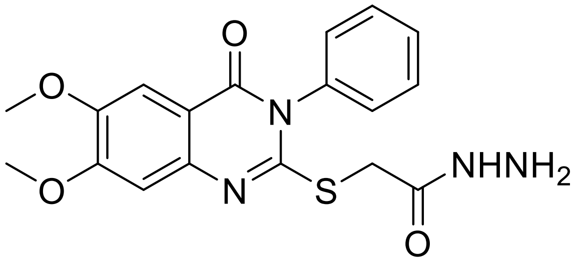

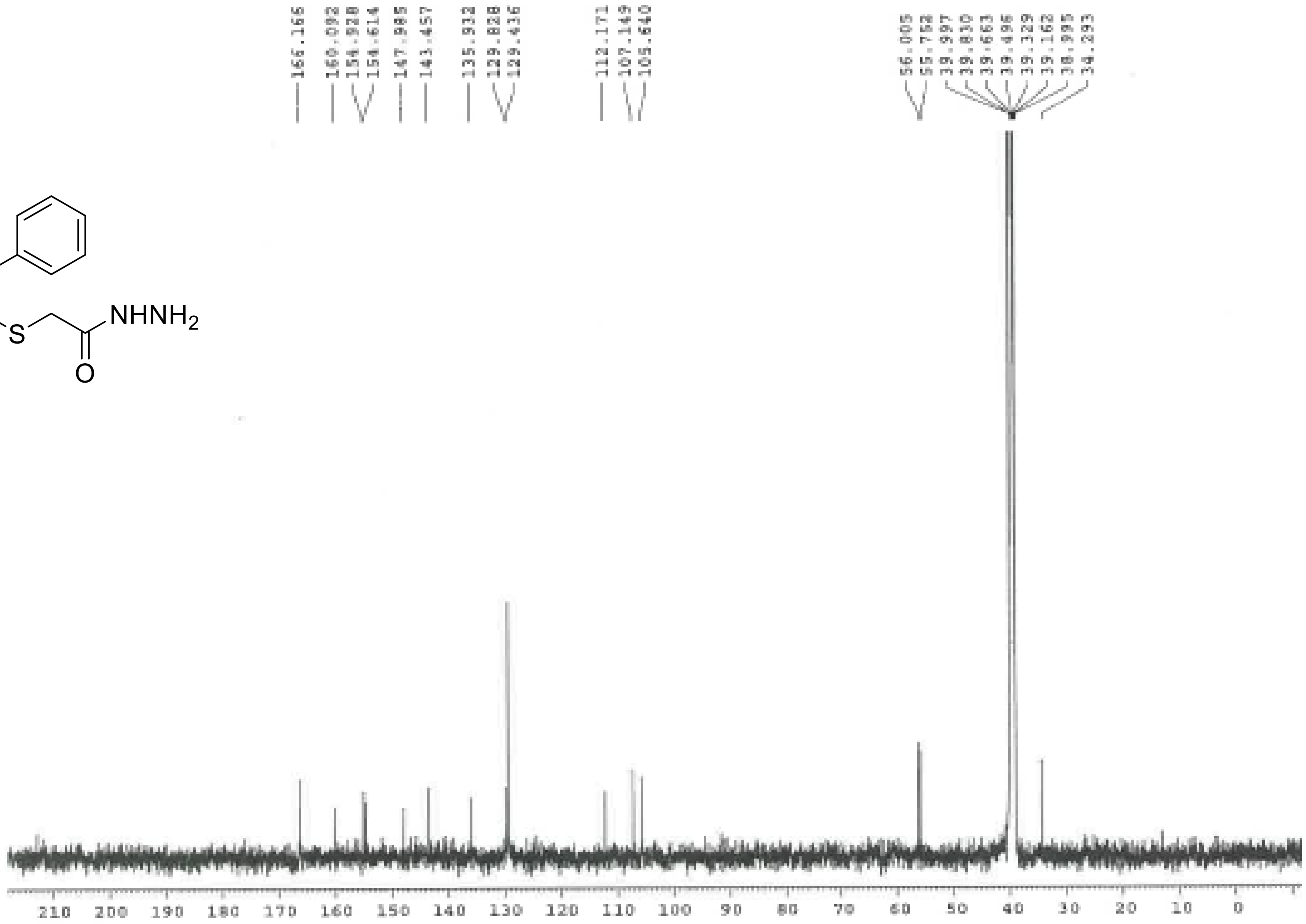

Figure S52. ^{13}CNMR of compound **3d** (extended)

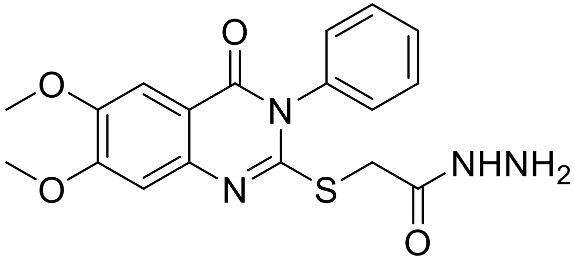

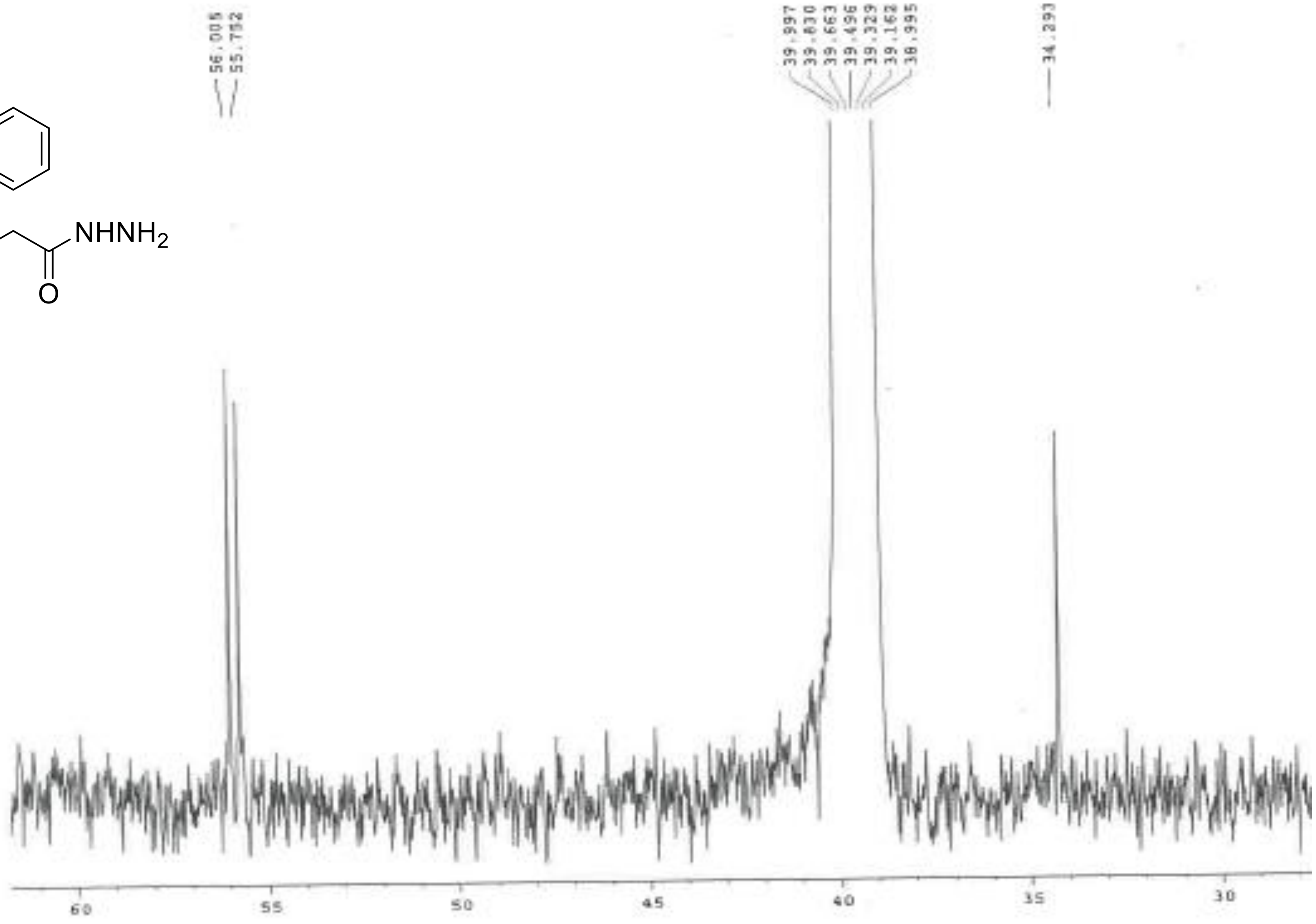

Figure S53. ^{13}CNMR of compound **3d** (extended)

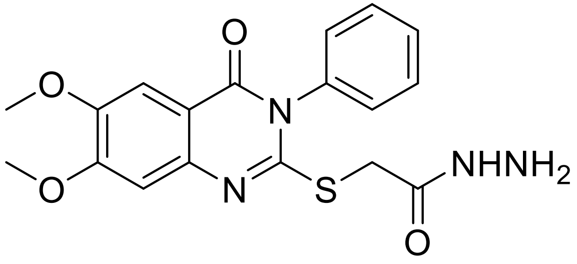

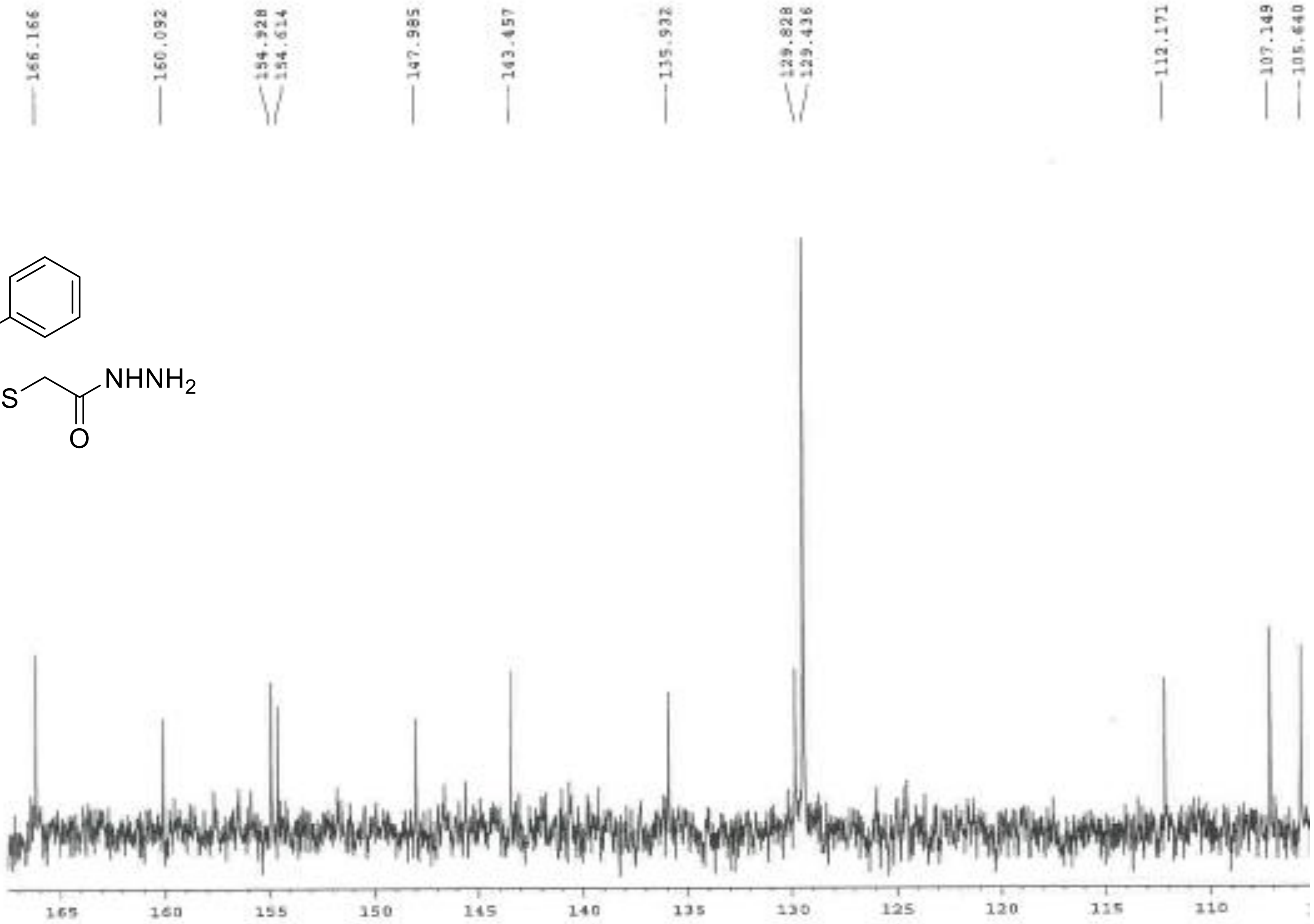

Figure S54. ^{1}HNMR of compound **3e**

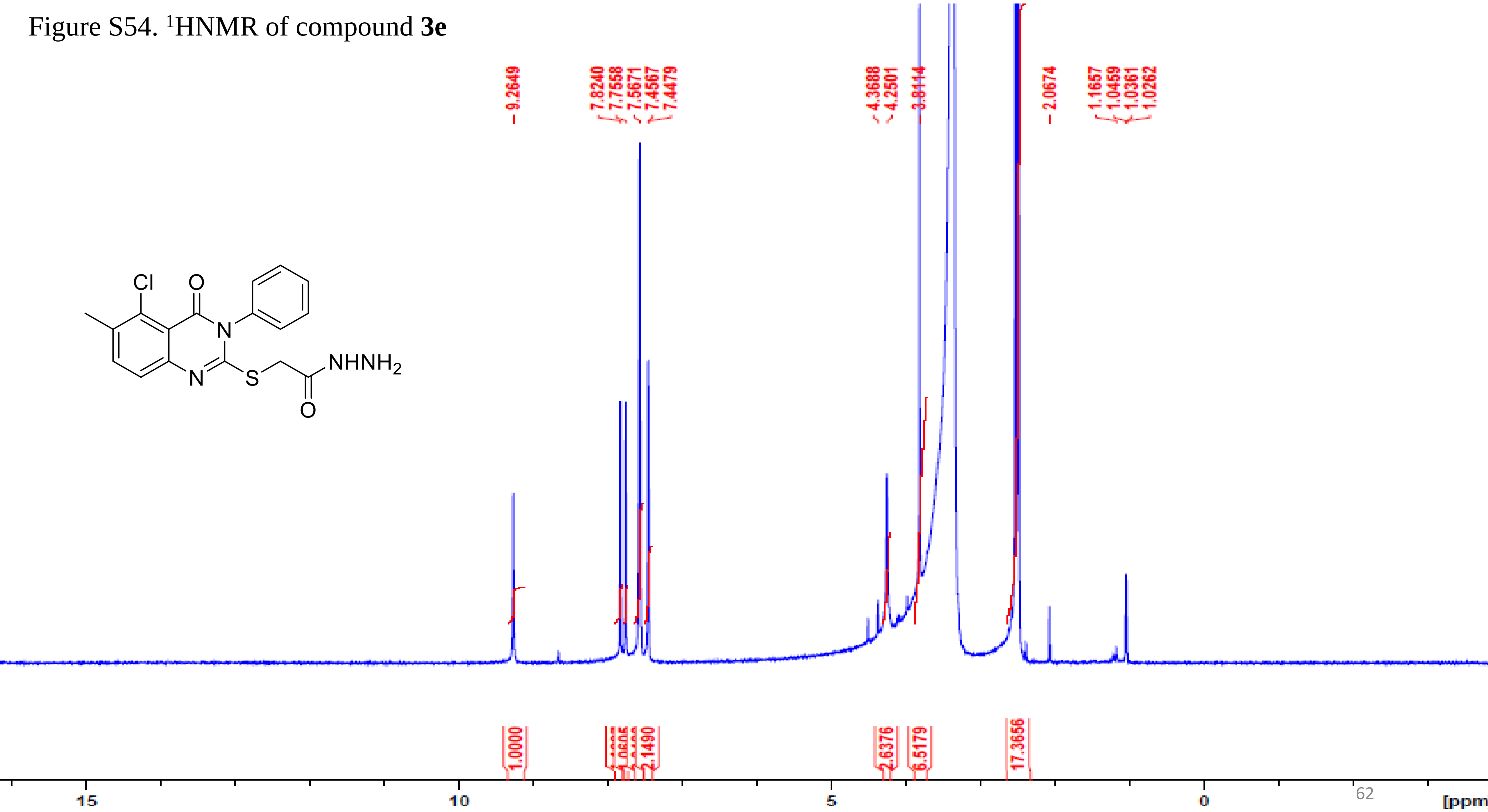

Figure S55. ^{1}HNMR of compound **3e** (extended)

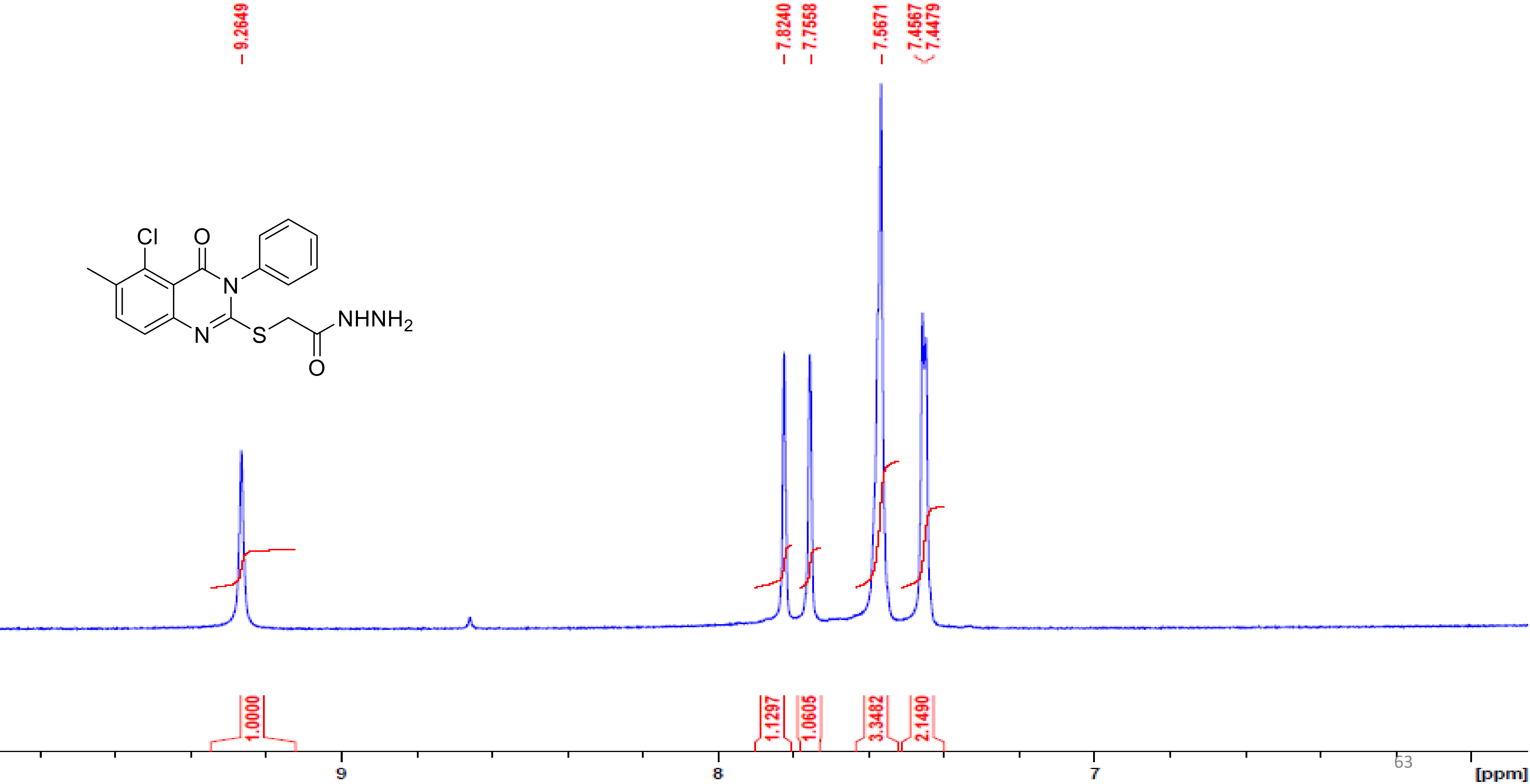

Figure S56. ^{13}CNMR of compound **3e**

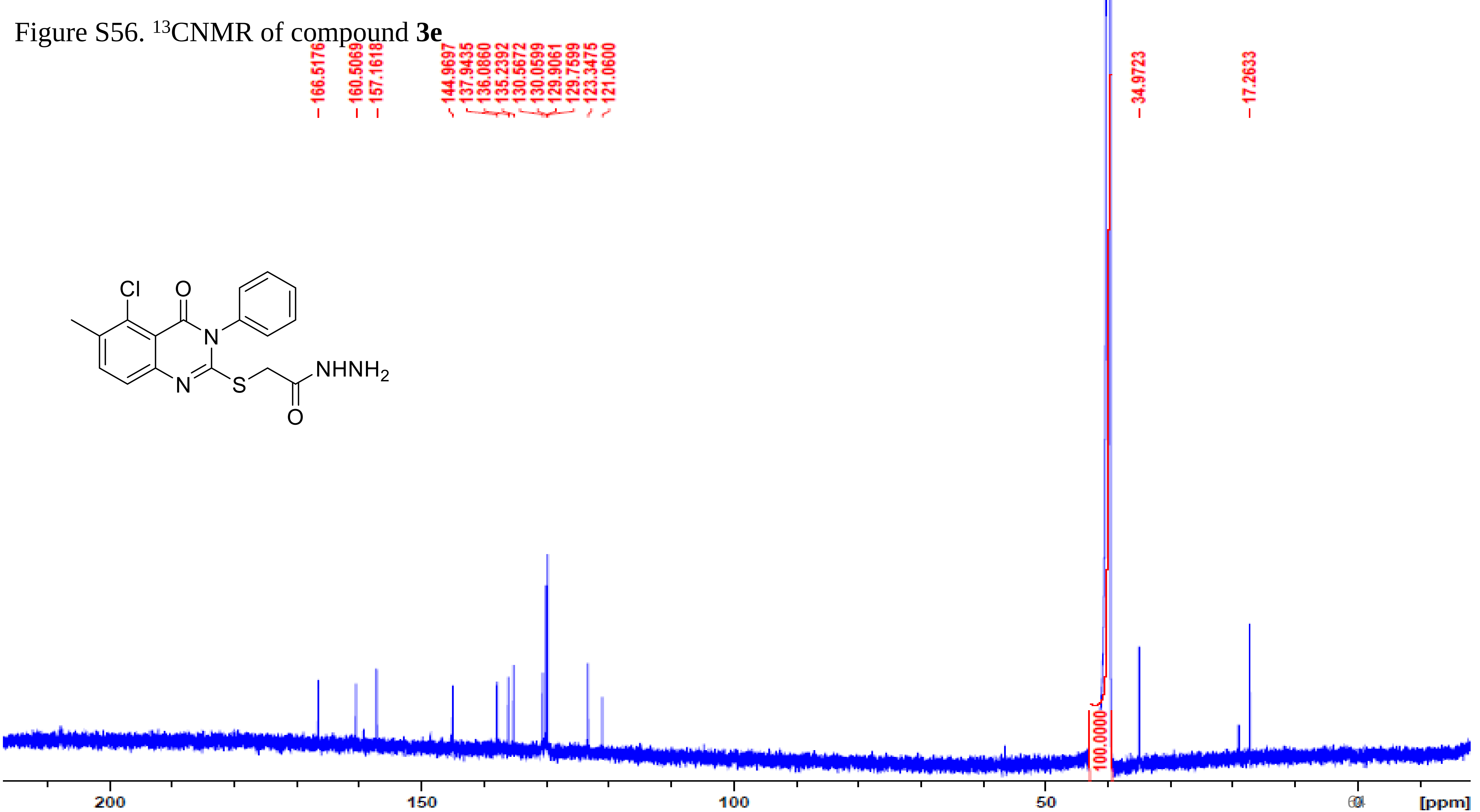

Figure S57. ^{13}CNMR of compound **3e** (extended)

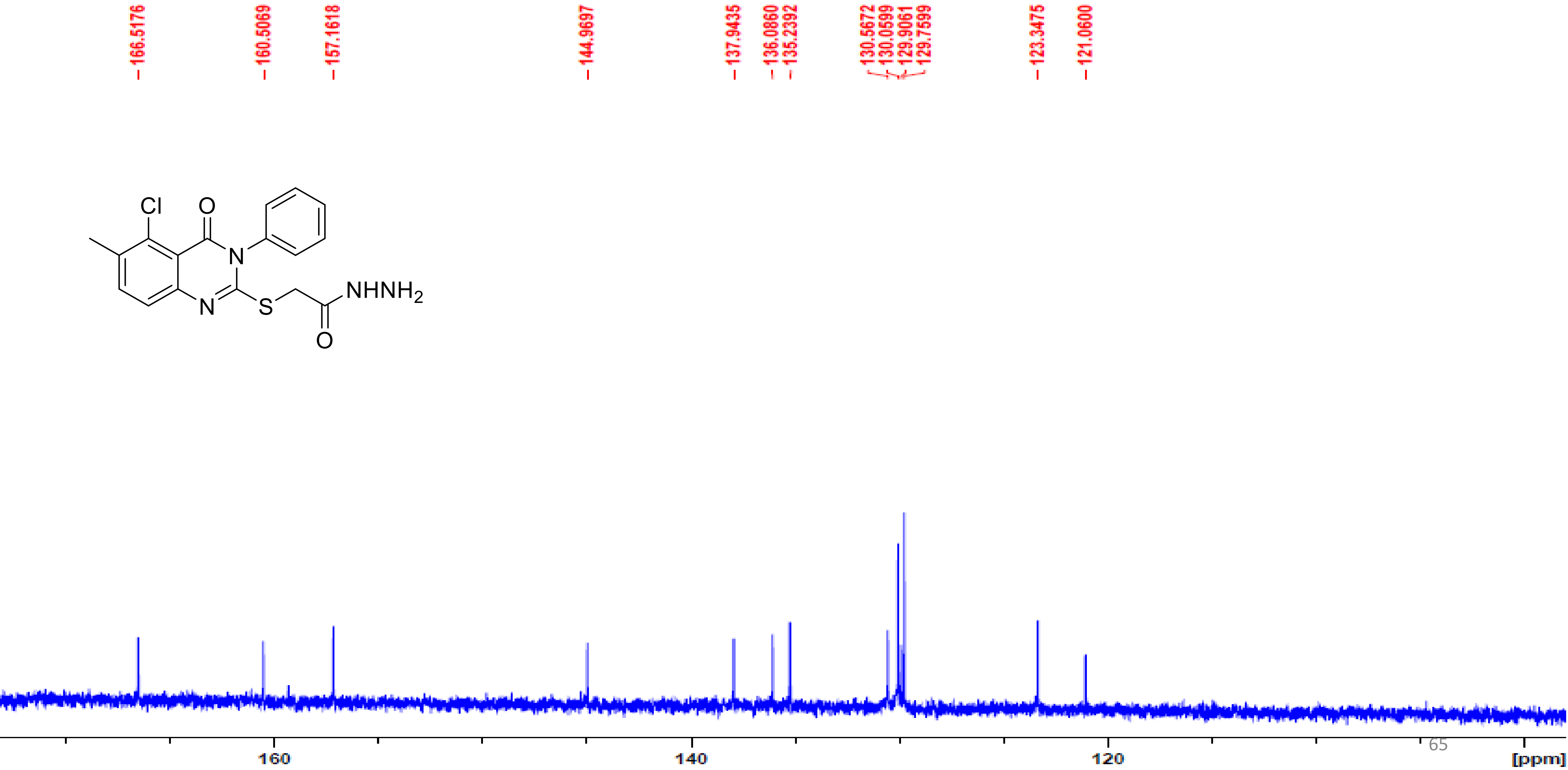

Figure S58. ^{1}HNMR of compound **3f**

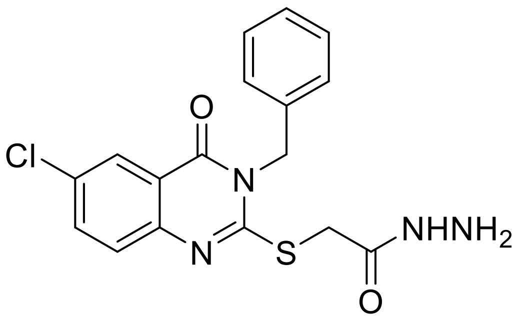

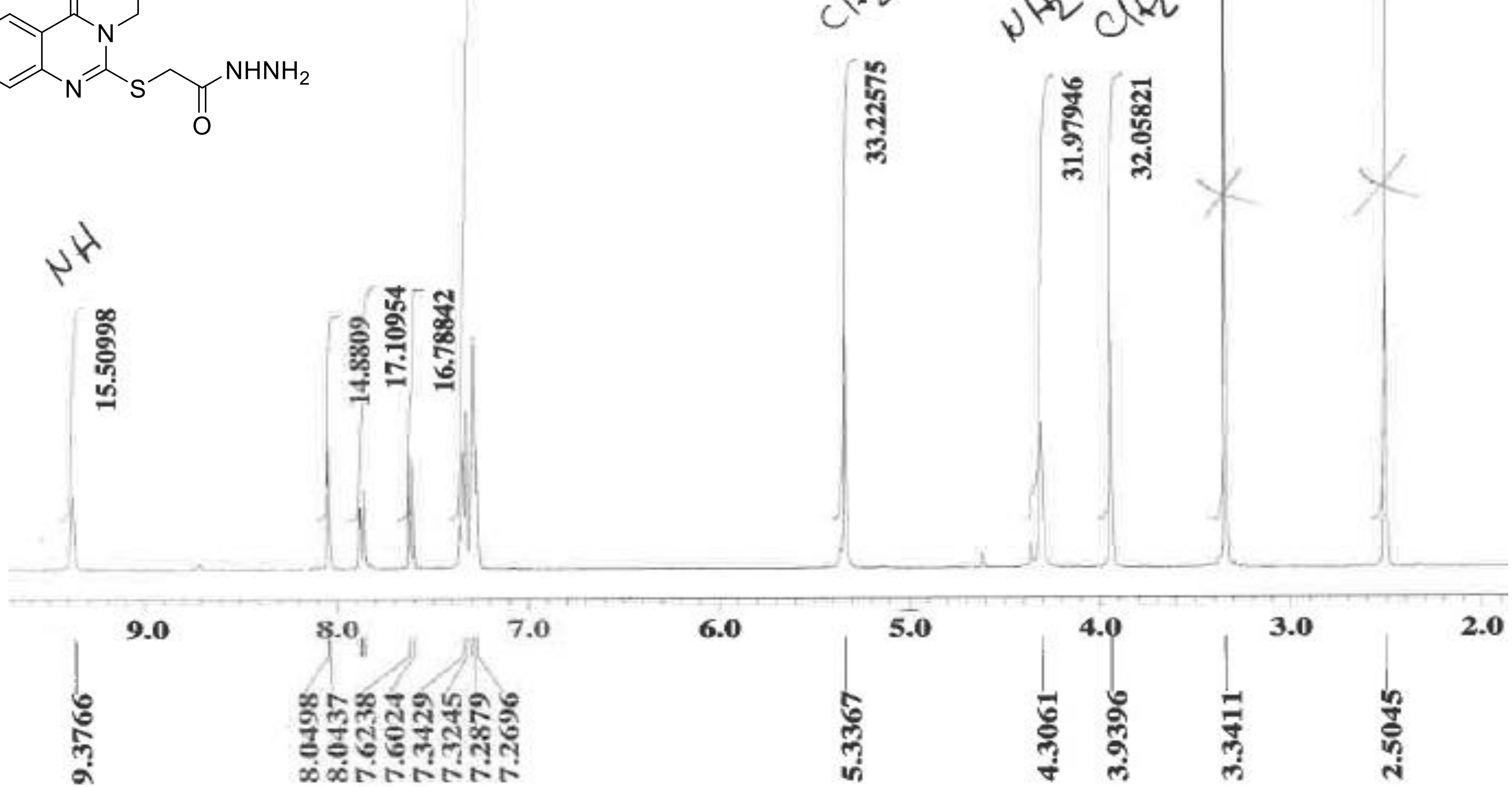

Figure S59. ^{1}HNMR of compound **3f (**extended**)**

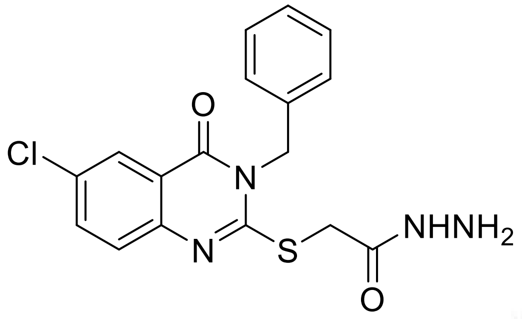

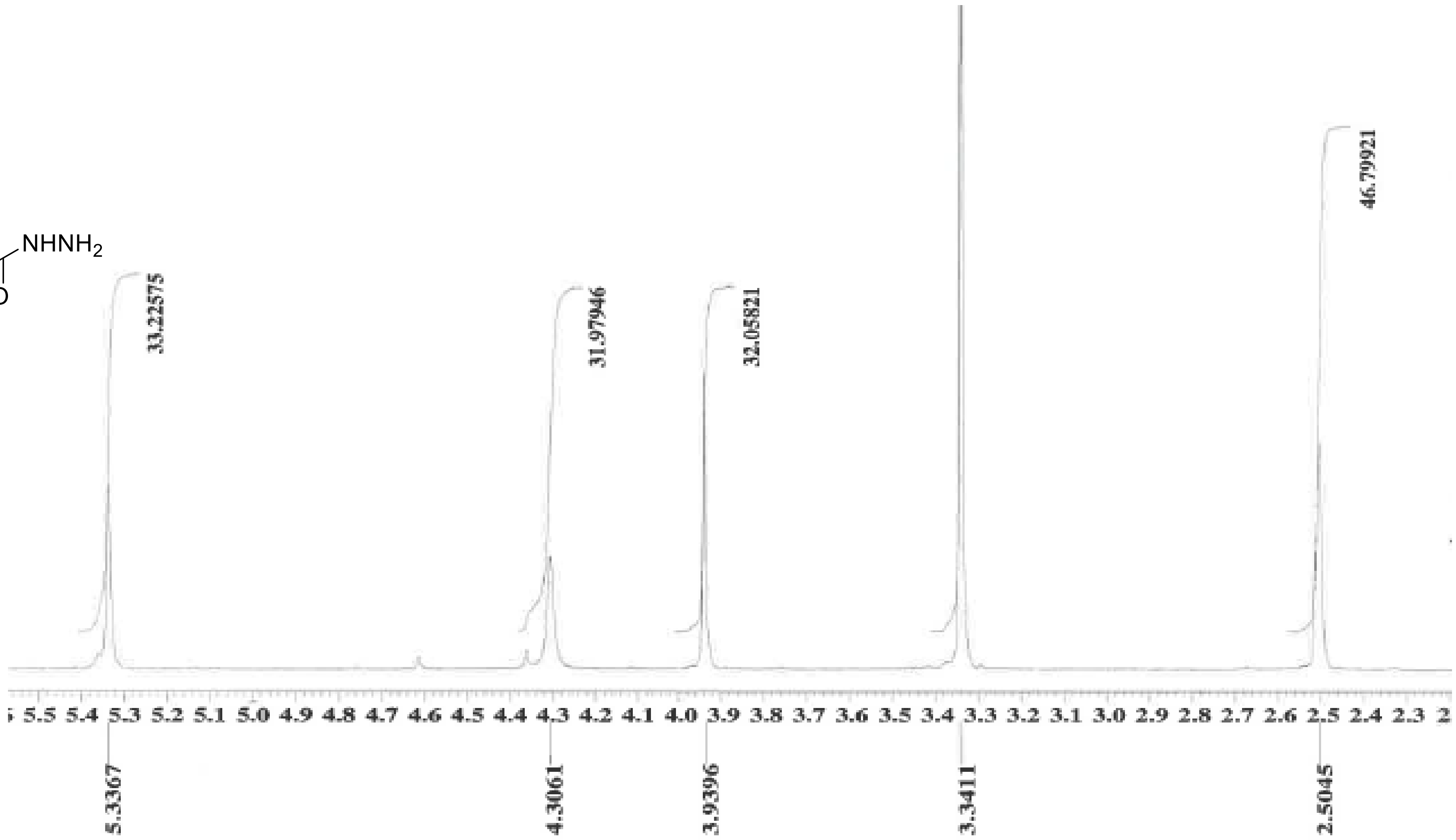

Figure S60. ^{1}HNMR of compound **3f (**extended**)**

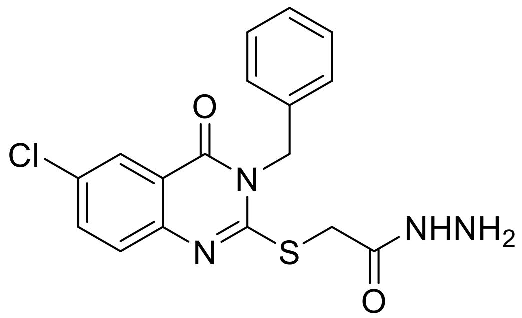

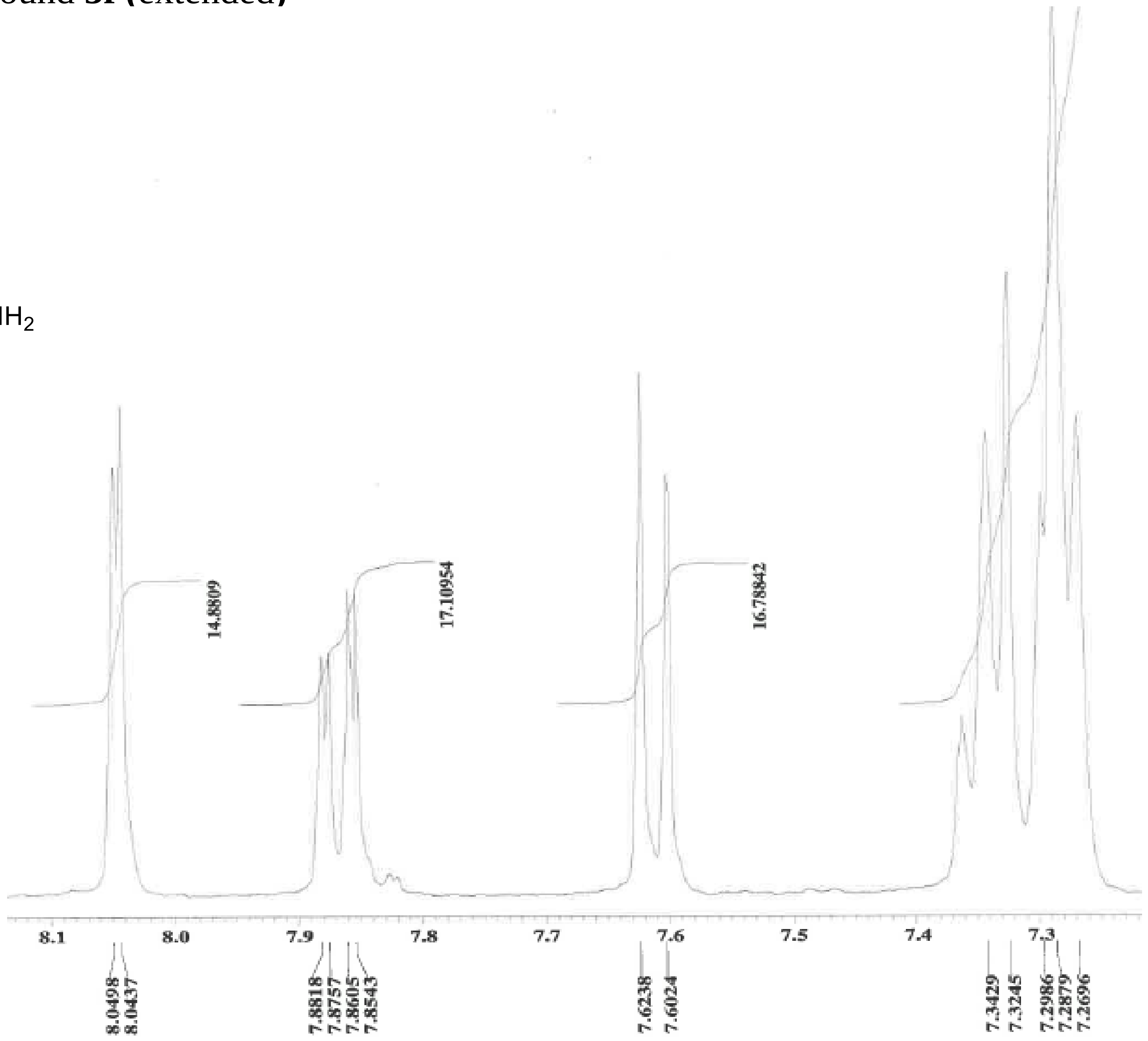

Figure S61. ^{13}CNMR of compound **3f**

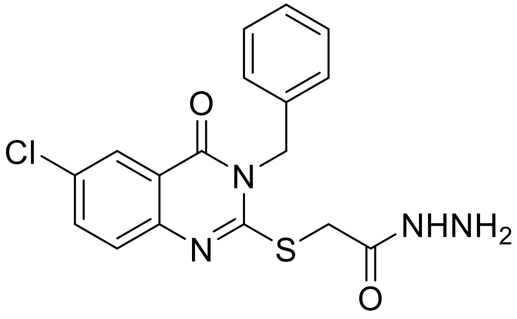

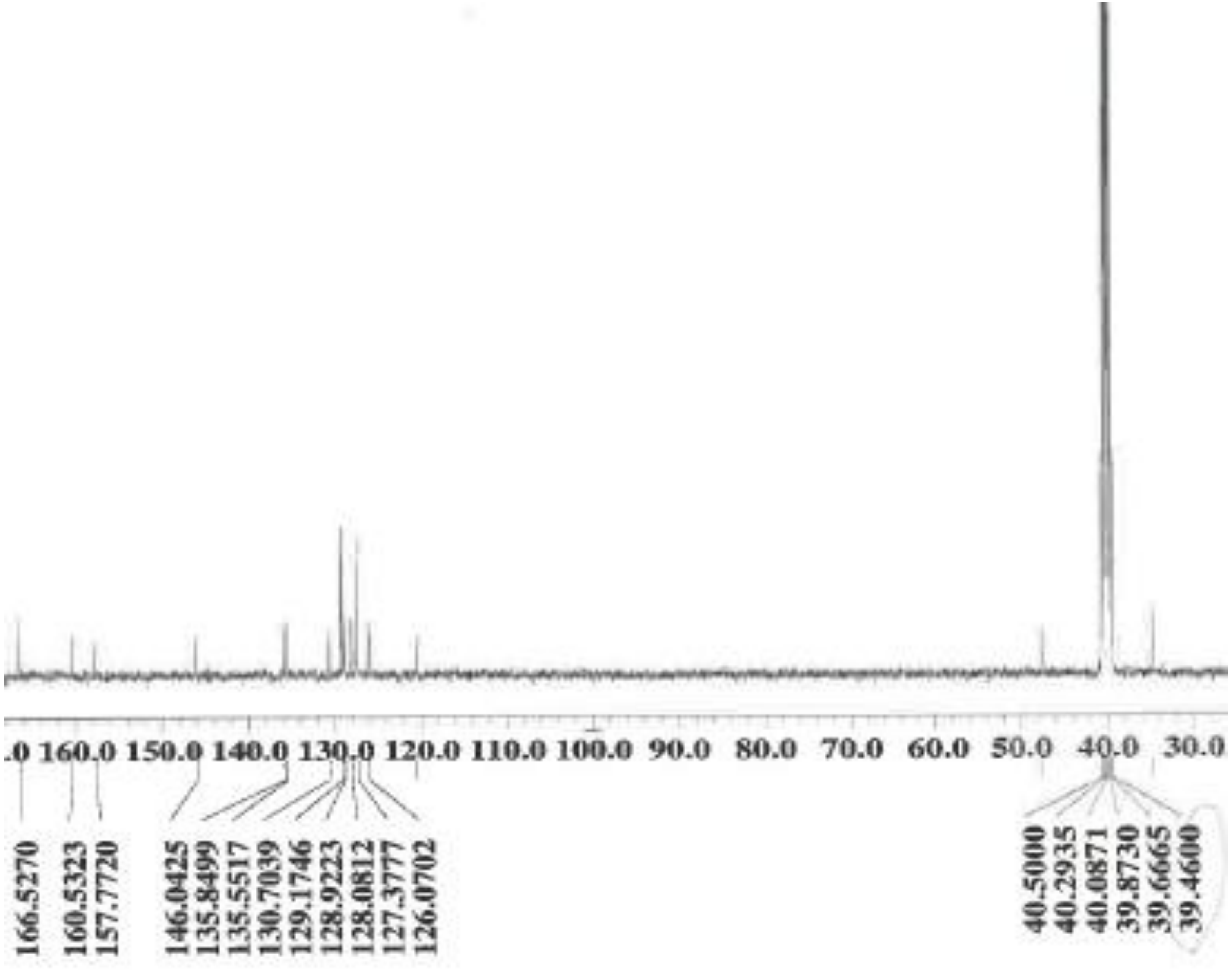

Figure S62. ^{13}CNMR of compound **3f** (extended)

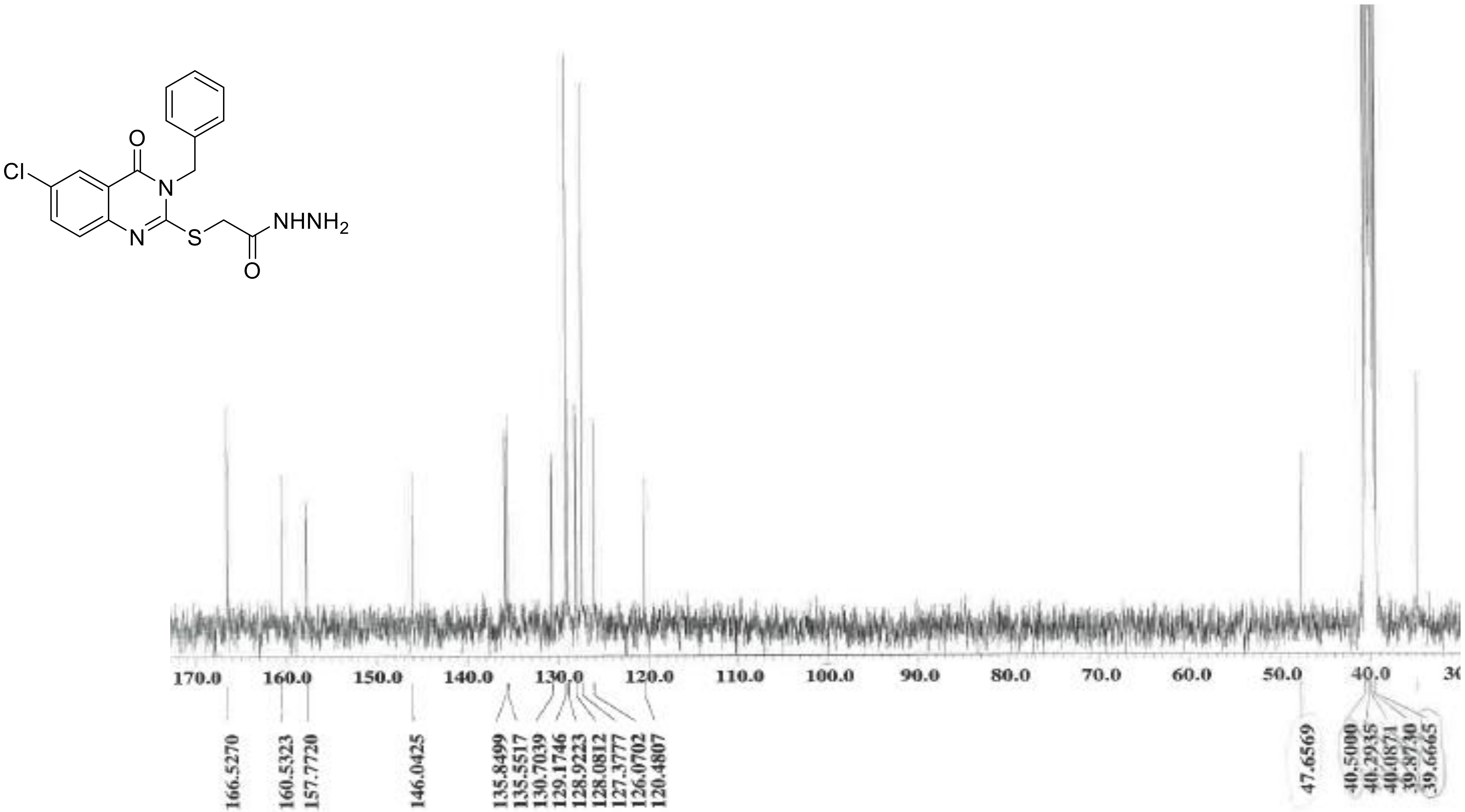

Figure S63. ^{13}CNMR of compound **3f** (extended)

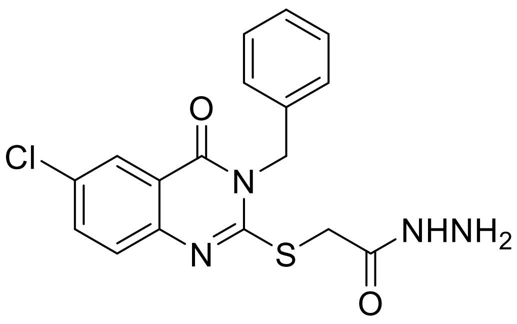

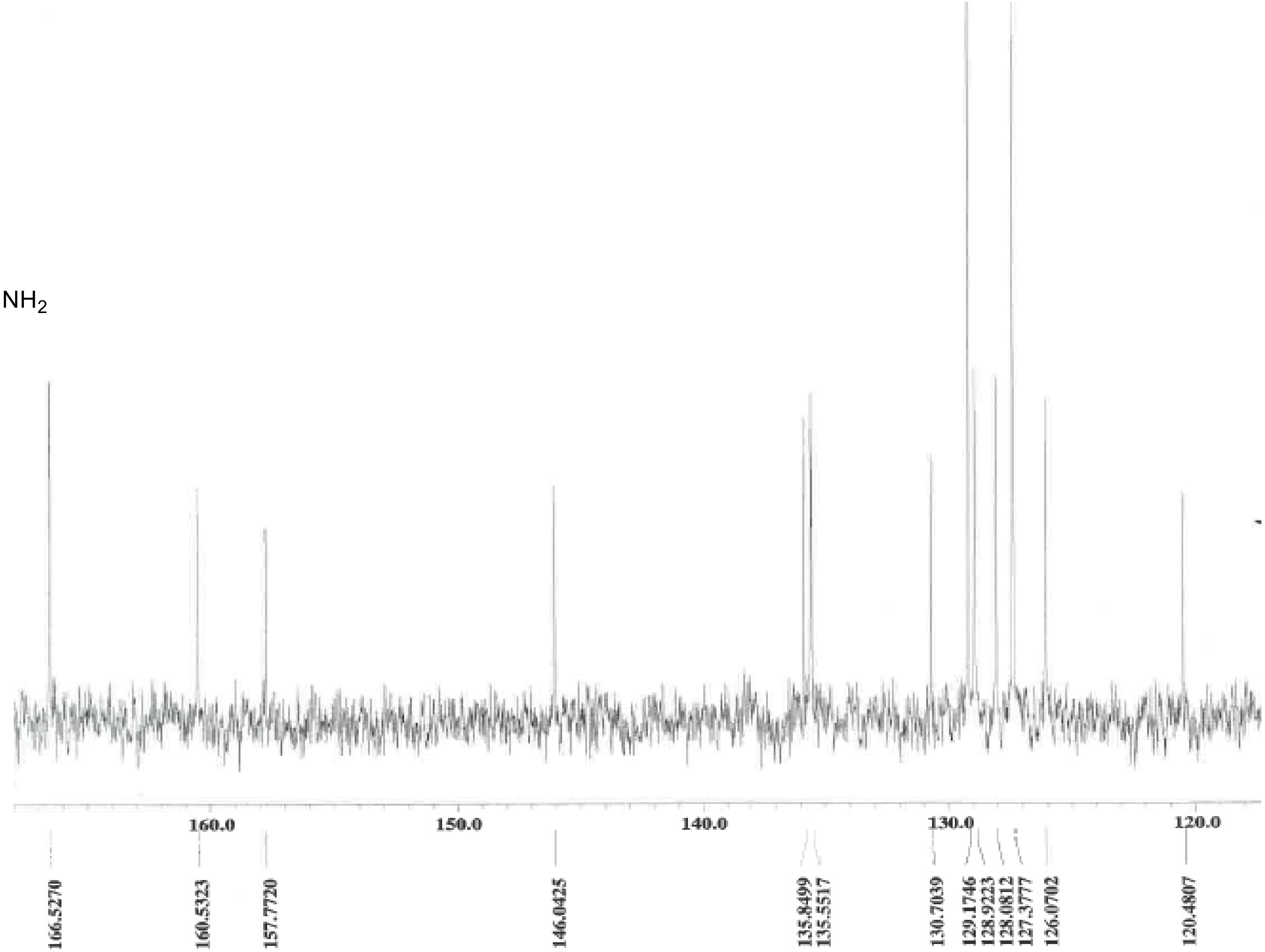

Figure S64. ^{1}HNMR of compound **3g**

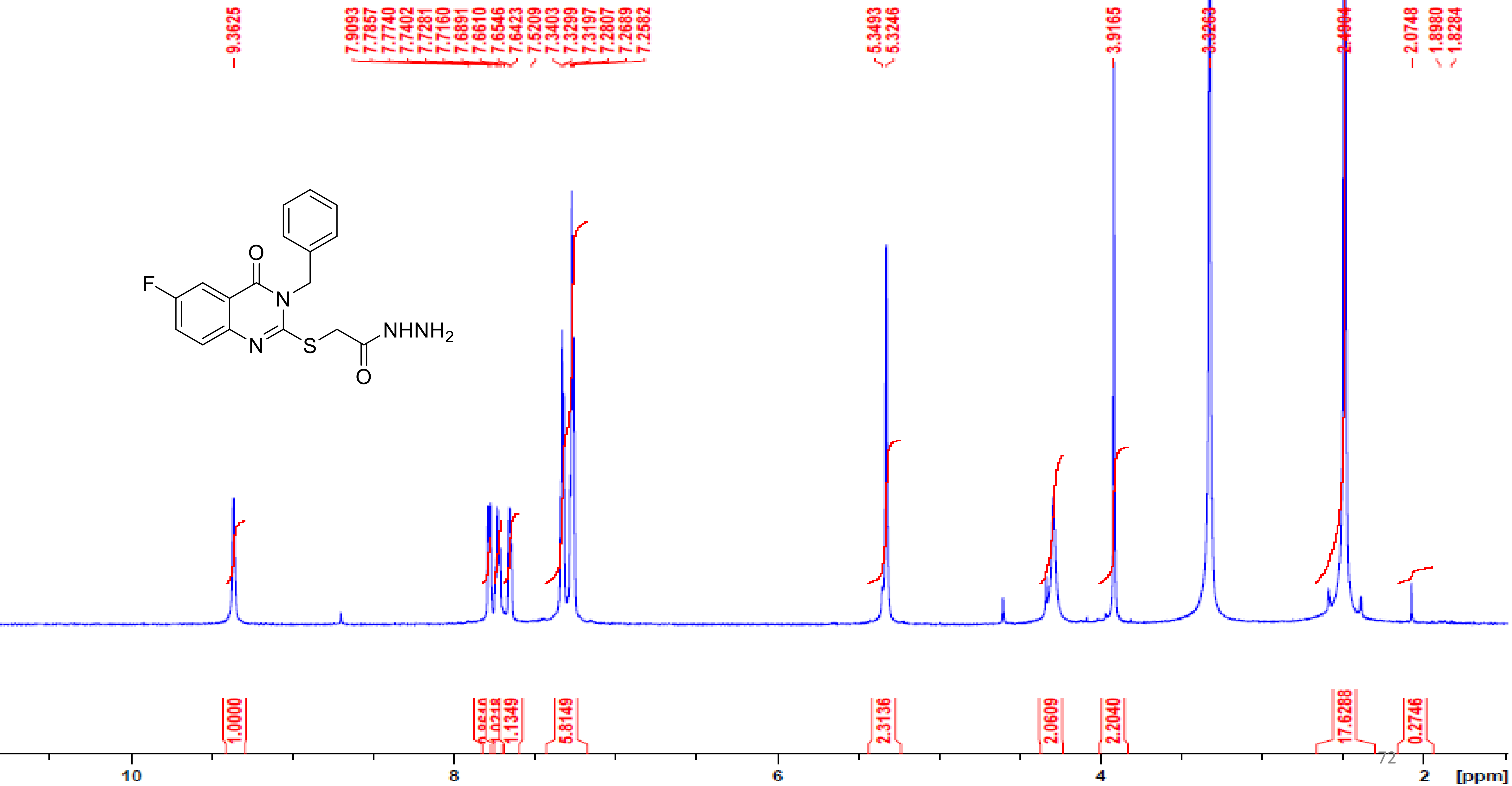

Figure S65. ^{1}HNMR of compound **3g (**extended**)**

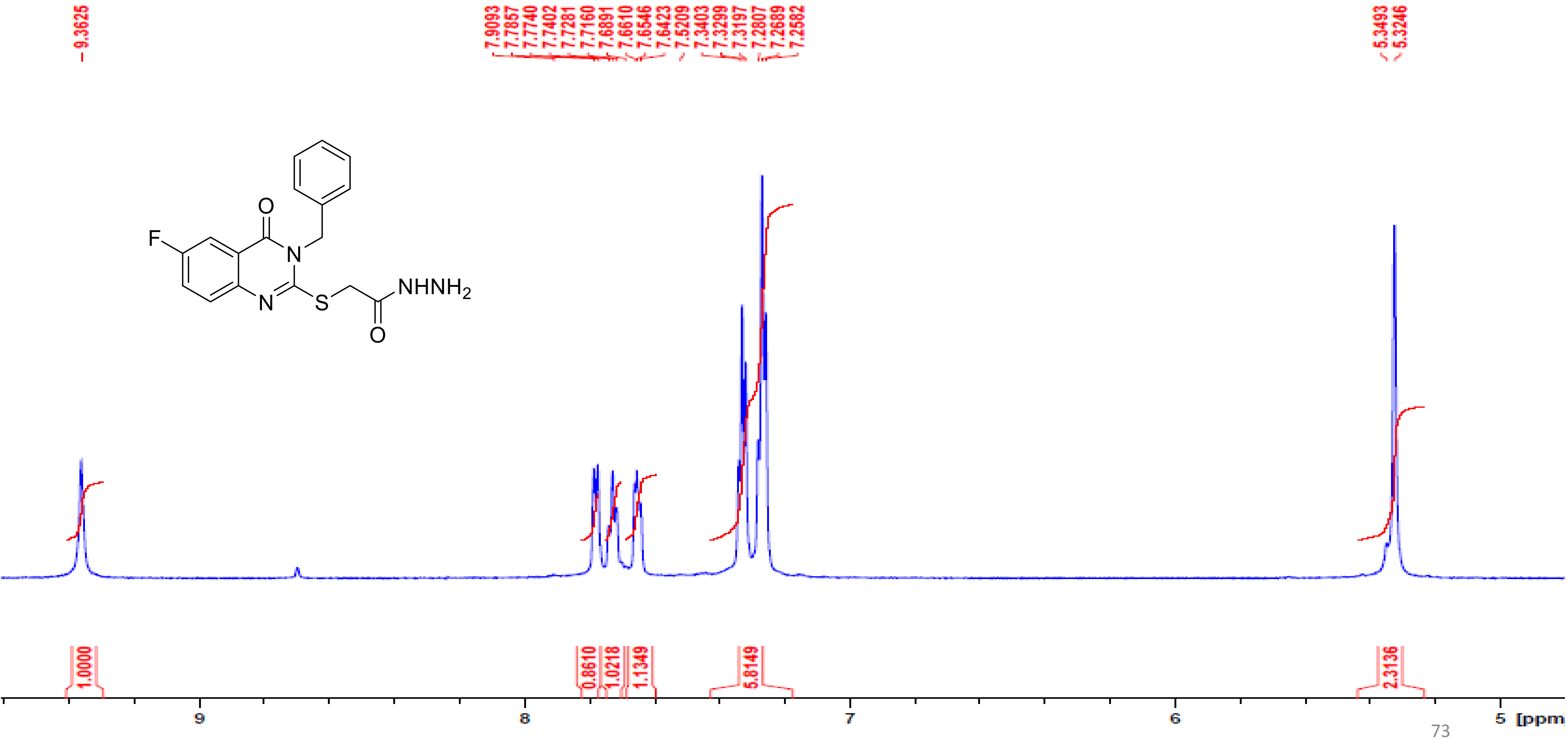

Figure S66. ^{1}HNMR of compound **3h**

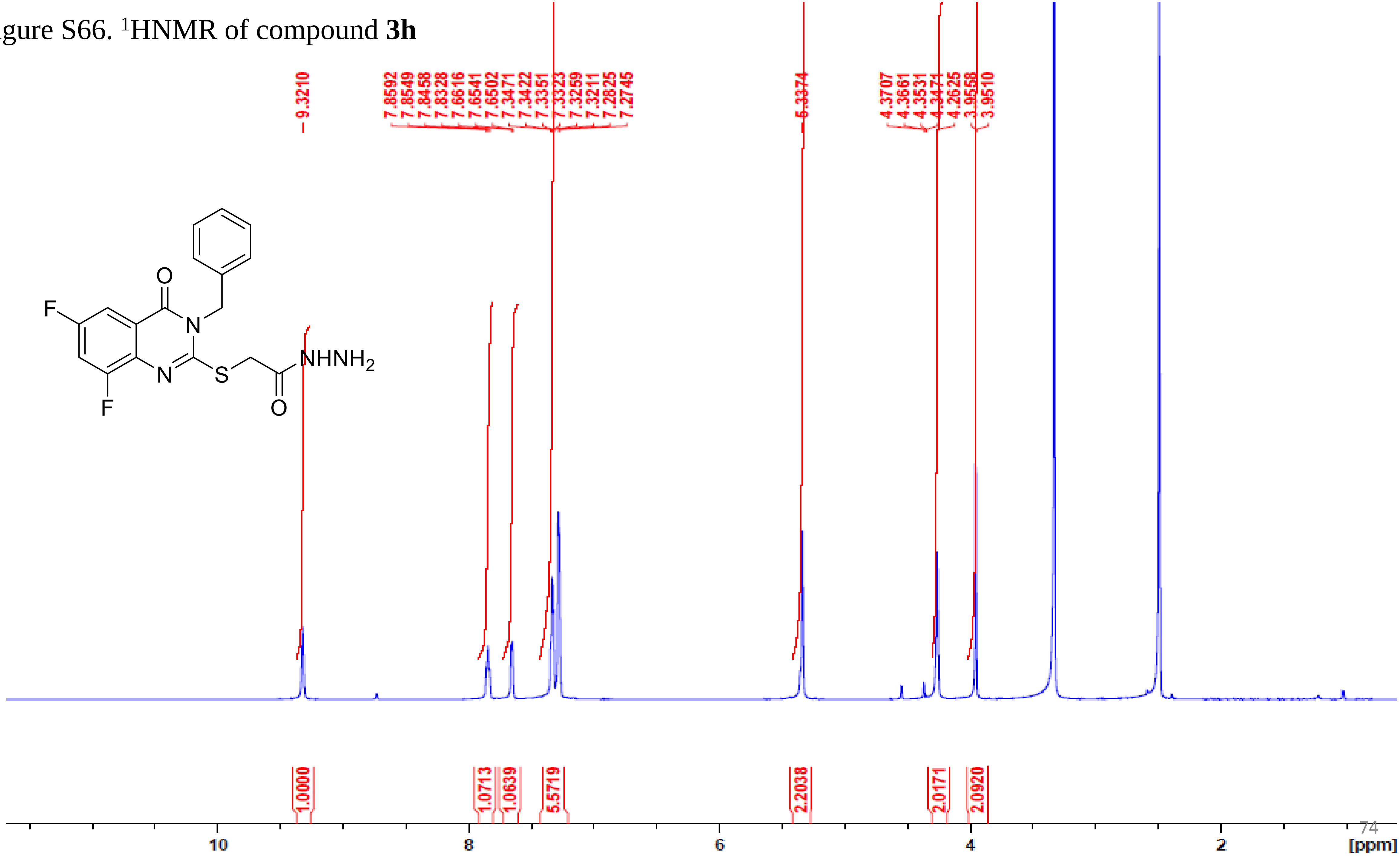

Figure S67. ^{1}HNMR of compound **3h (**extended**)**

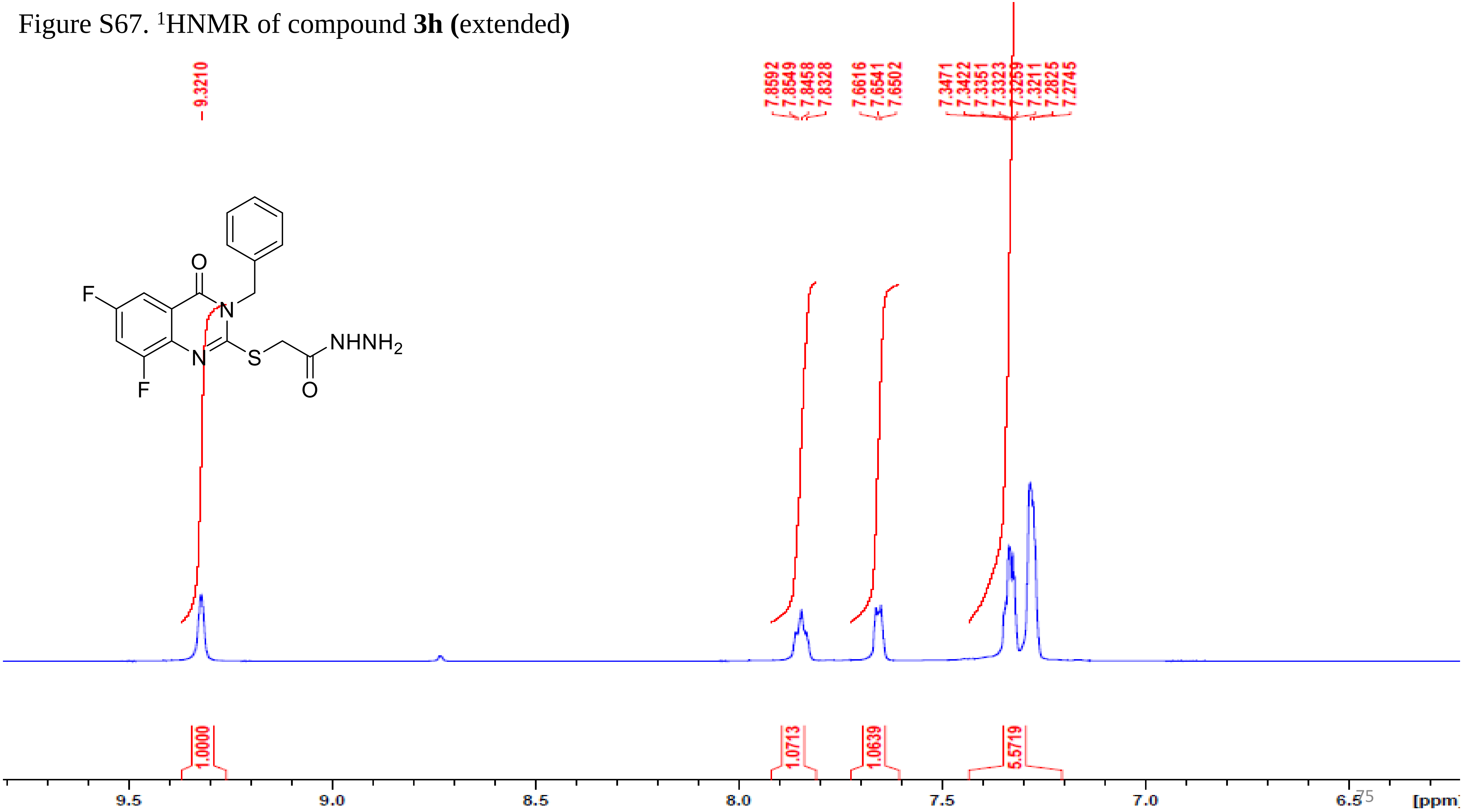

Figure S68. ^{13}CNMR of compound **3h**

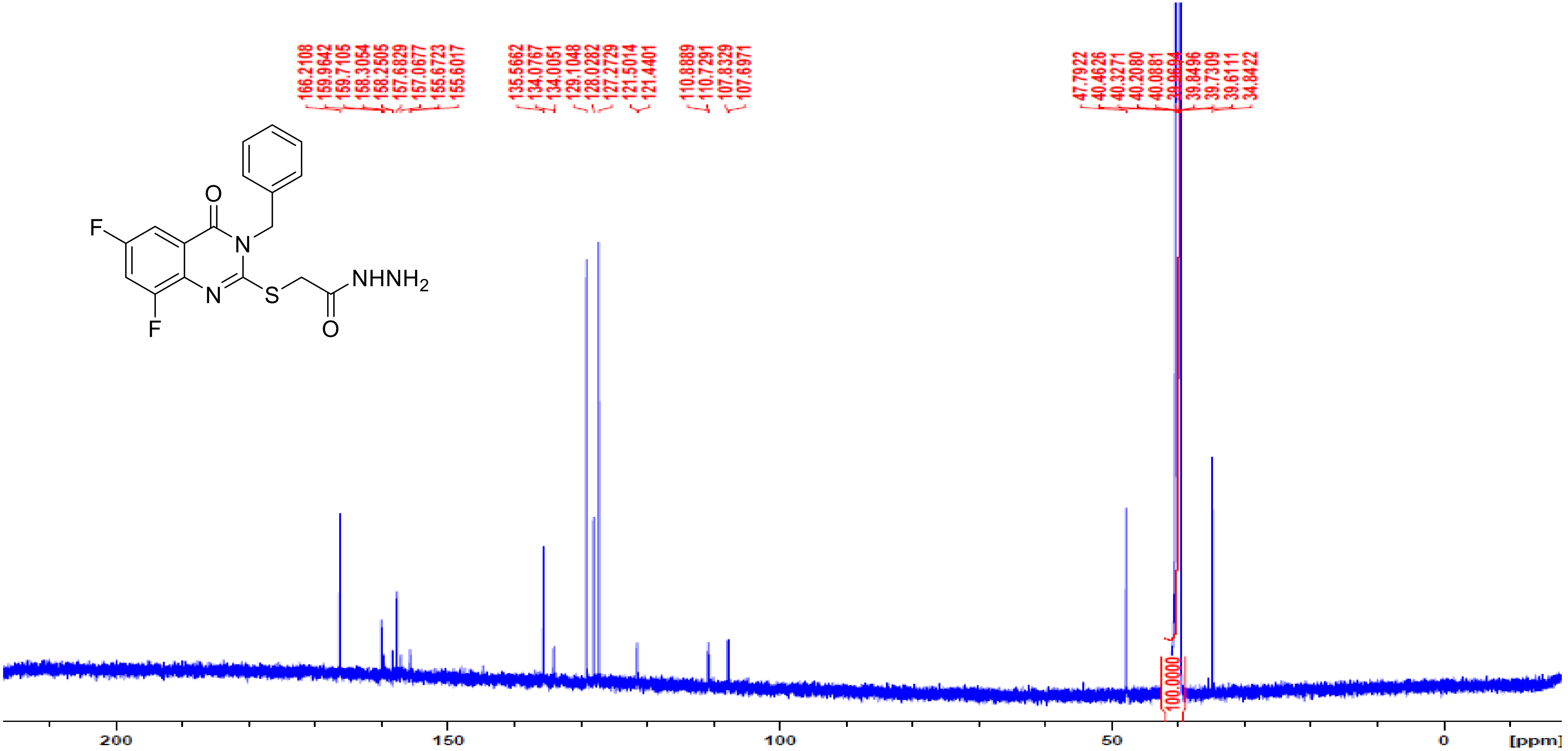

Figure S69. ^{13}CNMR of compound **3h** (extended)

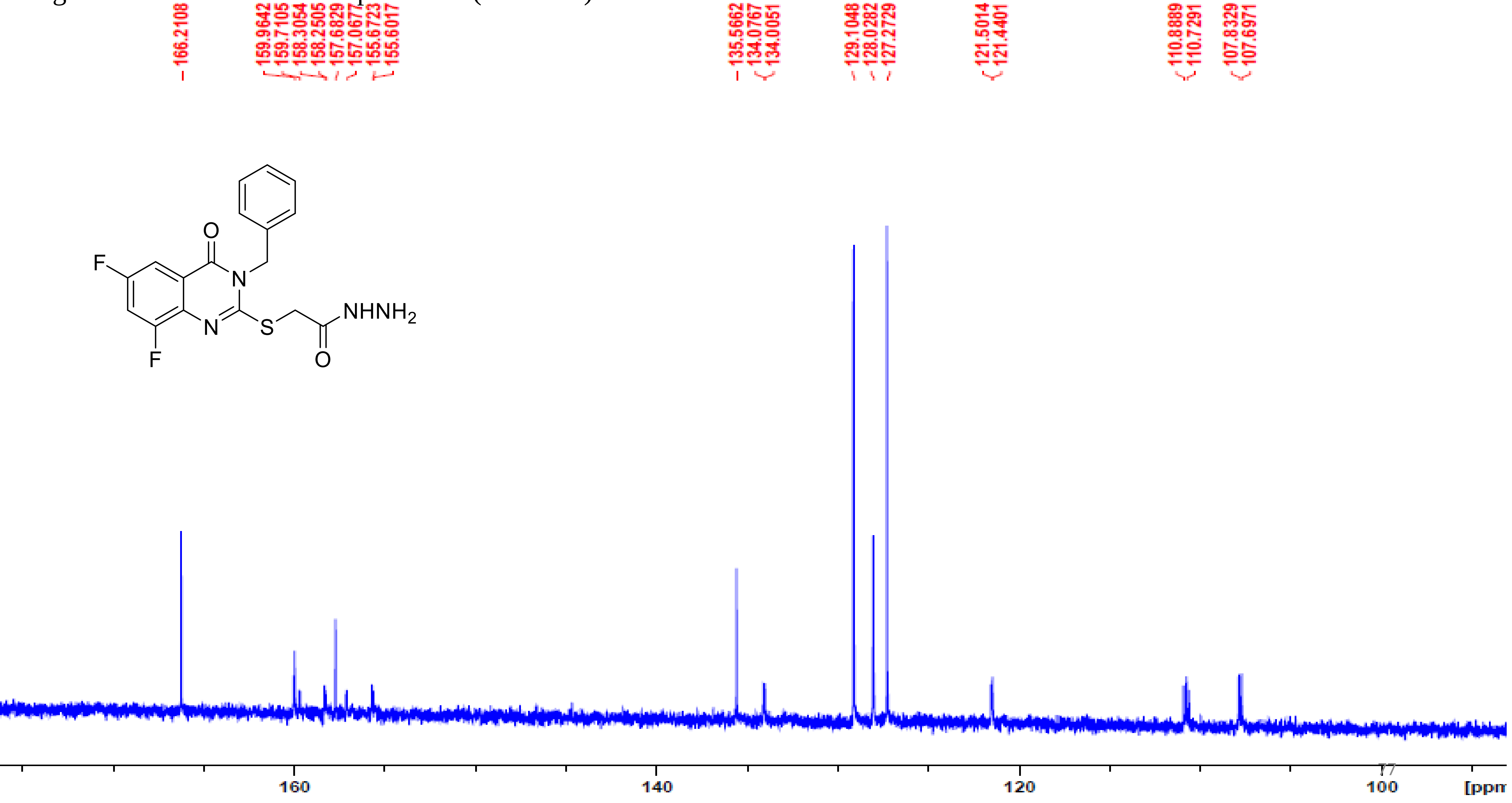

Figure S70. ^{1}HNMR of compound **3i**

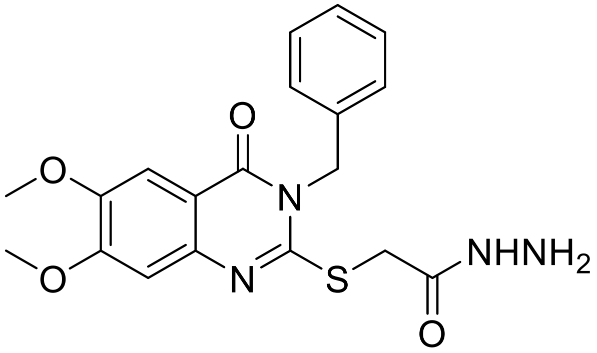

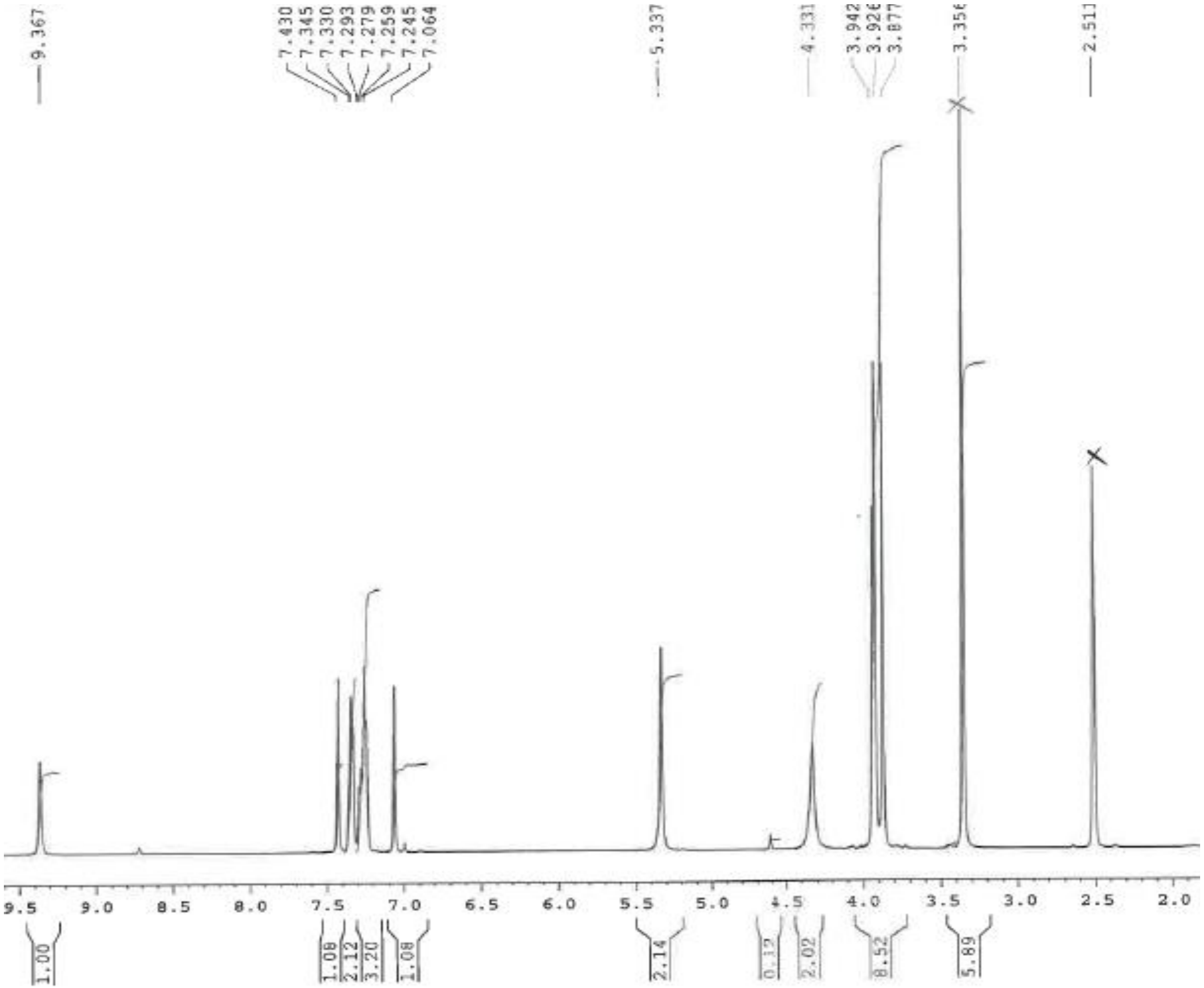

Figure S71. ^{1}HNMR of compound **3i (**extended**)**

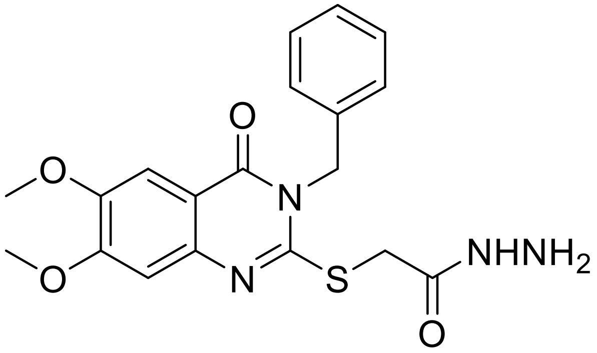

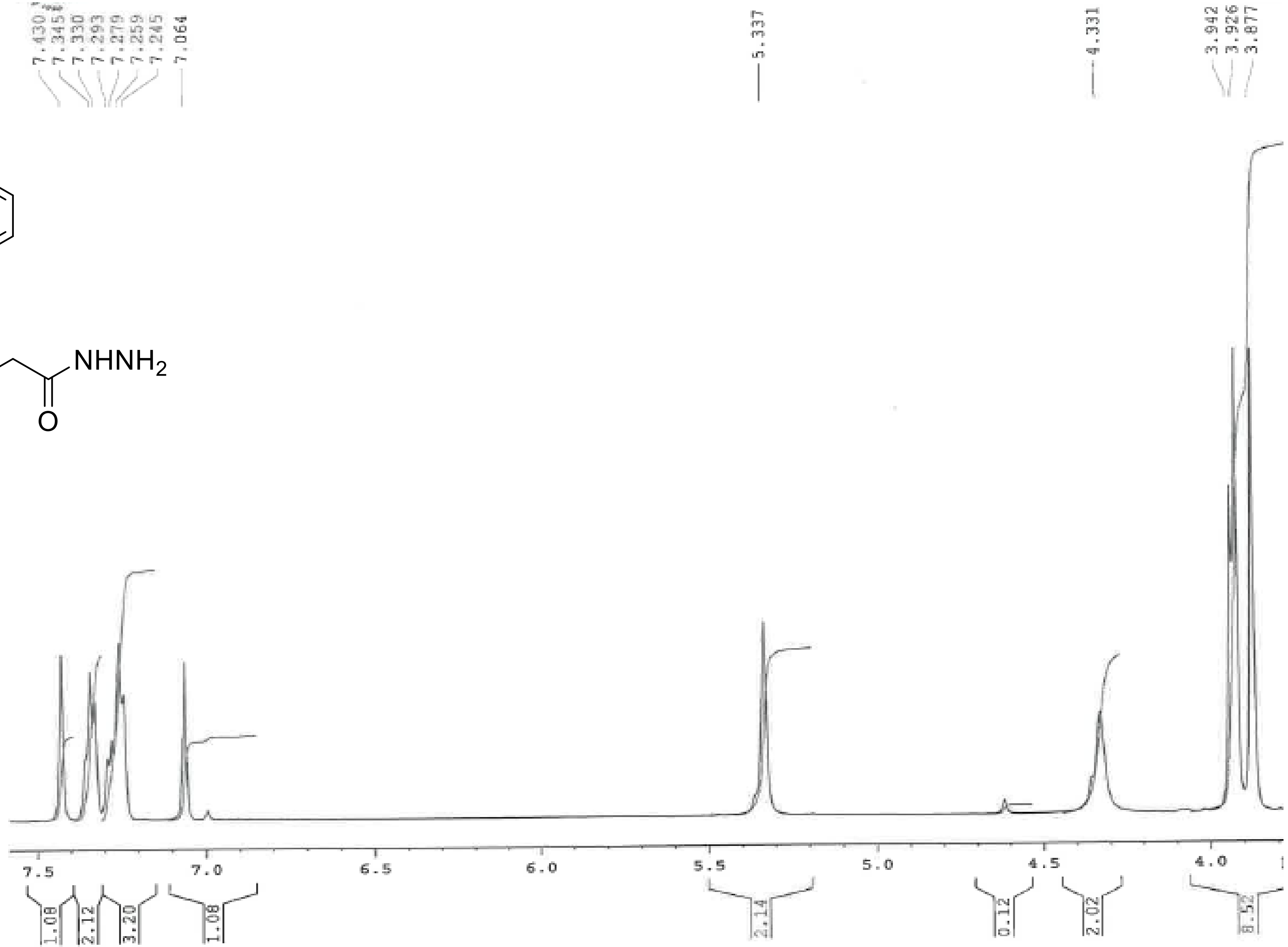

Figure S72. ^{13}CNMR of compound **3i**

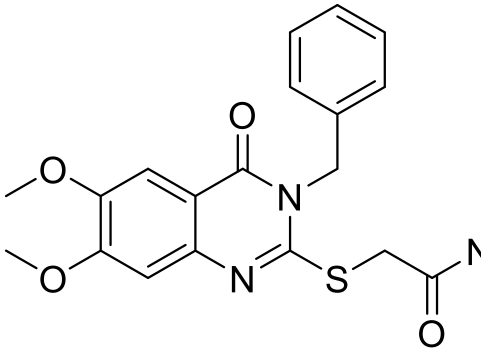

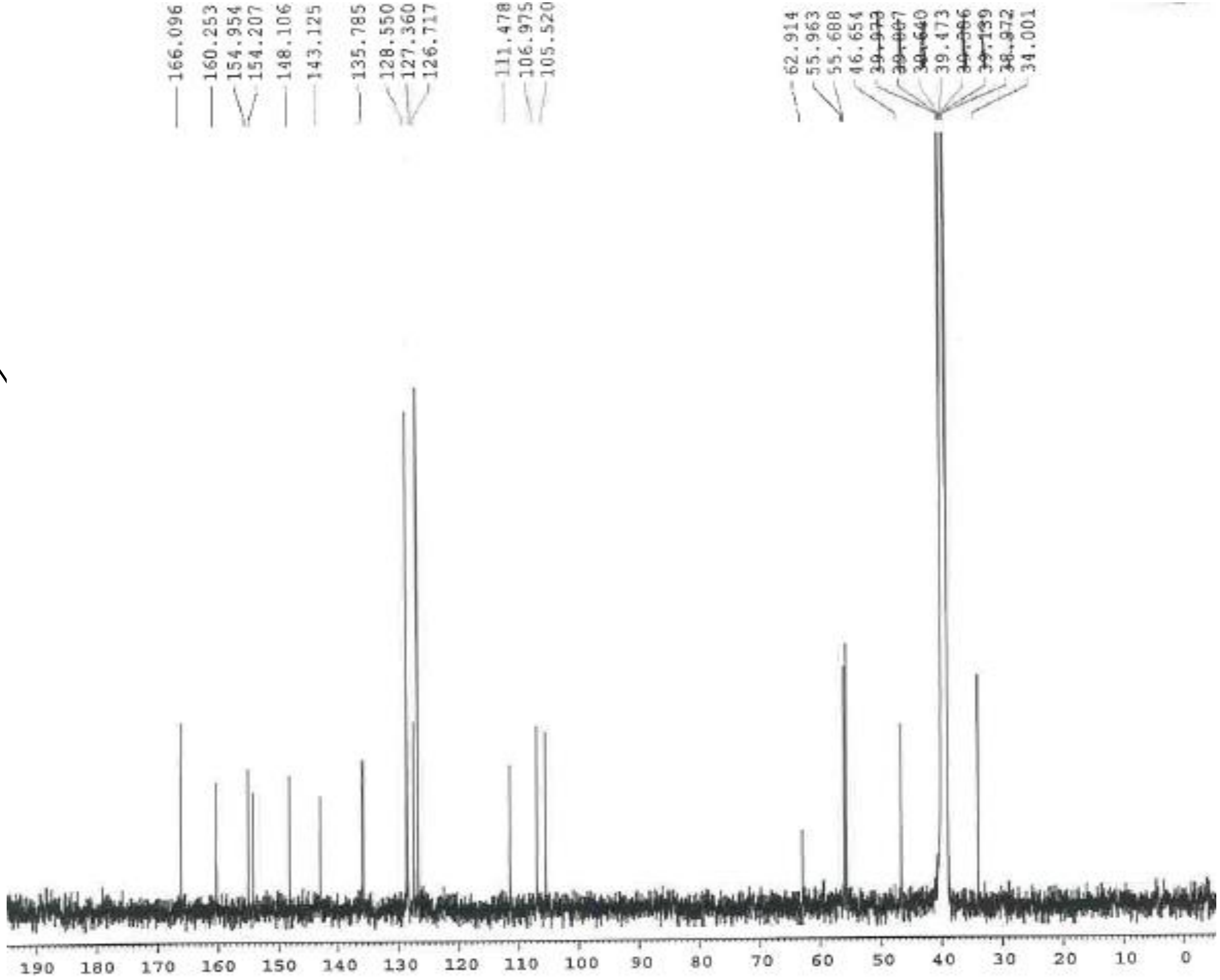

Figure S73. ^{13}CNMR of compound **3i** (extended)

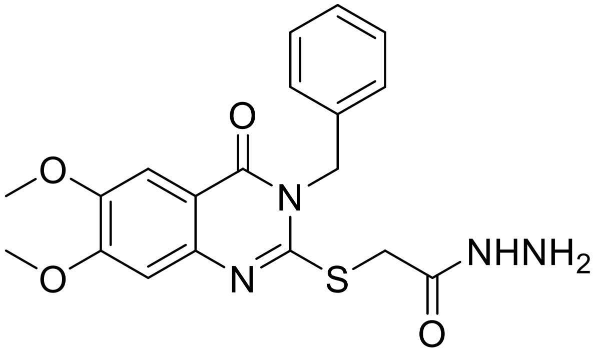

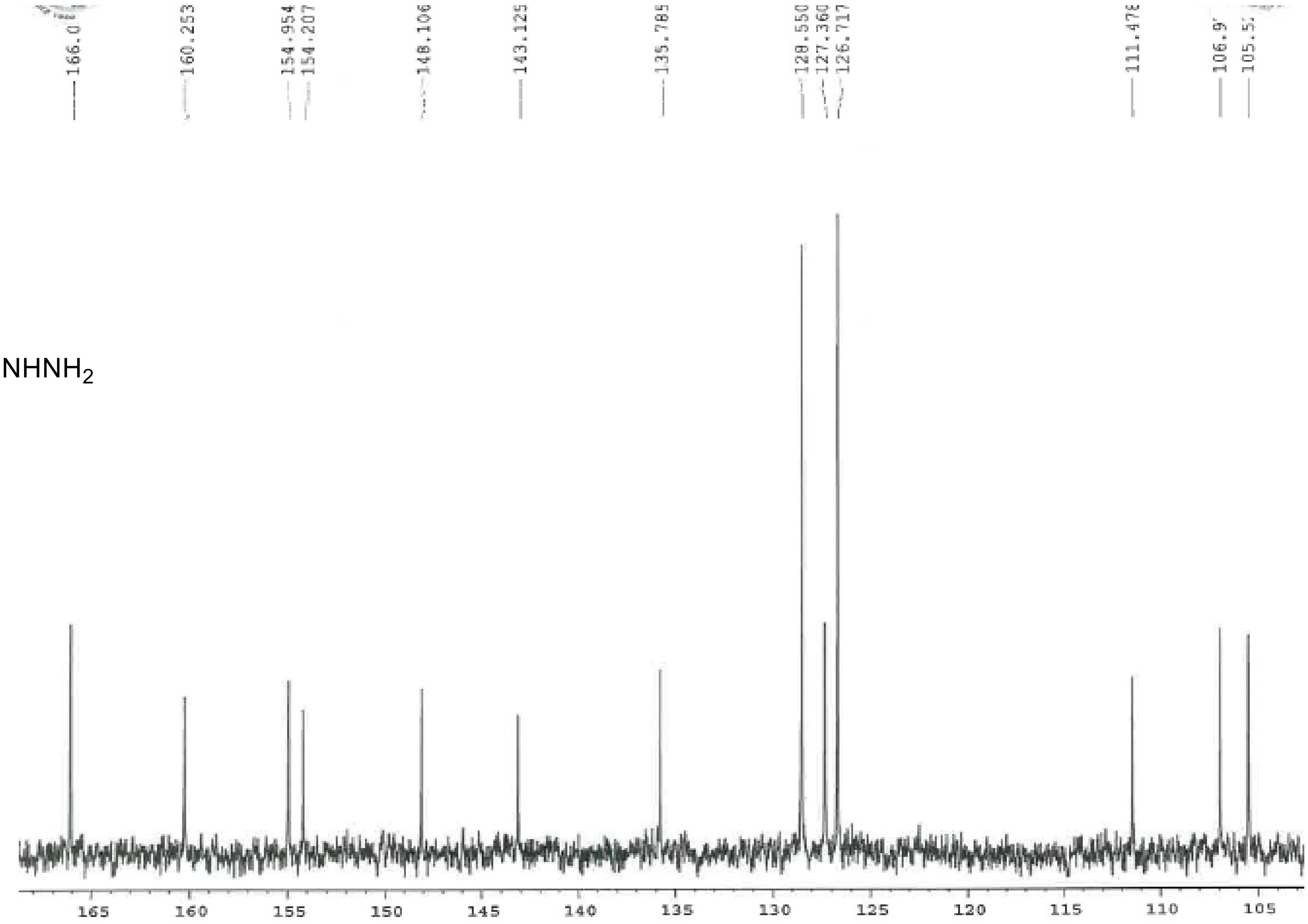

Figure S74. ^{13}CNMR of compound **3i** (extended)

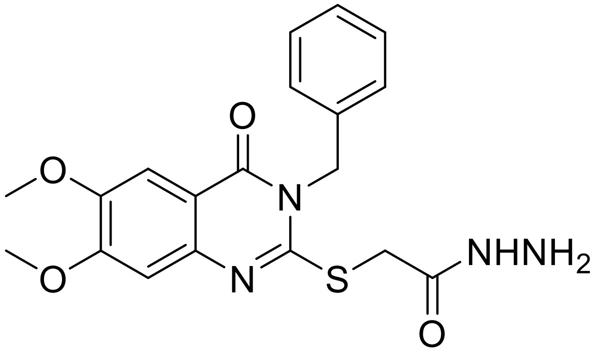

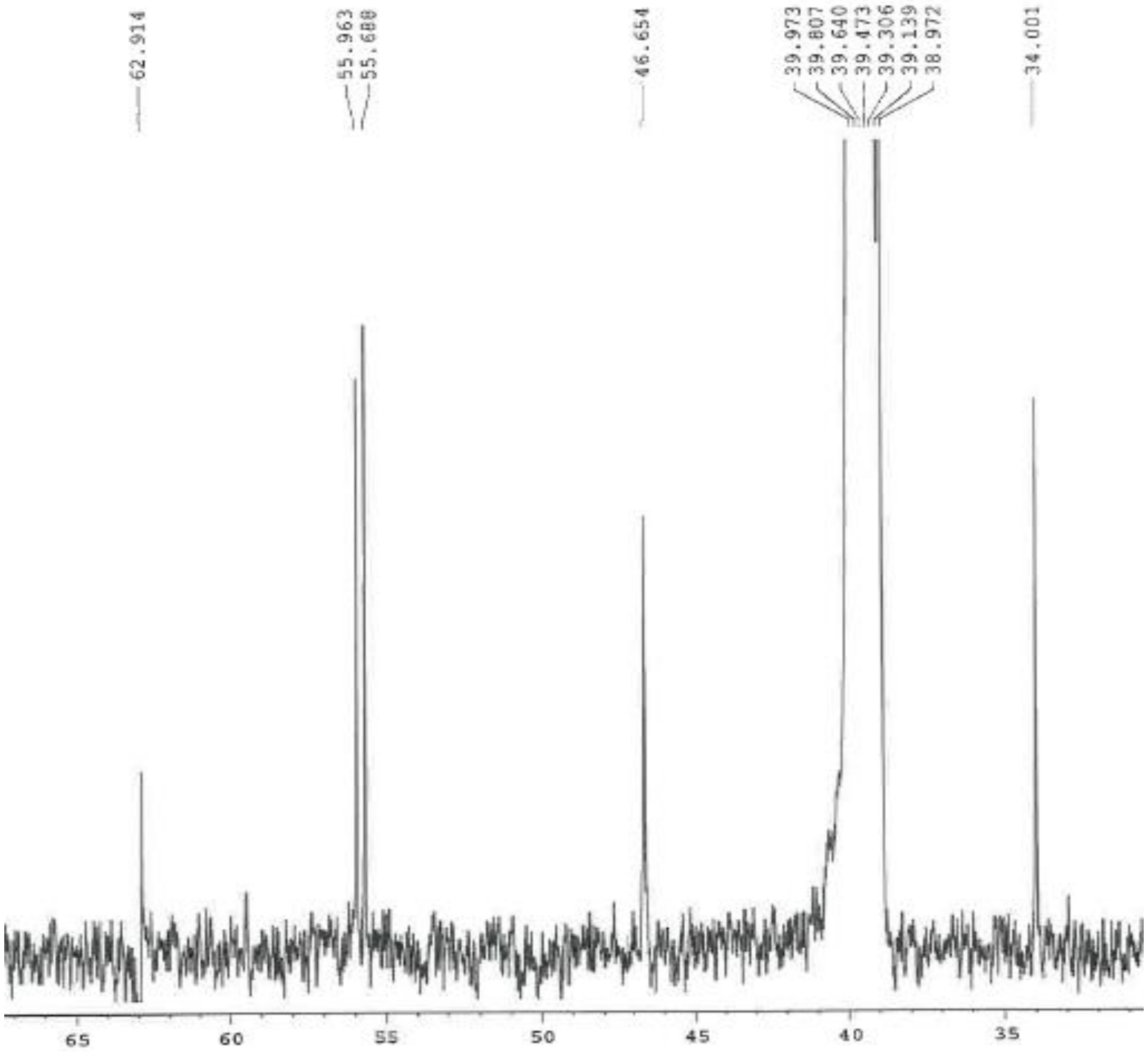

Figure S75. ^{1}HNMR of compound **3j**

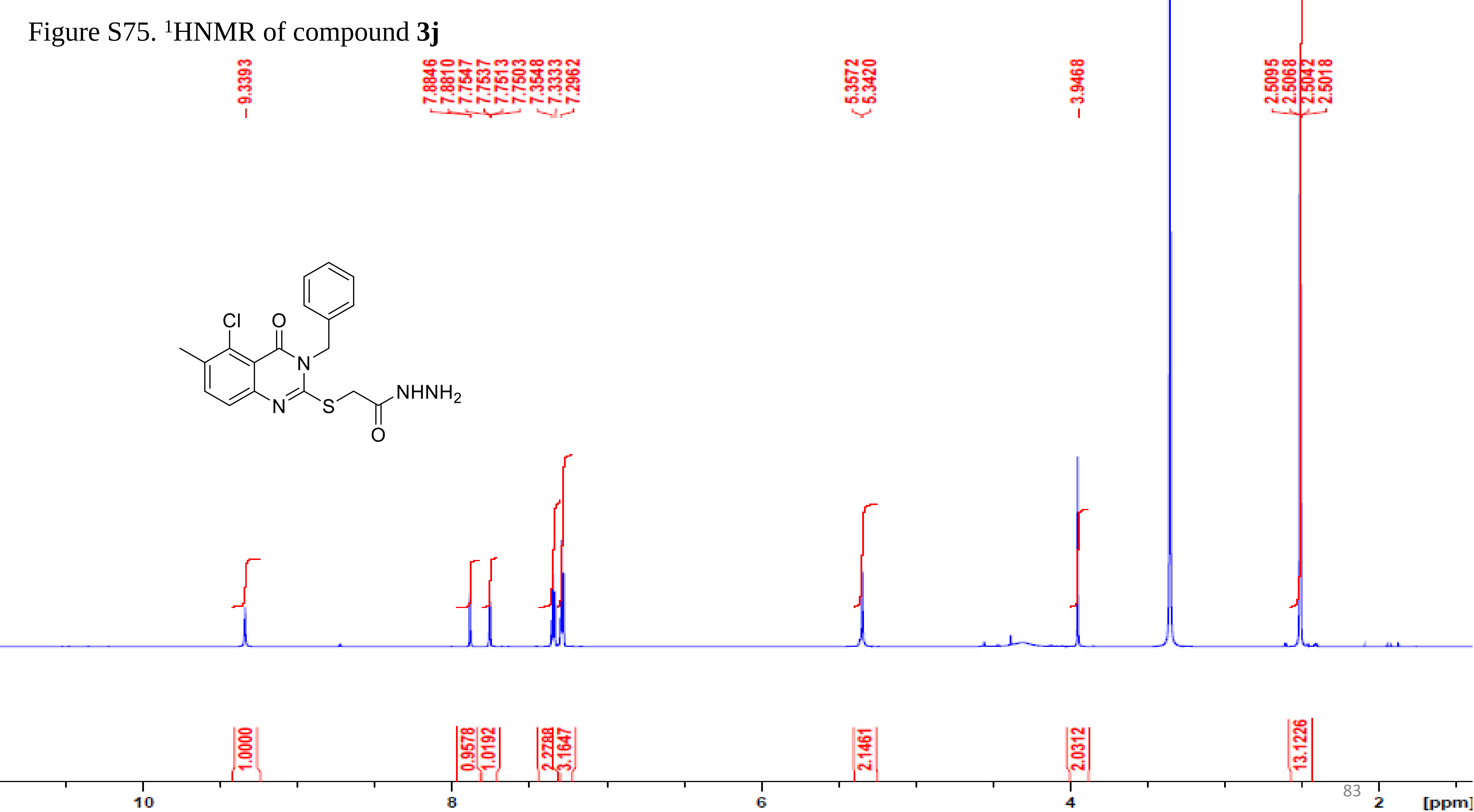

Figure S76. ^{1}HNMR of compound **3j (**extended**)**

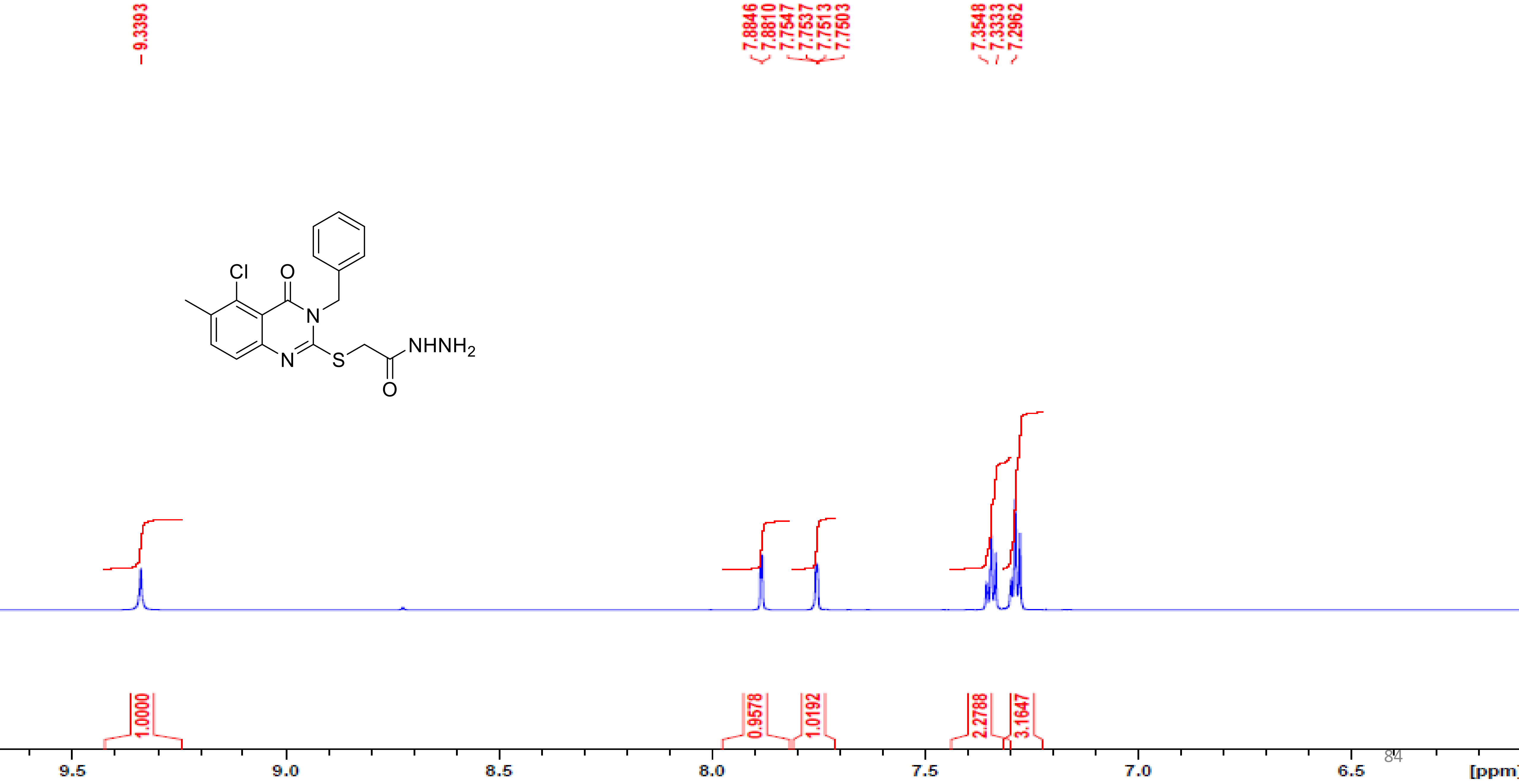

Figure S77. ^{13}CNMR of compound **3j**

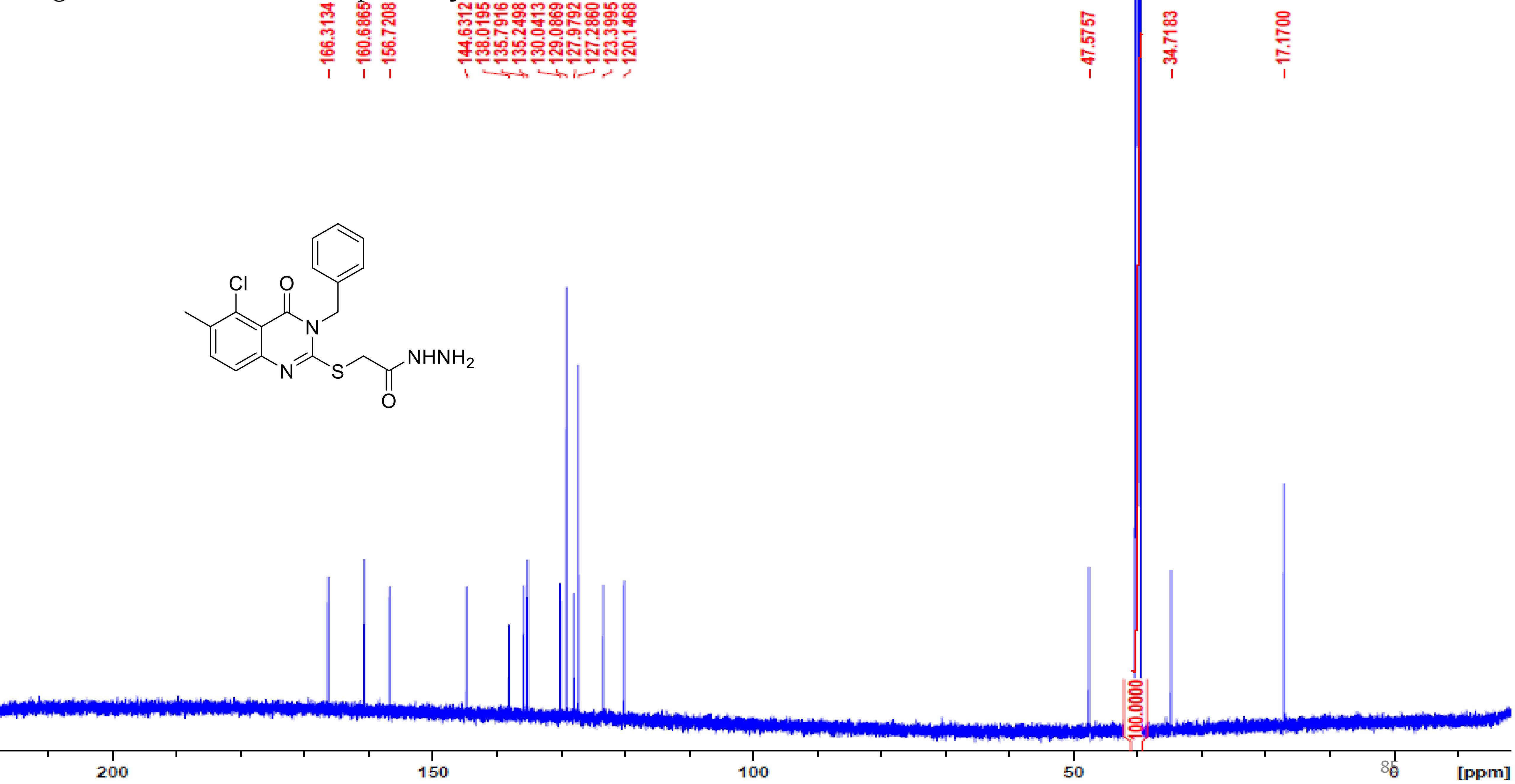

Figure S78. ^{13}CNMR of compound **3j** (extended)

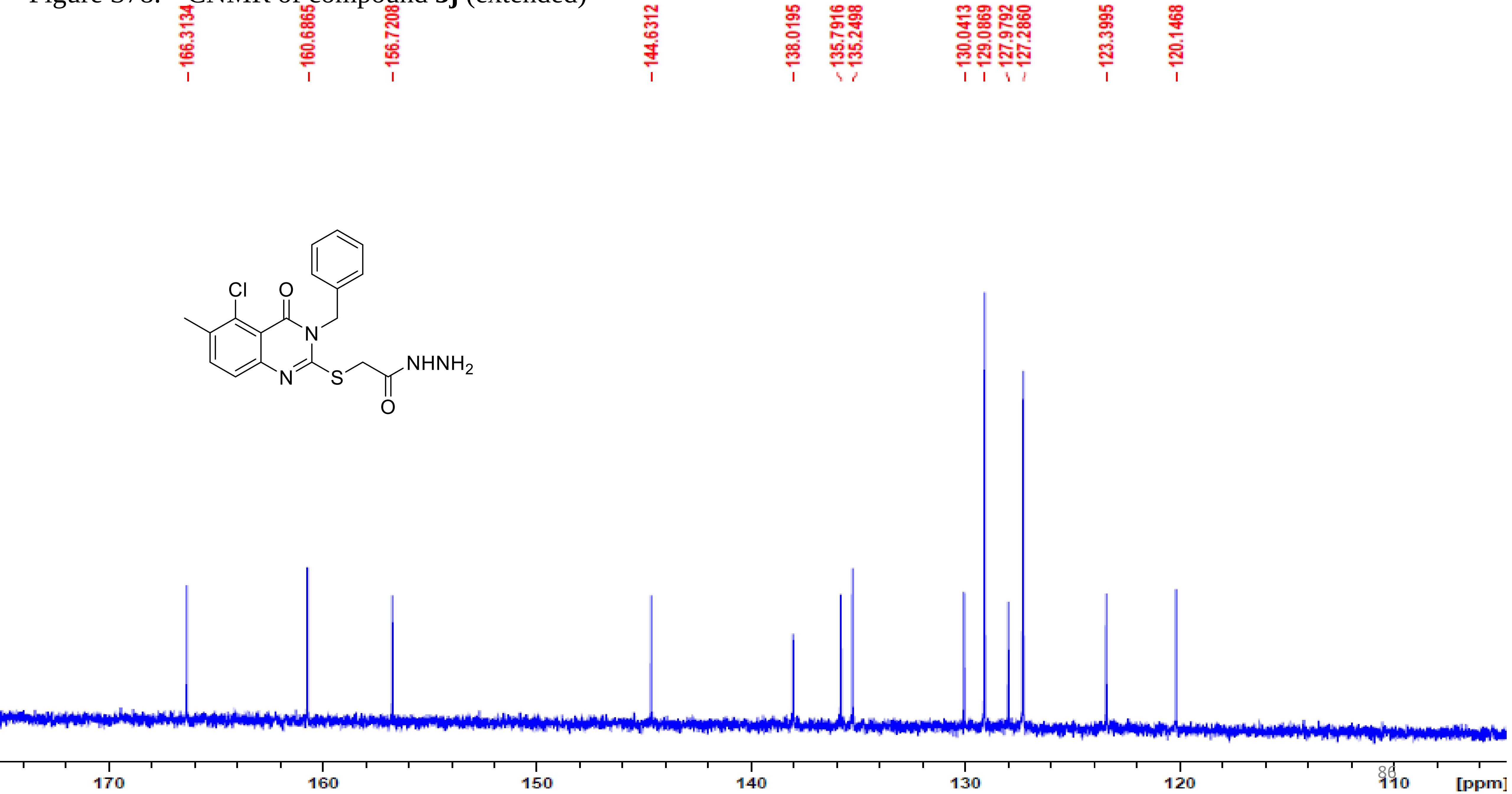

Figure S79. Docking analysis of Imatinib with CDK2 protein kinase enzymes (2D)

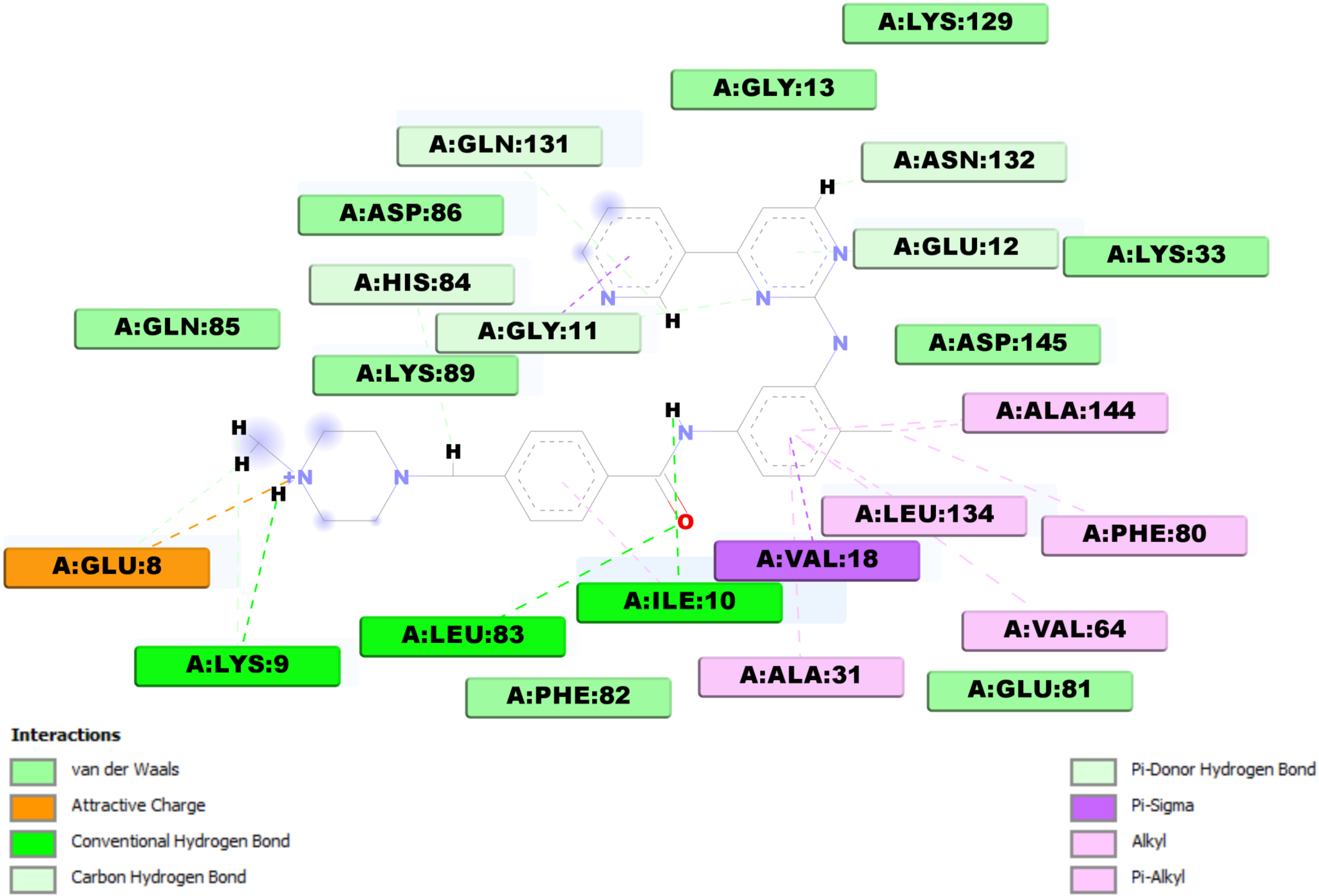

Figure S80. Docking analysis of Imatinib with CDK2 protein kinase enzymes (3D)

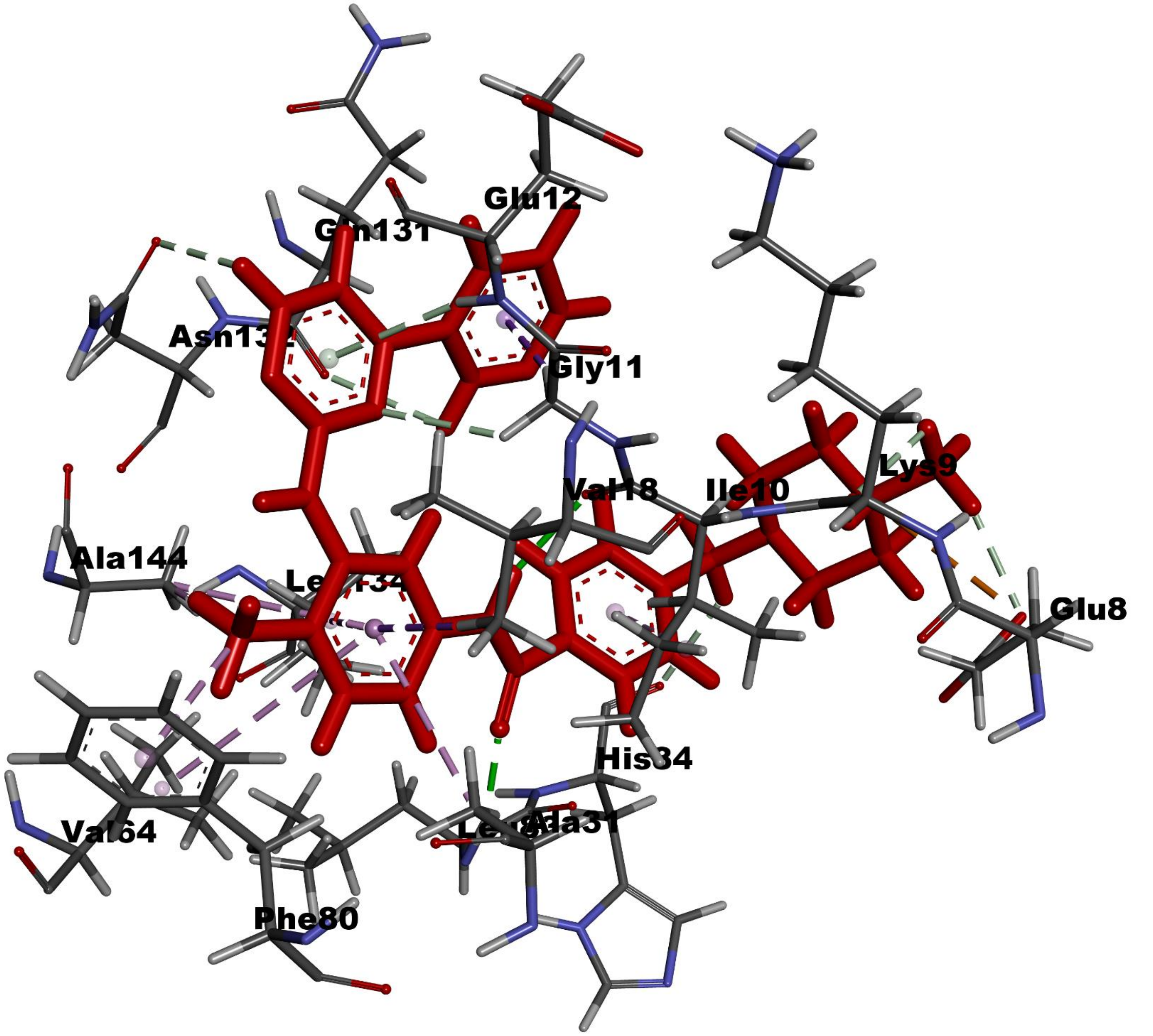

Supplement: Supplemental Material [file IENZ_A_1972992_SM9171.pdf]
